# An eQTL Analysis of Partial Resistance to *Puccinia hordei* in Barley

**DOI:** 10.1371/journal.pone.0008598

**Published:** 2010-01-06

**Authors:** Xinwei Chen, Christine A. Hackett, Rients E. Niks, Peter E. Hedley, Clare Booth, Arnis Druka, Thierry C. Marcel, Anton Vels, Micha Bayer, Iain Milne, Jenny Morris, Luke Ramsay, David Marshall, Linda Cardle, Robbie Waugh

**Affiliations:** 1 Genetics Programme, Scottish Crop Research Institute, Dundee, United Kingdom; 2 Biomathematics and Statistics Scotland (BioSS), Scottish Crop Research Institute, Dundee, United Kingdom; 3 Laboratory of Plant Breeding, Graduate School for Experimental Plant Sciences, Wageningen University, Wageningen, The Netherlands; University of Warwick, United Kingdom

## Abstract

**Background:**

Genetic resistance to barley leaf rust caused by *Puccinia hordei* involves both *R* genes and quantitative trait loci. The *R* genes provide higher but less durable resistance than the quantitative trait loci. Consequently, exploring quantitative or partial resistance has become a favorable alternative for controlling disease. Four quantitative trait loci for partial resistance to leaf rust have been identified in the doubled haploid Steptoe (*St*)/Morex (*Mx*) mapping population. Further investigations are required to study the molecular mechanisms underpinning partial resistance and ultimately identify the causal genes.

**Methodology/Principal Findings:**

We explored partial resistance to barley leaf rust using a genetical genomics approach. We recorded RNA transcript abundance corresponding to each probe on a 15K Agilent custom barley microarray in seedlings from *St* and *Mx* and 144 doubled haploid lines of the St/Mx population. A total of 1154 and 1037 genes were, respectively, identified as being *P. hordei*-responsive among the *St* and *Mx* and differentially expressed between *P. hordei*-infected *St* and *Mx*. Normalized ratios from 72 distant-pair hybridisations were used to map the genetic determinants of variation in transcript abundance by expression quantitative trait locus (eQTL) mapping generating 15685 eQTL from 9557 genes. Correlation analysis identified 128 genes that were correlated with resistance, of which 89 had eQTL co-locating with the phenotypic quantitative trait loci (pQTL). Transcript abundance in the parents and conservation of synteny with rice allowed us to prioritise six genes as candidates for *Rphq11*, the pQTL of largest effect, and highlight one, a phospholipid hydroperoxide glutathione peroxidase (HvPHGPx) for detailed analysis.

**Conclusions/Significance:**

The eQTL approach yielded information that led to the identification of strong candidate genes underlying pQTL for resistance to leaf rust in barley and on the general pathogen response pathway. The dataset will facilitate a systems appraisal of this host-pathogen interaction and, potentially, for other traits measured in this population.

## Introduction

Barley leaf rust caused by *Puccinia hordei* is a model for investigating basal disease resistance, also known as quantitative or partial resistance. *P. hordei* invades barley leaves during the entire growing season. Genetic resistance to leaf rust is common but complex, involving both major genes and quantitative trait loci (QTL). To date, 19 major race-specific leaf rust resistance genes (*R* genes named *Rph*1 to *Rph*19) have been identified [1], [2]. While these *R* genes provide high levels of resistance, they are only effective against pathogen strains carrying the cognate *Avr* genes. The effectiveness of *R* genes is limited as resistance may be quickly overcome due to loss-of-function mutations in *Avr* genes of the pathogen. Consequently, exploring quantitative or partial resistance has become a favorable alternative for controlling disease [3].

To understand the molecular basis of partial resistance, genomic regions should be identified that contain partial resistance loci. Using five different barley mapping populations, Marcel and co-workers [4] identified a total of 19 phenotypic QTL (pQTL) for partial resistance. Fourteen were found to be effective during the seedling stage, and were detected by pQTL analysis of the latency period exhibited by the rust fungus on seedling leaves. Four of these segregated in the doubled haploid Steptoe/Morex (*St/Mx*) reference mapping population. Each parent contributed the resistance allele for two of the pQTL. However, pQTL mapping alone is not sufficient to provide insight into the molecular mechanisms underpinning partial resistance which requires the molecular isolation of the causal genes. Unfortunately this is both cumbersome and time-consuming, especially if the phenotypic effects of each pQTL are relatively small.

‘Genetical genomics’ [5] provides an opportunity to elucidate the molecular processes underpinning pQTL without prior and lengthy development of pQTL isolines. This systems approach investigates the genetic determinants of transcript abundance by determining mRNA levels in the individuals of a segregating population, and analysing the observed data genetically as a quantitative trait [5]. Importantly the abundance of thousands of mRNA transcripts can be assessed simultaneously by microarray analysis in a single experiment.

The loci controlling transcript abundance have been termed expression QTL (eQTL) [6]. eQTL that map to the same genetic location as the gene whose transcript is being measured generally indicate the presence of a *cis*-acting regulatory polymorphism in the gene (*cis*-eQTL). eQTL that map distant to the location of the gene being assayed most likely identify the location of *trans*-acting regulators (*trans*-eQTL) that may control the expression of a number of genes elsewhere in the genome. eQTL analysis may therefore help to reveal networks of genes under common regulatory control. eQTL analysis also provides the possibility of correlating observed variation in the abundance of mRNA transcripts with variation observed in simple or complex phenotypes and is potentially an efficient route towards unraveling the molecular basis of phenotypic diversity [7], [8]. Importantly, several recent studies have shown that variation in transcript abundance is the cause of variation in phenotypes that include disease resistance [9], insect resistance, glucosinolate biosynthesis and activation [10]–[13], phosphate sensing [14], flowering time, circadian rhythm and plant development [15]–[18].

Many microarray studies that have been performed on crop and model plants address changes in the transcriptome during development or under biotic and abiotic stress conditions. In barley, the Affymetrix Barley1 GeneChip [19] has been employed for various studies analysing grain protein accumulation [20], senescence [21] and expression patterns during barley development [22]. The most common use has been the investigation of host-pathogen interactions involving contrasting wildtypes and mutants, and near isogenic lines exposed to infection by pathogens such as powdery mildew (*Blumeria graminis*), stem rust (*Puccinia graminis*) and head blight (*Fusarium graminearum*) [23]–[27], [11]. No published microarray studies have been performed on barley leaf rust caused by *Puccinia hordei*.

Genome-wide analyses of transcript abundance have also been performed by eQTL mapping in *Arabidopsis* [28], [29] and barley [30]. While these provide a detailed picture of transcript-level variation in the tissues studied, attempts to identify direct relationships between transcript abundance variation and phenotypes have been less successful. One notable exception was Druka *et al.* [31] who showed a very strong correlation between transcript abundance at both *Rpg1* and *Rpg4/5* loci with resistance to the wheat stem rust pathogen *Puccinia graminis* f. sp. *tritici* in barley.

In this study, we conducted an experiment to characterise quantitative resistance to the barley leaf rust pathogen *P. hordei* in the *St/Mx* population, and identify a small number of candidate genes underpinning the pQTL using a systems strategy combining genetical genomics with genetic mapping of partial resistance. We developed an Agilent barley custom microarray that we used to assess transcript abundance in 144 DH lines of the *St/Mx* population challenged with *P. hordei*. The genotypic and phenotypic datasets were generated previously by Rostoks *et al.* [32] and Marcel *et al.* [4] respectively. Correlations between transcript abundance and resistance levels, combined with genetic positional information of eQTL and pQTL allowed us to prioritise a small number of candidate genes for further study.

## Results

### Fungal Development across the Time Points Post Inoculation

**Figure 1:**
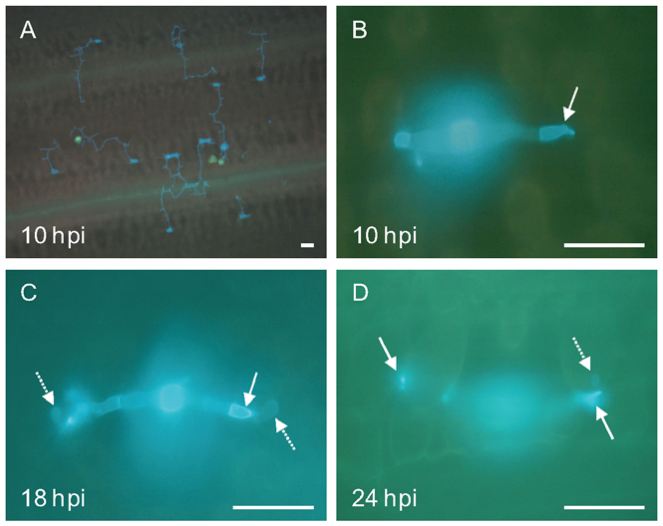
Micrographs viewed under epi-fluorescence microscopy after staining with Uvitex, showing development of *P. hordei* at different time points post inoculation. A: overview of germinating urediospores on barley leaves 10 hpi, green spots are inert spores of lycopodium; B, C and D: close-up images showing infection units at10, 18 and 24 hpi, respectively. Solid arrows indicate haustorial mother cells, dotted arrows haustoria. Scale bar = 50 µm.

Previous studies indicated that both *St* and *Mx* have similar levels of resistance, both containing resistance and susceptibility alleles at pQTL [4]. Our microscopic investigation of the timing of pathogen development on the two parents revealed no observable differences. Urediospore germination occurred within 10 hpi on leaf surfaces by producing a germ tube that grew towards the stoma on which it formed an appressorium (Figure 1A). By 10 hpi, a penetration peg had entered the stoma and had formed a torpedo-shaped substomatal vesicle in the substomatal space. At this stage haustorial mother cells (HMCs) were clearly visible but haustoria were not yet formed (Figure 1B). At 18 hpi, 61% of infection units had penetrated the host cells and developed haustoria from the tips of HMCs, indicating colonisation. At 24 hpi 85% of the infection units had formed at least one haustorium (Figure 1D). Thereafter, infection hyphae extended inter-cellularly to attack neighbouring mesophyll cells by forming new HMCs and intracellular haustoria, ultimately followed by pustule formation and completion of the life cycle (images not shown). As studies with other biotrophic pathosystems have shown that expression divergence between compatible and incompatible interactions occurs during membrane-to-membrane contact after cell wall (as opposed to stoma) penetration and during early haustorial development [23], [43], we chose 18 hpi for tissue sampling. Niks [44] observed that partial resistance of barley to *P. hordei* is associated with a substantial amount of failed haustorium formation at about 24 hours after inoculation.

### Analysis of *Ph*-Responsive (Induced/Suppressed) Genes

Comparisons between *Ph*-infected and mock-inoculated controls were made to identify *Ph*-responsive genes. Respectively, 935 and 844 probes detected significant transcript abundance changes from *St* and *Mx* with 698 up-regulated and 237 down-regulated for *St*, and 603 up-regulated and 241 down-regulated for *Mx* (Figure S1). In total, 1154 probes recorded differential transcript abundance and were considered to represent *Ph*-responsive genes. Of the 1154 probes, 625 indicated significant *Ph*-responsive gene expression in *St* as well as in *Mx* and showed the same manner of regulation (up or down) in response to *Ph*-infection in both parental lines. Table S3 shows the complete list of differentially expressed genes with their expression levels, corresponding *p*-values and putative functional annotation based on HarvEST:Barley (http://harvest.ucr.edu/). The putative function of each gene was examined and grouped into the twelve major categories shown in Figure S2. Genes in the defense responsive categories were predominantly up-regulated, whereas genes involved in cell wall structure and light harvesting were mostly down-regulated (Figure S2, and Table S3). Gene ontology enrichment analysis using the web-based tool GOEAST (see Materials and Methods) revealed that the *Ph*-responsive genes were significantly enriched (*p*<0.05) for those classified as controlling response to stimulus (including two sub-branches of response to biotic stimulus and stress), cell wall organization, protein transport, L-phenylalanine catabolic process and glucan metabolic process (Table S2). Not unexpectedly, this confirms that many *Ph*-responsive genes are functionally associated with defense and that at 18 hpi the defense response has clearly been initiated.

### Analysis of Differential Expression between Parental Lines

Comparison of transcript abundance between the two *Ph*-infected parental lines identified 1037 probes reporting significantly differentially expressed genes (Table S4). A similar number of genes showed higher transcript abundance in *St* (514) as in *Mx* (523). Of the 1037 probes, 206 were also *Ph*-responsive genes (61 from *St*, 52 from *Mx* and 93 from both parental lines) (Figure S1).

### eQTL Analysis

#### Maximizing informative comparisons for eQTL analysis

**Figure 2:**
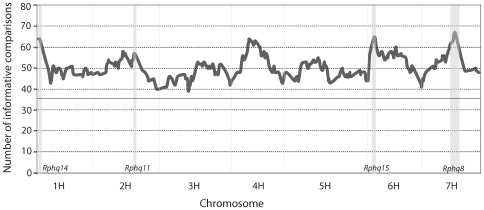
Number of informative comparisons across the barley genome based on a distant-pair design (see text) with extra weight given to four pQTL regions (shown as grey blocks). The solid horizontal line at 36 represents the average number of informative comparisons when samples were randomly paired.

We adopted an optimal distant pair design [36] to maximize the informative comparisons for eQTL analysis from the minimum number of microarrays. Genetic distances between the 144 DH lines in the *St/Mx* population were analyzed using SNP genotypic data. We derived 72 pairs that maximized the overall genetic difference. Figure 2 shows the informative number of comparisons across the whole genome. Using this distant-pair design, the informative pairs increased from an average of 36 out of 72 pairs in random pairing to an overall average of 50 with the highest number of informative pairs (64, 57, 64, and 66) at the four QTL regions *Rphq14*, *11*, *15* and *8* respectively, where extra weight was given in the distant pair analysis.

**Figure 3:**
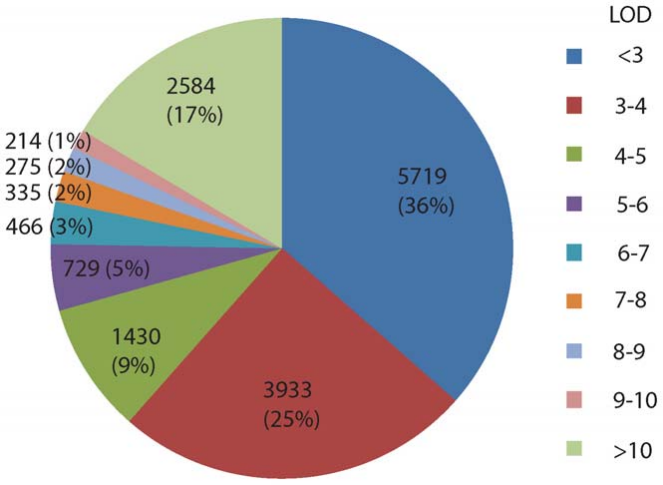
Numbers and proportions of eQTL with different *LOD* scores. A total of 15685 significant eQTL (*p*<0.001) was detected with *LOD* scores ranging from 2.4 to 55.7.

As transcript abundance variation in a segregating population may be detected for genes that are not differentially expressed between the parental lines (due to transgressive segregation), we carried out regression analysis of transcript abundance represented by all of the 15208 probes on the microarray against all 466 SNP markers. In total, 9557 probes (62.8%) detected significant (*p*<0.001) associations between transcript abundance and one to six SNP markers at distinct genomic regions. This corresponds to a total of 15685 eQTL. Of these 9557 probes, 916 represented *Ph*-responsive genes. Summaries of the numbers and proportions of eQTL with respect to their *LOD* scores, and partitioning into classes discussed above, are displayed in Figure 3 and Table 1.

**Table 1:** Number of significant eQTL (*p*<0.001) and genes in different groups.

Number of	Overall		*Ph*-responsive		SNP-mapped		TDM-mapped	
eQTL/gene	genes	eQTL	genes	eQTL	genes	eQTL	genes	eQTL
0	5651	0	238	0	-	-	-	-
1	5103	5103	361	361	135	135	653	653
2	3074	6148	314	628	73	146	296	592
3	1122	3366	184	552	36	108	84	252
4	227	908	48	192	8	32	29	116
5	26	130	7	35	1	5	4	20
6	5	30	2	12	0	0	0	0
**total**	**9557**	**15685**	**916**	**1780**	**253**	**426**	**1066**	**1633**

#### Analysis of eQTL from genes with known map positions

**Figure 4:**
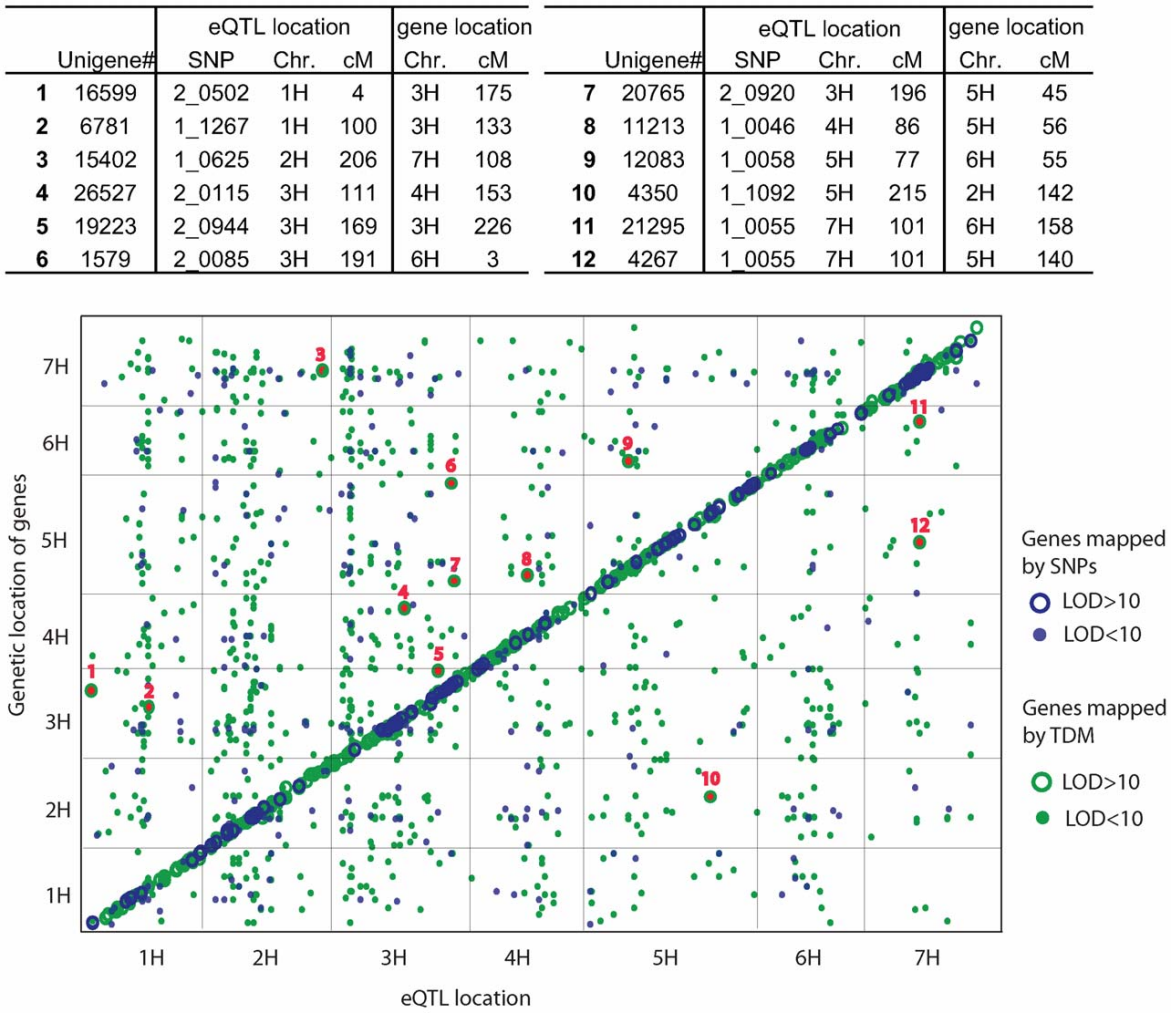
Overview of eQTL mapping results for genes previously mapped by SNP and TDM markers. The *x*-axis shows the locations of eQTL associated with transcript abundance from the current experiment. The *y*-axis shows the location of genes mapped previously as SNPs (253 genes, [29]), TDMs (1066 genes, [31]) or both (63 genes). The 1256 previously mapped genes correspond to 1623 eQTL in the present study. eQTL corresponding to SNP- and TDM- mapped genes are displayed in blue and green respectively. eQTL with *LOD* score>10 and <10 are distinguished as circles or dots. Circles and dots on the diagonal represent correspondence between the locations observed in the current study with previous work [29], [31]. Circles or dots off the diagonal represent *trans*-eQTL. While all eQTL and their corresponding SNP-mapped genes were on the diagonal, 12 eQTL with LOD>10 (highlighted as numbered and red-filled green circles) when compared to TDM-mapped genes were located at distinctly different (>25cM away) positions.

**Figure 5:**
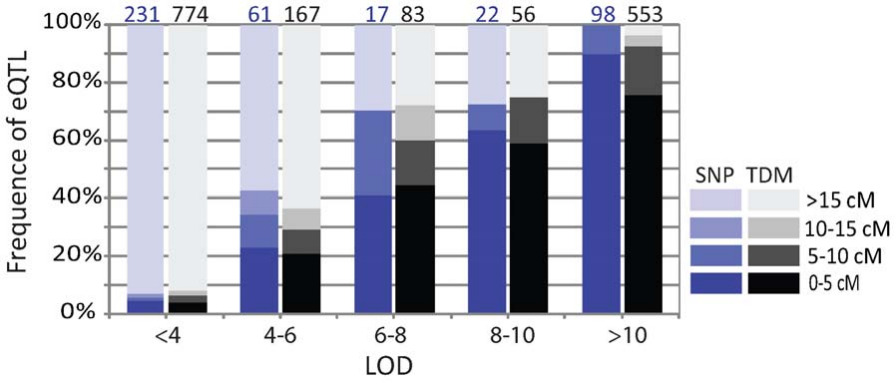
Relationship between eQTL *LOD* scores and position relative to their corresponding genes. Numbers on the top of the columns are the total number of genes mapped by SNP (blue) and TDM (black). The colour key represents different eQTL categories assigned according to distance from their corresponding genes.

Of the 9557 genes that were described by one or more eQTL, 253 had previously been mapped using coding sequence SNPs [32] and 1066 as transcript-derived markers (TDMs) [45], [30]. This represented a total of 1256 uniquely mapped genes as 63 of these were mapped as both SNPs and TDMs. These 1256 genes/probes revealed 1623 significant eQTL. Plotting the position of eQTL-associated markers against the position of their corresponding genes revealed significant eQTL-by-gene association across the genome (Figure 4). It has been reported previously that high *LOD* eQTL are frequently located close to their corresponding genes [46], [47], [28], [30]. We therefore analysed the relationship between eQTL *LOD* scores and their correspondence with structural gene locations in more detail. We superimposed the *LOD* scores of individual eQTL onto the distances observed between the location of the previously mapped SNPs and TDMs and their corresponding eQTL (Figure 5). We observed that as eQTL *LOD* scores increase, a higher frequency co-locate with their corresponding SNP or TDM locus. Ultimately, eQTL with *LOD*>10 were all (for SNP-mapped genes) or nearly all (93%, TDM-mapped genes) detected within 10 cM of their corresponding genes (Figure 5). Of the 7% (*i.e.* 40 eQTL) that were more than 10 cM away from their corresponding TDMs, 28 were located within 25 cM on the same chromosome, and 12 were further than 25 cM or on different chromosomes (Figure 4).

#### Analysis of eQTL for the Ph-responsive genes

**Figure 6:**
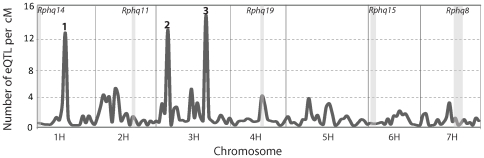
eQTL density for *Ph*-responsive genes across the genome. *x*-axis, eQTL genomic location on chromosomes. *y*-axis, eQTL density calculated on a 10 cM sliding window. Chromosomal regions corresponding to the three most significant peaks are named as eQTL hotspots 1, 2 and 3 from left to right. The five pQTL regions were indicated by the grey blocks.

Comparisons between *Ph*-infected treatments and mock controls identified 1154 genes that were *Ph*-responsive in at least one of the two parents. Of these, 916 had one or more significant eQTL, yielding a total of 1780 eQTL for *Ph*-responsive genes (Table 1 and Table S5). To investigate if the eQTL for *Ph*-responsive genes were randomly distributed across the genome, or clustered as eQTL hot spots, we calculated the density of eQTL per cM across the genome using 10 cM sliding window analysis (Figure 6). Three regions had a high eQTL density centering around SNP markers 2_1057 (98 cM on Chr. 1H), 1_0571 (18 cM on Chr. 3H) and 2_0023 (153 cM on Chr. 3H), each having over 12 eQTL per cM, in contrast to 1.2 if the 1780 eQTL were evenly distributed along the 1533 cM genetic linkage map. These three 10cM intervals harboured 127, 134 and 151 eQTL for *Ph*-responsive genes. The same regions contained 11, 17 and 23 genes that were previously mapped by SNPs and TDMs [30], [32] corresponding to eQTL/gene ratios of 11.5, 7.9 and 6.6 respectively as compared to 0.64 (1780 eQTL *vs* 2776 genes in total) on average. The three regions were therefore named as eQTL hotspots 1, 2 and 3 respectively. To investigate if the members of each eQTL hotspot shared a common biological function (*e.g.* metabolic pathways or similar gene ontology functional annotation), the *Ph*-responsive genes located within each hotspot were separately subjected to GO enrichment analysis. Hotspot 1 was overrepresented by genes that are involved in GO term ‘response to stimulus’, and all of its sub-categories and a few GO terms in ‘metabolic process’. Hotspot 2 was over represented by genes with GO classifications ‘cellular process and localization’, ‘response to stimulus’ and ‘metabolic process’ (Table S2). No GO classes of genes were found to be significantly overrepresented for hotspot 3. None of the eQTL hotspots co-located with known pQTL for *Ph*-resistance.

### pQTL for Partial Resistance and Correlations between Transcript Abundance and Rust Resistance

Four pQTL for leaf rust resistance at the seedling stage have previously been identified in *St/Mx* and named *Rphq8*, *11*, *14*, and *15* [4]. We re-analysed the phenotypic resistance data of Marcel *et al.* [4] using the same model that we used for eQTL analysis after converting the RLP50S phenotypic scores into ratios calculated for each of the distant pairs. This provided a phenotypic data set that was consistent with the transcript abundance data set. We found that the SNP marker 1_0649 (142 cM on Chr. 2H) was associated most strongly with rust resistance (*R^2^* = 35.3%) (resistance allele derived from *St*). We then tested for further associations with a two marker model, testing each other marker together with the marker 1_0649 from Chr. 2H. This identified the following four SNPs: 2_1032 (14 cM on Chr. 6H, *R^2^* = 12.0%), 1_1513 (106 cM Chr. 4H, *R^2^* = 10.1%), 2_1174 (13 cM Chr. 1H, *R^2^* = 7.6%) and 1_0431 (91 cM Chr.7H, *R^2^* = 11.7%) as most significant (*p*<0.005) with the resistance alleles being derived from *St* for pQTL at 2_1032 and *Mx* for the other three. Multiple regression analysis indicated that these five pQTL, together, accounted for a total of 62% of the phenotypic variance. Four of these five markers (1_0649, 2_1032, 2_1174 and 1_0431) were located within the pQTL regions previously identified as *Rphq11*, *15*, *14* and *8* respectively. The SNP marker 1_1513 on Chr. 4H, indicated a pQTL not previously reported in the *St/Mx* population, being marginally below the significance threshold (T.C. Marcel, unpublished data). As this locus corresponds with the location of *Rphq19*, a pQTL previously detected in the Oregon Wolf Barley (OWB) DH population, we refer to this pQTL as *Rphq19*.

We next performed correlation analysis between the transcript abundance ratios recorded at each probe and resistance score ratios from corresponding sample pairs. We identified 128 probes on the microarray that reported transcript abundance ratios that were significantly correlated with rust resistance (*p*≤0.001). Six of these were previously classified as *Ph*-responsive. We then made positional comparisons between the eQTL associated with these probes and the aforementioned five pQTL for rust resistance. Of the 128-probe transcript abundance datasets, four revealed no significant eQTL and thirty-five had eQTL that were located outside the five resistance pQTL regions. Twenty-five of the latter were located within one of the three eQTL hotspots from the *Ph*-responsive genes. Based on this locational information, these 39 probes were not considered further. The remaining 89 probes revealed 95 significant eQTL (2 eQTL were detected for 6) located in the five pQTL regions with 1, 54, 4 and 26 being within the confidence intervals of the four previously reported pQTL *Rphq14* (Chr. 4H), *Rphq11* (Chr. 2H), *Rphq15* (Chr.6H) and *Rphq8* (Chr. 7H) respectively, and 10 within a 10 cM interval around *Rphq*19 (Table S6). eQTL for the 22 genes that were most significantly correlated with resistance (*p*<10^−4^) all mapped to *Rphq11*. In this report we therefore focus further analysis only on this pQTL.

### Candidate Genes for *Rphq11*

To identify the most promising candidate genes for *Rphq11*, we first analyzed conservation of synteny in the region surrounding *Rphq11* with the rice genome sequence. The objective was to determine if the genes represented by these 54 probes were likely to be physically co-located in this region. A BLASTN search for rice homologues of the consensus EST sequences represented by the 54 probes identified 31 that were located at a conserved syntenic position corresponding to 27–30 Mb on rice chromosome 4. Seventeen were located elsewhere in the rice genome and six (unigenes 17168, 18410, 15816, 17152, 3199, and 20160) revealed no significant rice homologs (*E* value of <1*e*-10). Of the 31 genes located at conserved syntenic positions, 25 were detected as *cis-*eQTL with *LOD*>10, supporting their physical and genetic co-location with *Rphq11* (Table 2).

**Table 2:** Candidate gene analysis of the 54 genes with eQTL detected at *Rphq11*.

Unigene ID	Rice homologue	eQTL		Correlation with resistance		*Ph*-infected		Response to Ph-infection		Response to Ph-infection		Annotation
		SNP	LOD	*r*	*p*-value	*Mx*	*St*	infected-*Mx*	mock-*Mx*	infected-*St*	mock-*St*	
**genes without rice homologue with rice homologues located in the synteny corresponding to ** ***Rphq11***												
**UNIGENE2453**	**LOC_Os04g46960.2**	**1_0649**	**40.8**	**0.59**	**4.39889E-08**	**1109±176**	2394±58*	**764±83**	**739±40**	**1963±102**	**1832±167**	**phospholipid hydroperoxide glutathione peroxidase**
**UNIGENE7644**	**LOC_Os04g45910.1**	**1_0475**	**27.3**	**0.52**	**2.7986E-06**	**425±34**	979±88*	**496±39**	**409±47**	**1104±121**	**793±71**	**placental protein 11 precursor**
**UNIGENE14456**	**LOC_Os04g47280.1**	**2_1007**	**37.4**	**−0.53**	**1.39292E-06**	**1172±87**	403±60*	**1226±52**	**1375±55**	**364±36**	**463±20**	**alpha-L-fucosidase 1 precursor**
**UNIGENE15522**	**LOC_Os04g46190.3**	**1_0936**	**36.8**	**−0.50**	**8.52479E-06**	**2799±175**	1394±118*	**3117±134**	**3458±218**	**1531±62**	**1584±59**	**ubiquitin carboxyl-terminal hydrolase isozyme L3**
**UNIGENE15767**	**LOC_Os04g48840.1**	**1_0969**	**27.1**	**−0.44**	**0.000113711**	**1392±104**	517±93*	**1269±103**	**1997±51**	**481±78**	**731±93**	**protein kinase**
**UNIGENE10081**	**LOC_Os04g49350.1**	**1_0936**	**32.4**	**−0.44**	**0.000131026**	**811±50**	160±27*	**921±55**	**936±90**	**162±28**	**215±34**	**expressed protein**
UNIGENE12856	LOC_Os04g46750.1	1_0649	22.1	−0.53	1.90626E-06	272±12	150±17	227±17	224±6	125±3	135±15	transferase transferring glycosyl groups
UNIGENE20372	LOC_Os04g48520.1	1_0214	18.5	−0.47	2.56925E-05	898±58	901±38	984±51	1050±35	869±26	962±44	expressed protein
UNIGENE2970	LOC_Os04g47690.3	1_0214	11.6	−0.43	0.000190821	11530±1277	6655±402	9838±805	11394±471	6170±471	7621±488	HMG1/2-like protein
UNIGENE19081	LOC_Os04g45860.2	1_0475	14.2	−0.42	0.000244338	817±49	485±23	1008±76	1384±79	607±40	957±74	transposon protein
UNIGENE17934	LOC_Os04g48750.2	1_0969	21.7	−0.41	0.000375063	2008±174	1578±110	1828±97	1806±38	1426±96	1450±52	3-oxo-5-alpha-steroid 4-dehydrogenase
UNIGENE26389	LOC_Os04g46610.2	1_0649	16.0	−0.39	0.000676861	5623±1064	3702±885	4442±633	6089±380	3156±808	4416±828	PAP fibrillin family protein expressed
UNIGENE14163	LOC_Os04g48510.1	1_0969	22.5	−0.44	0.000848567	54±21	21±11	71±7	119±12	9±2	20±5	transcription activator
UNIGENE25788	LOC_Os04g51050.2	1_0475	5.0	0.38	0.001010222	203±28	233±48	194±28	178±32	246±39	229±59	OsWAK receptor-like protein kinase
*UNIGENE9814*	*LOC_Os04g48160.1*	*1_0214*	*26.5*	*−0.51*	*4.80738E-06*	*279±50*	72±10*	*276±19*	*160±18*	*52±8*	*33±13*	*calmodulin binding protein*
*UNIGENE23650*	*LOC_Os04g48790.1*	*1_0214*	*5.5*	*0.43*	*0.000166834*	*39±7*	90±7*	*39±8*	*42±3*	*88±16*	*106±15*	*rac GTPase activating protein 2*
*UNIGENE11212*	*LOC_Os04g49194.1*	*1_0936*	*13.4*	*−0.39*	*0.000813902*	*12940±1368*	3194±249*	*10478±1005*	*3595±935*	*2234±206*	*549±58*	*naringenin2-oxoglutarate 3-dioxygenase*
*UNIGENE11646*	*LOC_Os04g46930.2*	*1_0649*	*24.3*	*0.58*	*1.20566E-07*	*691±51*	*1257±33*	*730±29*	*1040±78*	*1632±55*	*1853±87*	*serine racemase*
*UNIGENE20757*	*LOC_Os04g47680.1*	*1_0214*	*13.7*	*−0.54*	*1.16612E-06*	*1159±59*	*854±74*	*1378±115*	*1173±65*	*1081±46*	*875±69*	*Ser/Thr-rich protein T10 in DGCR region*
*UNIGENE6071*	*LOC_Os04g47120.1*	*1_0649*	*26.8*	*0.53*	*1.36383E-06*	*10712±967*	*13860±1688*	*11665±571*	*12826±889*	*15704±851*	*16339±1019*	*acyl-CoA thioesterase/cyclic nucleotide binding protein*
*UNIGENE1225*	*LOC_Os04g47220.1*	*2_1007*	*17.6*	*0.51*	*4.06056E-06*	*38251±8433*	*55030±11560*	*33434±5490*	*50870±7692*	*48369±7446*	*59404±8124*	*aquaporin PIP1.2*
*UNIGENE13970*	*LOC_Os04g48140.1*	*1_0214*	*27.1*	*−0.51*	*4.09792E-06*	*1124±123*	*667±73*	*1063±87*	*1080±56*	*586±42*	*508±42*	*methyltransferase family protein*
*UNIGENE12601*	*LOC_Os04g47820.1*	*1_0649*	*18.6*	*−0.51*	*5.19895E-06*	*967±109*	*734±52*	*929±66*	*973±71*	*700±35*	*651±71*	*expressed protein*
*UNIGENE1242*	*LOC_Os04g47220.1*	*2_1007*	*26.0*	*0.52*	*5.5318E-06*	*0±2*	*33±7*	*-*	*-*	*66±10*	*101±16*	*aquaporin PIP1.2*
*UNIGENE1237*	*LOC_Os04g47220.1*	*2_1007*	*20.9*	*0.52*	*5.79476E-06*	*16±7*	*88±13*	*8±3*	*27±3*	*118±15*	*166±20*	*aquaporin PIP1.2*
*UNIGENE1235*	*LOC_Os04g47220.1*	*2_1007*	*17.5*	*0.49*	*1.44965E-05*	*24748±5170*	*37463±6678*	*22944±3732*	*36280±3652*	*35876±4882*	*45537±3980*	*aquaporin PIP1.2*
*UNIGENE12506*	*LOC_Os04g45800.1*	*1_0475*	*29.3*	*−0.48*	*1.9828E-05*	*1047±96*	*543±25*	*1113±33*	*1040±57*	*529±8*	*462±38*	*sphingosine kinase*
*UNIGENE20608*	*LOC_Os04g47300.1*	*1_0475*	*4.0*	*0.45*	*6.48563E-05*	*782±63*	*953±136*	*812±99*	*1114±108*	*1083±158*	*1427±164*	*calcium-dependent protein kinase*
*UNIGENE21087*	*LOC_Os04g46570.3*	*2_1007*	*7.4*	*−0.44*	*9.32753E-05*	*332±26*	*266±32*	*345±4*	*294±16*	*305±26*	*281±44*	*growth regulator like protein*
*UNIGENE22118*	*LOC_Os04g47270.1*	*2_1007*	*8.5*	*0.42*	*0.000249507*	*479±69*	*592±73*	*527±53*	*680±56*	*679±31*	*874±55*	*flowering locus D*
*UNIGENE11370*	*LOC_Os04g46100.1*	*1_0475*	*8.0*	*−0.39*	*0.000729696*	*2414±329*	*1708±219*	*2932±256*	*3005±269*	*2316±148*	*2249±171*	*expressed protein*
**genes without rice homologue or with rice homologues located outside the synteny corresponding to ** ***Rphq11***												
UNIGENE17168	No hits found	1_0649	23.7	−0.50	9.47247E-06	199±27	94±20*	279±35	285±26	168±33	215±58	No description
UNIGENE8616	LOC_Os03g53310.1	2_1007	31.7	−0.47	3.01711E-05	5779±430	225±29*	6613±207	5755±373	172±47	230±100	suppressor/enhancer of lin-12 protein 9 precursor
UNIGENE12147	LOC_Os02g52010.1	1_0475	7.8	0.39	0.000648413	2446±330	6716±773*	2081±363	1816±594	6441±889	3768±806	phi-1-like phosphate-induced protein
UNIGENE18410	No hits found	1_0475	21.1	−0.45	7.36746E-05	3613±280	2762±262	3764±193	4523±308	3048±229	3712±212	No description
UNIGENE9767	LOC_Os10g18370.1	1_0649	2.7	−0.43	0.000140629	7258±553	7473±843	7302±163	7179±236	7221±566	8084±1231	uncharacterized ACR COG1678 family protein
UNIGENE5255	LOC_Os03g07570.1	1_0936	2.5	−0.38	0.000929807	1302±85	1388±251	1678±230	1778±238	1874±192	2033±350	alanine–glyoxylate aminotransferase 2
UNIGENE15816	No hits found	1_0475	14.1	0.38	0.000968049	173±28	231±37	166±27	54±5	273±23	79±10	No description
UNIGENE2253	LOC_Os04g18090.1	1_0969	7.1	−0.38	0.000984173	10599±1335	7365±74	9277±1016	11574±306	6722±413	10401±1110	histone H1
*UNIGENE7845*	*LOC_Os04g59360.1*	*2_1007*	*40.9*	*0.54*	*1.17112E-06*	*374±25*	1513±159*	*328±6*	*377±14*	*1595±67*	*1726±116*	*PR5*
*UNIGENE6615*	*LOC_Os04g59360.1*	*2_1007*	*44.7*	*0.52*	*3.5913E-06*	*195±38*	3908±213*	*26±4*	*20±4*	*4046±607*	*4115±602*	*PR5*
*UNIGENE20160*	*No hits found*	*1_0214*	*22.9*	*−0.47*	*2.56601E-05*	*360±52*	110±25*	*362±46*	*503±63*	*134±24*	*126±20*	*No description*
*UNIGENE19492*	*LOC_Os08g20440.1*	*2_1007*	*3.0*	*0.41*	*0.000349971*	*893±109*	390±54*	*884±55*	*940±30*	*525±93*	*570±63*	*expressed protein*
*UNIGENE5562*	*LOC_Os01g56330.1*	*1_0475*	*5.4*	*0.47*	*3.5641E-05*	*732±63*	*1160±102*	*725±35*	*971±117*	*1287±112*	*1739±160*	*protein kinase*
*UNIGENE12510*	*LOC_Os03g22490.4*	*1_0649*	*2.5*	*−0.45*	*8.72925E-05*	*1656±101*	*2537±338*	*1703±119*	*1281±43*	*2773±63*	*2235±175*	*heavy metal-associated domain containing protein*
*UNIGENE25228*	*LOC_Os11g24060.1*	*1_0475*	*5.3*	*−0.44*	*0.000102949*	*1738±261*	*2693±495*	*1628±292*	*2019±259*	*2724±420*	*2703±341*	*transmembrane transport protein*
*UNIGENE2581*	*LOC_Os12g32240.1*	*1_0649*	*3.4*	*−0.42*	*0.00020512*	*2330±241*	*2293±151*	*1991±60*	*1714±56*	*2098±100*	*1695±186*	*eukaryotic translation initiation factor 5A-2*
*UNIGENE7391*	*LOC_Os02g45180.1*	*2_1007*	*5.1*	*−0.40*	*0.000461853*	*4794±415*	*4407±271*	*3976±122*	*2787±137*	*3567±60*	*2311±43*	*ORM1-like protein 2*
*UNIGENE16687*	*LOC_Os03g12250.1*	*1_0936*	*2.7*	*0.40*	*0.000567601*	*516±87*	*586±112*	*553±94*	*765±94*	*625±123*	*642±106*	*atypical receptor-like kinase MARK*
*UNIGENE3383*	*LOC_Os01g42860.1*	*1_0969*	*4.7*	*−0.39*	*0.000608069*	*2415±328*	*3237±655*	*2480±361*	*1303±159*	*2991±405*	*1625±198*	*protein subtilisin-chymotrypsin inhibitor 2,*
*UNIGENE3199*	*No hits found*	*1_0969*	*2.8*	*0.39*	*0.000682439*	*30950±3314*	*36651±7226*	*26778±1779*	*30374±4413*	*33570±1915*	*35833±4626*	*No description*
*UNIGENE14421*	*LOC_Os04g36820.1*	*2_1007*	*5.2*	*0.39*	*0.000811315*	*157±36*	*220±54*	*124±15*	*210±19*	*238±44*	*396±51*	*hypothetical protein*
*UNIGENE19205*	*LOC_Os01g12770.1*	*1_0475*	*4.0*	*−0.39*	*0.000875264*	*26±3*	*41±14*	*30±10*	*21±5*	*72±20*	*24±5*	*cytochrome P450 71A26*
*UNIGENE17152*	*No hits found*	*1_0936*	*5.1*	*0.38*	*0.001041891*	*525±55*	*495±23*	*541±36*	*710±47*	*501±50*	*593±48*	*No description*

1) Genes in the upper panel of the table show rice homologues exhibiting conserved synteny with the region containing *Rphq11*.

2) Text in italics indicates that the direction of the correlation between eQTL and rust resistance is inverse (*i.e.* lower abundance, increased resistance and *vice versa*).

3) Asterisks show significantly different transcript abundance between the two parents.

4) Bold text represents the most promising candidates for *Rphq11*.

5) Transcript levels presented as average of the four replicates plus/minus standard error of mean.

We then examined the abundance of transcripts measured by these 54 probes for differential expression between *St* and *Mx* infected with *P. hordei*. Sixteen (marked with asterisks in Table 2) showed significant differential expression (fold change>2, FDR<0.05) between the two parents. Nine of these had rice homologues at a conserved syntenic position. We also compared the sign of the correlation coefficients between transcript abundance and resistance score ratios of the sample pairs, with the manner of regulation (up- or down-regulation in response to *Ph*-infection). Since the resistance allele is contributed by *St* for the locus of *Rphq11*, genes with positive correlations would reflect up-regulation in response to *Ph*-infection irrespective of statistical significance, and their transcripts should be more abundant in *St* than in *Mx* (and *vice versa* for genes with negative correlations). Twenty-two probes fit these criteria, whereas 32 showed an inconsistency between sign of correlations and manner of regulation (*i.e.* positive correlations associated with down-regulation, or *vice versa*). The genes represented by these 32 probes, from an eQTL analysis strategy, were therefore not considered candidates for *Rph11*. Six genes (bold, Table 2) fulfilled all the necessary characteristics of a rust resistance candidate eQTL (gene) for *Rphq11*.

## Discussion

eQTL analysis is potentially a powerful approach for the identification of genes underlying particular biological phenotypes [7], [8]. For the approach to be applicable to a specific trait, variation in the observed and measured phenotype of the trait is required to be the biological manifestation of variation in the expression of causal gene(s). In this study, to be detected directly by eQTL analysis, the causal genes responsible for partial resistance to *Puccinia hordei* would have to fulfill the following criteria. Firstly, transcript abundance in inoculated leaves would correlate positively or negatively with partial resistance. Secondly, both the causal gene and its eQTL would co-localize with pQTL, which means it is regulated in *cis-*. Thirdly, the causal gene would exhibit differential transcript abundance between two parental lines (either in non-inoculated or inoculated tissue). Only genes fulfilling each of these criteria would potentially be candidates for partial resistance. The eQTL strategy would not be valid in cases where the causal polymorphisms for a trait fail to change transcript levels [47]. For example, the eQTL approach would have failed to identify the recently cloned wheat gene *Lr34*, which confers durable resistance to multiple diseases, including leaf rust, stripe rust and powdery mildew [48]. *Lr34* encodes an ABC transporter with resistant and susceptible alleles having no polymorphism within 2kb 5′ of the gene, and only three polymorphisms in the coding region that are proposed to affect protein structure and substrate specificity. No expression differences are observed between resistant and susceptible lines and expression of *Lr34* does not depend on the presence of pathogens. Currently, we do not know whether partial resistance of barley to leaf rust has any connection with transcript abundance. However, in a species with a large and unsequenced genome such as barley, eQTL analysis offers an opportunity to identify genes that are closely linked to pQTL and that can be linked directly to fully sequenced model genomes. Differentially expressed positional candidates merit further investigation. Moreover, genome-wide eQTL analysis also provides a valuable dataset that can be used to investigate other traits assessed in the same population, even if they are not explicitly related to the tissue sampled for analysis.

Based on a microscopic assessment of the development of leaf rust infection over time in the barley cultivars Steptoe and Morex, we selected 18 hpi as an appropriate sampling time for a genetical genomics experiment that aimed to identify genes involved in partial resistance to leaf rust. This timepoint corresponds to the stage when plant cell walls are being penetrated and haustoria formation is being initiated and has previously been revealed to be crucial in barley accessions with partial resistance to *P. hordei* [44]. Caldo and co-workers [23] performed a time course analysis of interactions between barley and powdery mildew (*Blumeria graminis* f. sp. *hordei*), and found that expression profiles over the first 16 hpi were similar between incompatible and compatible reactions but diverged after this timepoint. This timing corresponds with the well-established kinetics of haustorium formation by *B. graminis* f. sp. *hordei* [23]. At haustorium formation fungal effector molecules are presumably delivered into host cells to suppress defense-related transcriptional responses [23], [24], [49]. In this study, the intermediate partial resistance phenotype of both parental lines prevented such a comparison. However, as 18 hpi corresponded to the formation of the first haustoria by the pathogen, we judged that it would represent a good choice for assessing the divergence of transcript abundance between lines that exhibit varying levels of partial resistance in the population.

We used Agilent microarray technology to measure transcript abundance. The two-channel feature allows pairs of RNA samples to be co-hybridised onto a single array after labeling with different fluorophores, thus, greatly reducing the impact of technical variation. We also used a distant pair design [36] which optimized the use of genetic diversity among individuals within the mapping population. In assembling the sample-pair matrix, we gave extra weight to markers linked to previously identified pQTL for partial resistance to leaf rust [4]. This increased the statistical power for detecting eQTL at these regions by maximizing the number of informative pair comparisons (Figure 2). Throughout the analysis we used normalized transcript abundance ratios of co-hybridised samples recorded on the same spot rather than their absolute signal intensity, which reduced the bias derived from spot and array effects [36]. We also used the same design in the experiment for tissue sampling by growing paired samples in the same trays which saved using checks in each tray. These combined approaches allowed us to generate a very robust dataset that was suitable for genetic investigation. It should be noted that the custom Agilent array was developed from the 22K Barley1 Affymetrix GeneChip [19] which has only partial coverage of the barley genome. Therefore potentially interesting genes may not present on the array and thus could have been missed out in the study.

We identified over 1100 genes that were differentially expressed in response to *Ph*-challenge in either of the parental lines. GO enrichment analysis identified over-representation of many *Ph*-responsive genes in the GO categories ‘response to stimulus’, ‘cell wall organization’, ‘metabolic process’ and one or more subcategories. These categories comprise many genes known to be involved in defense responses including defense-related transcription factors, genes involved in signal perception and transduction, hormone, phenylpropanoid pathway, and oxidative burst (Figure S2). Their patterns of regulation in response to *Ph*-infection are mostly in agreement with findings observed in other plant-pathosystems, such as up-regulation of genes coding for WRKY transcription factors, PR proteins and PALs, and down-regulation of genes involved in auxin signaling and light harvesting [50]–[54]. In a few cases, we did find contradictory patterns of regulation for genes annotated with the similar functions. For example, three PR genes were unexpectedly down-regulated. While we have no explanation for this latter observation, overall the *Ph*-responsive genes identified fit well into the generalized group of ‘host response to pathogen infection’ genes observed across different host-pathogen interactions [55], [56]. This suggests that the 18 hpi is representative of barley response to early *Ph*-infection, and appropriately chosen as the sampling timepoint for our genetical genomics experiment.

The relative density of eQTL across the genome showed that three regions were significantly enriched with eQTL for *Ph*-responsive genes (hotspots 1, 2 and 3 respectively) and could therefore represent the location of master regulators (*trans-*eQTL) that control the expression of networks of functionally related genes (Figure 6). However, as the observed eQTL density was calculated on genetic distance, high densities could result from genetically diverse and poorly recombining but gene rich regions such as the genetic centromeres. This however does not appear to be the case for the three regions with the highest eQTL density as they are located outside the centromeres and correspond to regions exhibiting relatively high recombination rates of 0.3–3.1 Mb/cM, 0.1 Mb/cM and 0.3 Mb/cM [57]. Furthermore, the three regions also had over ten times as many eQTL as compared to the genome average. The excessive number of eQTL in these regions may therefore have biological significance in this plant pathogen interaction. Gene ontology enrichment analyses revealed that eQTL hotspots 1 and 2 comprise genes forming conspicuous functional categories related to ‘response to stimulus’ and ‘localization’ respectively (*p*<0.05). These genes may therefore be components of a gene network or pathway controlled by a common upstream master regulator or *trans-*eQTL. Kliebenstein *et al.* [8] analyzed network eQTL for 20 well-studied gene networks using the averaged expression value of member genes as a measurable trait and found that network eQTL were located at same the regions as eQTL hotspots. We therefore speculate that hotspots 1 and 2 represent the location of underlying network or *trans-*eQTL that regulate expression of generalized defense responsive genes. In contrast, gene ontology enrichment analysis revealed no obvious biological process for genes whose eQTL were located at hotspot 3.

A master regulator (*trans-*eQTL) at an eQTL hotspot may function as the causal factor for a complex trait through regulation of specific trait-relevant pathways [8]. In our study however, none of the three eQTL hotspots co-located with any of the pQTL for rust resistance. This is not completely unexpected. The infection process on all lines, irrespective of their level of partial resistance, results in the differential regulation of many genes when compared to the mock inoculated treatment, and indicates that the pathogen directly influences the transcriptional response of numerous plant genes during the early phases of the interaction. This overall general response may be so strong that in a simple comparison (*e.g.* between resistant and susceptible lines) it would mask the differentially expressed genes that are actually responsible for the resistance phenotype. Genetic analysis can separate out these general effects from those responsible for the phenotype as eQTL should by necessity co-locate with pQTL. As the threshold we adopted for detection of *Ph*-responsive genes was stringent (fold change>2, FDR<0.05), it is likely that we would mostly detect highly differentially regulated genes involved in general defense responses. Individual components of the general defense response most often have incremental, rather than determinative, roles in the outcome of an interaction with a pathogen [58]. The observation that none of the eQTL hotspots overlapped with pQTL suggests that genes responsible for natural variation in partial resistance to *Ph* in this population are not *trans-*eQTL that control general defense responses. This conclusion is supported by the fact that many attempts to identify genes for disease resistance have ended up with those involved in signal transduction pathways [59], [60] or physiological or cellular functions [48], [61] rather than defense genes *per se* [62], [63].

eQTL were distributed across the barley genetic map and varied in magnitude and significance. Over 2500 genes had eQTL with *LOD*>10. We discounted the possibility that sequence polymorphisms between the probe and target were the cause of the observed high-*LOD* eQTL. While sequence polymorphisms have been shown to influence the efficiency of hybridisation between probe and target on 25-mer oligo Affymetrix arrays, generating Single Feature Polymorphisms (SFPs) [64], [45], the hybridisation dynamics of 60-mer oligos is relatively insensitive to SNPs [65], [66]. Therefore, we believe high *LOD* scores reflect extreme transcription level polymorphisms caused by variation in *cis*-acting elements. In eQTL studies with sequenced species like Arabidopsis, *cis*- and *tran*- eQTL can be determined by positional comparison of eQTL with corresponding gene. For unsequenced species such as barley, determining *cis*- or *trans*-eQTL is not so straightforward and is a limitation of our analysis. However, setting a threshold *LOD* score for declaring *cis*-eQTL is both arbitrary and experiment dependent. We only found for TDM-mapped genes some exceptions (7%) to the rule that LOD>10 eQTLs are located within 10 cM from the location of the corresponding genes. TDMs are based on transcript abundance differences and as 5% of TDMs may represent duplicate genes [30] this discrepancy is likely to be of true biological origin reflecting, for example, gene duplication or homologous transcripts from paralogous loci that are differentially expressed between tissues (*i.e.* infected leaf *vs.* germinated embryo). We therefore considered *LOD*>10 as a reasonable threshold for predicting the genetic map position of genes underlying *cis-*eQTL for the size and type of population we used in this study. Several other eQTL studies have shown that high *LOD* eQTL mainly reflect differentially *cis-*regulated allelic transcripts while *trans*-eQTL exhibit a less significant genetic effect [67], [46], [47], [28]. It is noteworthy that both Potokina *et al.* [30], [68] and the work we describe here used the same *St/Mx* population but different biological materials (germinating embryos compared to *Ph*-infected leaves) and different microarray platforms (Affymetrix *vs.* Agilent). That 93% of the common TDM's and *LOD*>10 eQTL mapped to the same genetic positions suggests that in different biological tissues, observed allelic transcript level differences tend to be conserved. Potokina *et al.* [68] investigated the phenomenon of limited pleiotropy in the *St/Mx* population using a highly selected set of 2081genes that showed the highest *LOD* scores for eQTL in two different tissue samples (germinating embryo and young leaf). They observed that for approximately half (1083) of these genes, *cis-*regulatory variation was consistent among both tissues, and for the remaining 998 genes *cis-*regulation was tissue-specific (*e.g.* a gene was only expressed in one tissue). Thirty-four genes were identified where the direction of the *cis-*effect was reversed in the different tissues. In *C. elegans*, Li *et al.* [69] discovered that 8% *cis*-eQTL showed eQTL-by-environment interaction as opposed to 59% for *trans*-eQTL. One obvious outcome of these observations is that for *cis*-regulated genes, eQTL datasets obtained from one particular experiment (*e.g.* set of conditions, tissue or treatment) may be of considerable value for transcript abundance-based candidate gene identification for other traits that segregate in the same genetic material but are not necessarily measured in the same tissues/times. Supporting this idea is the recent report by Druka *et al.* [31] who demonstrated that *Rpg1*, the causal gene for barley stem rust resistance in the *St/Mx* population, could be successfully predicted based on transcript abundance data generated from uninfected germinating embryos.

Converting the resistance scores into ratios for each distant pair prior to performing eQTL and correlation analysis proved to be a highly robust approach. It allowed us to reproduce the identification of four previously discovered pQTL [4] and the *Rphq19* locus reported in a different population. It also allowed us to identify 95 eQTL co-located with at least one of the five pQTL from 89 genes that were correlated in transcript abundance with rust resistance. Notably, a subset of 54 eQTL co-located with *Rphq11*, the pQTL of the largest resistance effect. eQTL for the 22 genes that were most strongly correlated with rust resistance (|*r*|≥0.47, *p*<10^−4^) exclusively mapped to *Rphq11*. As the biological samples used for eQTL analysis were not the same plants used for disease evaluation we may have reduced the power of the correlation analysis which would result in a reduction of the number of significantly correlated genes The 128 genes we identified may therefore be an underestimate. The observation that so many genes are correlated with rust resistance and their eQTL co-localize with pQTL is not entirely unexpected. For genes located within the pQTL regions, this correlation is almost certainly the result of their physical linkage to the causal gene and their regulation in *cis*-. Subsequent analysis of putative function and genetic distance from the pQTL peak can exclude many of these eQTL as candidate genes. For genes located outside the pQTL regions, their correlation with rust resistance may either represent chance events or linked biological functions that operate downstream of the causal gene(s). Notably, we observed that many eQTL (from 25 out of 35 genes) that did not coincide with pQTL were located at one of the three eQTL hotspots (Table S5). This suggests that wider transcriptional reprogramming in response to *Ph*-infection is under the control of ‘general response’ *trans-*eQTL located at the observed eQTL hotspots, an explanation that would thus account for the correlations between the transcript abundance of these genes and rust resistance.

Conservation of synteny with rice allowed us to predict the physical location of 31 of the 54 genes underlying eQTL at *Rphq11*. The high *LOD* (>10) eQTL for 25 of these also strongly suggested that they were physically located close to *Rphq11* (Table 2). If a positional candidate is to be considered the causal gene underlying a given phenotype directly as the result of eQTL analysis then it must be regulated in *cis-*. While high *LOD* eQTL usually suggests *cis*-regulation [46], [47], [28], due to the lack of information on the precise physical location of genes in barley, it is not possible to definitively resolve *cis*- from *trans*-eQTL on the basis of *LOD* scores alone. However, *cis*-regulated genes should exhibit significantly different transcript abundances in the parental lines. Of the 31 genes located at *Rphq11*, nine showed such differences between the two parents (FC>2, FDR<0.05) but these only showed subtle changes in transcript abundance after *Ph*-infection as compared to mock controls and were not classified *Ph*-responsive genes. Of course there is no requirement for the causal gene to be responsive to *Ph*-infection. We know that resistance conferred by *Rphq11* is mediated by the *St* allele [4]. We have no evidence to differentiate whether this is due to an increase or decrease in the abundance of transcript from the causal gene or not (it could be a protein functional mutation). However, if resistance at this locus is ultimately attributed to variation in transcript abundance then we may logically expect that a positive correlation would be associated with increased transcript abundance and a negative correlation with decreased transcript abundance. Applying this criterion excludes three, leaving six genes as the promising candidates (Table 2). Of these six, ‘unigene2453’ encoding a phospholipid hydroperoxide glutathione peroxidase (PHGPx) is perhaps the strongest candidate. Tomato *Le*PHGPx has been shown to function as a cyto-protector, preventing *BAX-*, hydrogen peroxide-, and heat stress-induced cell death. Moreover, stable expression of LePHGPx in tobacco conferred protection against the fungal phytopathogen *Botrytis cinerea* [70]. As a result, we are currently testing the hypothesis that ‘unigene2453’ is the causal gene underlying *Rphq11*.

## Materials and Methods

### Plant Growth

Barley cultivars Steptoe (*St*) and Morex (*Mx*) and 144 doubled haploid (DH) lines from their segregating progeny were used throughout. Steptoe is a high yielding broadly adapted six-row barley cultivar and Morex is the North American six-row malting quality standard. Distribution of resistance levels across the progeny exhibited a typical normal distribution with ‘relative latency period’ (RLP50S) in hours ranging from 100 to 123. Both parents had similar levels of resistance with RLP50S of 118 for *Mx* and 119 for *St* (referred to [4] for details). Four biological replicates with both pathogen-infected treatment and mock-inoculated controls were set for parental lines, while a single replicate with pathogen-infected treatment was used for the progeny. The DH lines were sorted into pairs based on a distant pair design [36], as described in the next section. Paired lines with 10 seedlings each were grown together in trays (37×39 cm) in two rows 30 cm apart. Each tray contained three pairs. All seedlings were grown in a glasshouse compartment. The plant growth conditions were similar as described previously by Qi *et al.* [35] with temperature of 24°C day and 18°C night, light length of 16 hours and relative humidity of 60%.

### Distant Pair Design for Sampling and Microarray Analysis

We used a distant pair design, as proposed for two-colour microarrays by Fu and Jansen [36] to improve the efficiency of eQTL studies. The design uses genetic marker information to identify pairs of individuals with maximum dissimilarity across the mapping population. In calculating the optimal pairing, extra weight was given to markers in regions already known to affect the trait of interest. Briefly, the distant pair analysis was based on 466 SNP markers from Rostoks *et al.* [32]. From these markers a framework set of 119 SNP markers was chosen as having no missing data and even spacing across the genetic map. In the confidence intervals where the four pQTL for partial resistance to leaf rust had been previously located [4] framework markers were given a weight of ten, while markers in other regions were given a weight one. Following Fu and Jansen [36], a ‘simulated annealing’ algorithm [37] was used to find an optimal pairing matrix.

### Pathogen Inoculation

Barley leaf rust isolate *P. hordei* 1.2.1, to which no *R* genes are effective in either *St* or *Mx*, was used for inoculation of nine-day old seedlings with fully developed first leaves. Leaves were laid horizontal and gently fixed over the soil prior to inoculation. Inoculation was performed as described by Qi *et al.* [35] with minor modifications. Briefly, per plant tray, 8 mg of urediospores of *P. hordei* isolate 1.2.1 amounting to a spore deposition of about 500 spores per cm^2^, plus 32 mg of *Lycopodium* spores (added as a carrier) were thoroughly mixed by vortexing and applied to the adaxial sides of the seedling leaves using a settling tower inoculation facility. Mock inoculation of parental lines was carried out using 40 mg of *Lycopodium* spores only. All trays were transferred to a dark chamber at 18°C, 100% humidity for 10 hours, before being placed in the glasshouse for infection development.

### Microscopic Investigation of Fungal Development

To identify an optimal timing of sampling for the subsequent eQTL experiment, an exploratory experiment containing only *St* and *Mx* was performed. Progress of pathogen development was investigated using epi-fluorescence microscopy, according to Rohringer *et al.* [38]. Segments (1–3 cm) of the infected first leaves were excised from seedlings at 10, 18, 24, 34, 42, and 48 hours post inoculation (hpi) and collected into glass tubes containing a lactophenol-ethanol (1∶2 v/v) solution and placed in a boiling water bath for 1.5 min. The solution was replaced by clean lactophenol-ethanol and left at room temperature overnight. Leaf segments were washed, first with 50% ethanol for 30 min then with 0.05N NaOH for 30 min, and finally rinsed with water. Leaf segments were treated with 0.1 M Tris-HCl (pH8.5) by soaking for 30 min prior to staining in a solution of 0.1% Uvitex 2B (Ciba-Geigy) for 5 min. Samples were thoroughly rinsed with water, soaked in 25% glycerol for 30 min and mounted onto glass slides. Pathogen development stages were examined at different time points under an epi-fluorescence microscope and 18 hpi was identified as the critical time-point when direct physical interaction was becoming established through penetration of the host cell walls.

### Leaf Sampling and RNA Isolation

At 18 hpi, pathogen-inoculated leaves from each of the 144 DH lines were collected separately into Falcon tubes and immediately flash frozen in liquid nitrogen and stored at −80°C until use. One or two seedlings of each line were left uncut to ensure that the expected disease symptoms developed 5 days after inoculation, confirming that the inoculations were successful and samples were suitable for analysis.

Approximately 0.5 g of frozen leaf tissue was ground to a powder in liquid nitrogen. RNA was isolated with 5 ml TriZol extraction buffer (Invitrogen) as recommended by the supplier. The extracted RNA solution was immediately treated with RNase inhibitor SUPERase-In (Ambion) followed by digestion with DNaseI (Ambion) according to the manufacturer's instructions. RNA samples were purified using RNeasy Mini Kits (Qiagen) and quantified using a NanoDrop ND-100 spectrophotometer (NanoDrop Technologies). The yield was typically 200 µg of total RNA/g of wet tissue. RNA Concentrations were equilibrated to 500 ng/µl and RNA quality was checked on an Agilent Bioanalyzer 2100 electrophoresis system (Agilent Technologies) and stored at −80°C until use.

### Barley Custom Agilent Microarray

A barley custom array was designed in-house using eArray (Agilent http://www.chem.agilent.com; design number 015862). The array contains a total of 15744 60-mer oligonucleotide features including control probes and orientation markers. Of these, 15208 barley probes are derived from unigenes of assembly #25 used to design probesets for the 22K Barley1 Affymetrix GeneChip [19]. Each unigene was represented by a single 60-mer ologonucleotide probe. The unigenes included were chosen from the 22K Barley1 Affymetrix Gene Chip by eliminating redundant or poorly performing probe-sets identified in previous experiments. The probe identifiers and their corresponding cDNA sequences can be found at ArrayExpress (http://www.ebi.ac.uk/microarray-as/ae/; accession # A-MEXP-1471). The arrays were fabricated by Agilent in 8×15k format (http://www.chem.agilent.com).

### Microarray Processing

Total RNA was labeled by indirect incorporation of fluorescent dyes following cDNA synthesis. Reverse transcription was performed using 5 µg of total RNA in a 45 µl reaction containing 50 ng/µl oligo d(T)18, 0.5 mM each dATP, dCTP, dGTP, 0.2 mM dTTP, 0.3 mM aa-dUTP, 10 mM DTT, and 400 U Superscript II (Invitrogen) in 1× reaction buffer. Primers and RNA were initially heated to 70°C for 10 min followed by cooling on ice, and the entire reaction incubated for 16 h at 42°C. To denature the remaining RNA, 15 µl of 1 M NaOH and 15 µl of 0.5 M EDTA (pH 8.0) were added and incubated for 10 min at 65°C. The reaction was neutralized with 15 µl of 1 M HCl. Purification of cDNA was performed using MinElute columns as recommended (Qiagen), substituting phosphate wash buffer (4.75 mM K_2_HPO_4_, 0.25 mM KH_2_PO_4_, 84% EtOH) for PB and phosphate elution buffer (3.8 mM K_2_HPO_4_, 0.2 mM KH_2_PO_4_) for EB. Cy-dye esters were added to 10 µl of cDNA in a total volume of 13 µl, containing 150 mM sodium carbonate and 1 µl of the appropriate Cy-dye (GE Healthcare) suspended in DMSO (1/10 supplied aliquot), and incubated for 1 h at room temperature in the dark. To the labeled cDNA, 750 mM hydroxylamine hydrochloride was added and incubated for a further 30 min in the dark. Labeled targets for each array were combined and diluted with 24 µl sterile water and 500 µl of PB buffer (Qiagen) prior to MiniElute purification and elution with 2×10 µl of EB buffer. Labeling efficiency was estimated spectrophotometrically. Samples with dye incorporation of >2–3 pmol/µl and cDNA concentration of 40–60 ng/µl were used for hybridisations.

### Sample Hybridisation and Array Washing

Hybridisation and washing were conducted according to the manufacturer's protocols (Agilent, Two-Color Microarray-Based Transcript Abundance Analysis, version 5.5). Briefly, 20 µl labeled samples were added to 5 µl 10× blocking agent (Agilent 5188–5242) and heat denatured at 98°C for 3 min then cooled to room temperature. 2× GE Hybridisation buffer HI-RPM (25 µl) was added and mixed prior to hybridisation at 65°C for 17 hours at 10 rpm. Array slides were dismantled in Wash 1 buffer (Agilent, 5188–5327) and washed in Wash 1 buffer for 1 min, then washed in Wash 2 solution (Agilent, 5188–5327) for 1 min, and centrifuged dry. Hybridised slides were scanned using an Agilent G2505B scanner at resolution of 5 µM at 532 nm (Cy3) and 633 nm (Cy5) wavelengths with extended dynamic range (laser settings at 100% and 10%).

### Sample Layout of Parental Lines for Co-Hybridisation on Microarray

For *Ph*-responsive gene identification, RNA samples of *Ph*-infected parental lines were co-hybridised with their corresponding mock controls using 4 arrays for 4 biological replicates of each parent. Dye-swap duplicates were performed with two replicates to obtain dye balance (array slide 1 in Table S1). For identification of differentially expressed genes between the two parents, RNA samples of *Ph*-infected *St* and *Mx* were co-hybridised on the same arrays with four biological replicates of which two were applied with dye-swap (array slide 2 in Table S1).

### Deposition of Microarray Data

The raw microarray data and relevant experimental metadata, which are MIAME (Minimum Information About a Microarray Experiment) compliant, were stored in a local instance of the BASE laboratory information management system (http://base.thep.lu.se/), and from there were submitted to the ArrayExpress microarray data archive (http://www.ebi.ac.uk/microarray-as/ae/) at the European Bioinformatics Institute (accession numbers: E-TABM-645 for individual DH lines of the St/Mx population and E-TABM-747 for parental lines), by means of a custom-written plugin for BASE.

### Data Extraction, Normalisation and Significance Criteria for Differential Expression

Microarray images were imported into Agilent Feature Extraction (FE) (v.9.5.3) software and aligned with the appropriate array grid template file (015862_D_F_20070525). Intensity data and QC metrics were extracted using the manufacturer-recommended FE protocol (GE2-v5_95_Feb07). Entire FE datasets for each array were imported into GeneSpring (v.7.3) software for further analysis. Data from each array were Lowess (LOcally WEighted polynomial regreSSion) normalized to minimize differences in dye incorporation efficiency in a two-channel microarray platform [39]. For the replicated experiment with parental lines, dye swap was taken into account prior to Lowess normalization. Differentially expressed genes were first selected on fold change>2 followed by a *t*-test on log-transformed normalised ratio data by setting the false discovery rate (FDR) to 0.05.

### Gene Function Enrichment Analysis

After a list of *Ph*-responsive genes was obtained, the Gene Ontology Enrichment Analysis Toolkit (http://omicslab.genetics.ac.cn/GOEAST) [41] was used with the default settings (hypergeometric test with multi-test adjustment of Benjamini and Yekutieli [42] at FDR of 0.1) to analyze functional enrichment focusing on the functional category ‘Biological Processes’. Significantly enriched gene ontology (GO) categories containing at least 3 genes were used for presentation.

### Statistical Model for eQTL Analysis

eQTL analysis used the linear model proposed by [36]. This relates the log ratios of transcript abundance to the (SNP) markers on the linkage map (for each marker in turn). The model for transcript abundance at each marker can be expressed as
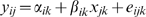
(1)where ‘*y_ij_*’ is the log ratio of transcript abundance of pair ‘*j*’ for gene ‘*i*’, and ‘*x_jk_*’ shows the marker allele information for the pair ‘*j*’ at marker ‘*k*’ with *x_jk_* = 1, and −1 for the pairs *St/Mx*, *Mx/St* respectively and *x_jk_* = 0 for the pairs *St/St* or *Mx/Mx*. The regression coefficient *β_ik_* shows the effect of the allele difference at marker ‘*k*’ on gene ‘*i*’, the intercept *α_ik_* should be close to zero unless there is dye bias and *e_ijk_* is the residual error.

The log-normalised ratios of transcript levels of the paired lines from each of the 15208 genes were employed, as transcript abundance phenotypic data in the linear model and tested for association with each of the 466 SNP markers across the 7 chromosomes independently using a threshold of *p*<0.001 to declare significant eQTL. When multiple markers on the same chromosome detected a significant association, only the most significant marker was selected to represent the eQTL on that chromosome. The residuals were then tested for further eQTL. In this second round test, a regression of the log ratio on all of the markers that indicated the most significant association on each chromosome was performed, and the residuals estimated. The residuals were then reanalyzed using equation (1) to test for further eQTL, either on the same or different chromosomes, in the next round. Markers with the highest logarithm of odds ratio (*LOD*) score, the corresponding *p*-value, the variation explained by the eQTL (*R* ^2^) and the eQTL additive effect were stored as output of the analysis.

The rust resistance trait, ‘relative latency period (RLP50S)’, which had been used previously for the discovery of the four pQTL *Rphq*8, *Rphq*11, *Rphq*14 and *Rphq*15 [4], was reanalysed using the QTL model of equation (1). The Pearson correlation coefficient was calculated between the RLP50S ratio and the normalised ratio of transcript abundance for each of the 15208 genes.

## Supporting Information

Figure S1 Venn diagram showing number of Ph-responsive genes and genes differentially expressed after Ph-infection. Red and green circle represent Ph-responsive genes identified from St and Mx respectively that are significantly (fold change>2 and FDR<0.05) altered after Ph-infection compared to mock controls. Blue circle represents significant (fold change>2 and FDR<0.05) differently expressed genes between the parental lines after Ph-infection. Venn diagram showing number of Ph-responsive genes and genes differentially expressed after Ph-infection. Red and green circle represent Ph-responsive genes identified from St and Mx respectively that are significantly (fold change>2 and FDR<0.05) altered after Ph-infection compared to mock controls. Blue circle represents significant (fold change>2 and FDR<0.05) differently expressed genes between the parental lines after Ph-infection.
(0.42 MB EPS)

Figure S2 Functional classification of the 1154 Ph-responsive genes. Number of up (+) or down (−) regulated genes are shown in the table attached on the right side (see Table S1 for details).
(0.70 MB EPS)

Table S1 Microarrays performed on parental lines for identification of Ph-responsive genes (array slide 1) and differentially expressed genes (array slide 2).
(0.04 MB DOC)

Table S2 Gene ontology enrichment analysis of Ph-responsive genes and genes with eQTL at hotspots 1 and 2.
(0.05 MB DOC)

Table S3 Transcript abundance of Steptoe and Morex infected by *Puccinia hordei* compared with mock control.
(0.40 MB DOC)

Table S4 Differentially expressed genes in Ph-infected seedlings between Steptoe and Morex.
(0.22 MB XLS)

Table S5 eQTL for Ph-responsive genes.
(0.34 MB XLS)

Table S6 eQTL for the 128 resistance-correlated genes and positional overlapping with pQTL and Ph-responsive eQTL hotspots.
(0.07 MB XLS)
