# Supplementary material for: An eQTL Analysis of Partial Resistance to Puccinia hordei in Barley
Source: PLoS One. 2010 Jan 6;5(1):e8598. doi: 10.1371/journal.pone.0008598 (PMC2798965; doi:10.1371/journal.pone.0008598)
Supplement: Table S1 — Microarrays performed on parental lines for identification of Ph-responsive genes (array slide 1) and differentially expressed genes (array slide 2). (0.04 MB DOC) [file pone.0008598.s003.doc]

**Table S1. Microarrays performed on parental lines for identification of *Ph***-responsive genes (array slide 1) and differentially expressed genes (array slide 2)

| **Array slide** | **Array name** | **Replicate** |  | **Sample pairs** |
| --- | --- | --- | --- | --- |
| 1 | 1-1 | 1 |  | *St*-M-C3 / *St*-P-C5 |
| 1 | 1-2 | 1 |  | *Mx*-M-C3 / *Mx*-P-C5 |
| 1 | 1-3 | 2 |  | *St*-M-C5 / *St*-P-C3 |
| 1 | 1-4 | 2 |  | *Mx*-M-C5 / *Mx*-P-C3 |
| 1 | 2-1 | 3 |  | *St*-M-C3 / *St*-P-C5 |
| 1 | 2-2 | 3 |  | *Mx*-M-C3 / *Mx*-P-C5 |
| 1 | 2-3 | 4 |  | *St*-M-C5 / *St*-P-C3 |
| 1 | 2-4 | 4 |  | *Mx*-M-C5 / *Mx*-P-C3 |
| 2 | 1-1 | 1 |  | *St*-P-C3 / *Mx*-P-C5 |
| 2 | 1-2 | 2 |  | *St*-P-C5 / *Mx*-P-C3 |
| 2 | 1-3 | 3 |  | *St*-P-C3 / *Mx*-P-C5 |
| 2 | 1-4 | 4 |  | *St*-P-C5 / *Mx*-P-C3 |

Note: *St*=Steptoe, *Mx*=Morex, M=mock control, P=*Ph*-infected, C3=labeling dye Cy3, and C5=labeling dye Cy5
