# Supplementary material for: An eQTL Analysis of Partial Resistance to Puccinia hordei in Barley
Source: PLoS One. 2010 Jan 6;5(1):e8598. doi: 10.1371/journal.pone.0008598 (PMC2798965; doi:10.1371/journal.pone.0008598)
Supplement: Table S2 — Gene ontology enrichment analysis of Ph-responsive genes and genes with eQTL at hotspots 1 and 2. (0.05 MB DOC) [file pone.0008598.s004.doc]

**Table S2. Gene ontology enrichment analysis of *Ph*-responsive genes and genes with eQTL at hotspots 1 and 2**

| GO ID | GO term | *Ph*-responsive | Hotspot-1 | Hotspot-2 |
| --- | --- | --- | --- | --- |
| GO:0050896 | response to stimulus | 0.0135 | 0.0018 |  |
| GO:0009607 | response to biotic stilumus | 2.68E-06 | 6.15E-07 |  |
| GO:0006950 | response to stress | 0.0071 | 0.0013 | 0.0445 |
| GO:0006952 | defense response | 0.0490 | 0.0009 |  |
|  |  |  |  |  |
| GO:0009987 | cellular process |  |  | 0.0019 |
| GO:0016043 | cellular component organization |  |  | 0.0159 |
| GO:0007047 | cell wall organization | 1.96E-05 |  | 0.0070 |
|  |  |  |  |  |
| GO:0051179 | localization |  |  | 0.0435 |
| GO:0006810 | transport |  |  | 0.0435 |
| GO:0033036 | macromolelule localization |  |  | 0.0406 |
| GO:0015031 | protein transport |  |  | 0.0406 |
|  |  |  |  |  |
| GO:0008152 | metabolic process | 0.0002 | 0.0015 | 0.0018 |
| GO:0055114 | oxidation reduction |  | 0.0033 | 0.0288 |
| GO:0044237 | cellular metabolic process | 0.0146 | 0.0112 | 0.0012 |
| GO:0006730 | one-carbon compound metabolic process |  |  | 0.0001 |
| GO:0044238 | primary metabolic process | 0.0161 | 0.0197 |  |
| GO:0005975 | carbohydrate metabolic process | 0.0071 |  |  |
| GO:0005976 | polysaccharide metabolic process | 0.0017 |  |  |
| GO:0006073 | cellular glucan metabolic process | 0.0161 |  |  |
| GO:0009056 | catabolic process | 0.0113 | 0.0164 |  |
| GO:0019439 | aromatic compound catabolic process | 0.0113 |  |  |
| GO:0006559 | L-phenylalanine catabolic process | 0.0113 |  |  |
| GO:0043170 | macromolecule metabolic process |  | 0.0375 |  |
| GO:0044267 | cellular protein metabolic process |  | 0.0275 |  |
| GO:0006457 | protein folding | 0.0014 | 0.0001 |  |
