# Supplementary material for: An eQTL Analysis of Partial Resistance to Puccinia hordei in Barley
Source: PLoS One. 2010 Jan 6;5(1):e8598. doi: 10.1371/journal.pone.0008598 (PMC2798965; doi:10.1371/journal.pone.0008598)
Supplement: Supplementary file 3 [file pone.0008598.s005.doc]

## Sheet: Sheet1

Table S3. Transcript abundance of Steptoe and Morex infected by Puccinia hordei compared with mock control
	Steptoe					Morex
Hv25-Unigene ID	Ratio (Ph/Mock)	t-test P-value	Log	Ph-infected	Mock control 	Ratio (Ph/Mock)	t-test P-value	Log	Ph-infected	Mock control 	rice homologue	annotated function	functional category
gb_UNIGENE7517	2.242171	0.007403727	0.80744463	5782.5625	2624.7017	1.8663439	0.00013001157	0.62398136	5577.1475	2995.0518	LOC_Os01g14440.1         	OsWRKY1v2 - Superfamily of rice TFs having WRKY and zinc finger domains	transcription factor
gb_UNIGENE23697	3.6516314	0.0058650095	1.295174	5927.73	1592.0154	3.2648551	0.0058360514	1.1832154	15297.9	4595.5977	LOC_Os01g61080.1         	OsWRKY24 - Superfamily of rice TFs having WRKY and zinc finger domains	transcription factor
gb_UNIGENE4387	9.129041	0.0060362536	2.2114606	445.03424	50.353256	8.402462	0.0025925243	2.1285248	836.27576	106.65663	LOC_Os06g44010.1         	OsWRKY28 - Superfamily of rice TFs having WRKY and zinc finger domains	transcription factor
gb_UNIGENE15957	3.7365925	0.016091654	1.3181741	966.705	232.32935	4.1866083	0.003905606	1.431891	201.0785	48.796837	LOC_Os05g25770.1         	OsWRKY45 - Superfamily of rice TFs having WRKY and zinc finger domains	transcription factor
gb_UNIGENE21110	2.8306844	0.010378287	1.0405185	738.7153	257.86124	2.35449	0.02207299	0.8563242	684.2358	325.28125	LOC_Os08g29660.1         	OsWRKY69 - Superfamily of rice TFs having WRKY and zinc finger domains	transcription factor
gb_UNIGENE10167	14.560221	0.00028033176	2.6782932	4410.9697	306.50943	12.195223	0.013911527	2.5010443	2756.9624	336.63513	LOC_Os05g46020.1         	OsWRKY7 - Superfamily of rice TFs having WRKY and zinc finger domains	transcription factor
gb_UNIGENE10168	20.121342	0.001905282	3.001781	3622.5825	180.12083	35.30167	0.00014225225	3.5639303	2405.1602	69.18106	LOC_Os05g46020.1         	OsWRKY7 - Superfamily of rice TFs having WRKY and zinc finger domains	transcription factor
gb_UNIGENE4386	4.0909514	0.0058996957	1.4087776	7592.0176	1820.107	3.875871	0.0040834723	1.3547704	4470.8174	1154.8147	LOC_Os02g08440.1         	OsWRKY71 - Superfamily of rice TFs having WRKY and zinc finger domains	transcription factor
gb_UNIGENE12005	3.312661	0.008251733	1.1977518	4352.258	1258.1184	3.1638637	0.0005076983	1.151794	3628.7324	1157.1562	LOC_Os09g16510.1         	OsWRKY74 - Superfamily of rice TFs having WRKY and zinc finger domains	transcription factor
gb_UNIGENE18462	7.219461	0.011257931	1.9767803	316.673	48.09081	6.237141	0.000117732474	1.830522	210.247	33.255367	LOC_Os01g40260.1         	OsWRKY77 - Superfamily of rice TFs having WRKY and zinc finger domains	transcription factor
gb_UNIGENE22244	0.35759416	0.0041242116	-1.0283566	54.695347	143.13287	0.4259287	0.0016181725	-0.8534833	173.29926	406.07916	LOC_Os05g32270.2         	AP2 domain transcription factor	transcription factor
gb_UNIGENE6636	4.647655	0.00070798106	1.5363628	3582.7825	772.7375	4.8319283	0.0002960186	1.5752456	3427.6775	699.1906	LOC_Os05g41780.1         	ethylene-responsive transcription factor 4	transcription factor
gb_UNIGENE2470	5.997803	0.007149163	1.7913933	1351.5425	239.13043	5.4570756	0.0004517391	1.696913	1249.6729	226.24785	LOC_Os04g52090.1         	ethylene-responsive transcription factor 4	transcription factor
gb_UNIGENE9431	3.7211754	0.0036403881	1.3140396	9568.357	2582.4656	2.5770235	0.00068752404	0.946635	6930.3623	2689.045	LOC_Os06g41770.1         	bZIP transcription factor protein	transcription factor
gb_UNIGENE21171	0.32812104	0.0146297375	-1.1143727	86.2809	249.73521	0.24288929	0.002640582	-1.4151496	55.787	226.49069	LOC_Os10g41230.1         	homeobox-leucine zipper protein HAT1	transcription factor
gb_UNIGENE10112	0.49671498	0.0014252554	-0.6997389	1310.314	2623.4507	0.6148343	0.0038883716	-0.48640248	1256.3425	2059.0757	LOC_Os03g08960.1         	homeodomain-leucine zipper transcription factor TaHDZipI-1	transcription factor
gb_UNIGENE13148	0.46544617	0.013188196	-0.7647588	604.56024	1337.1814	0.5993017	0.023251973	-0.51199013	539.02423	914.30194	LOC_Os09g35910.1         	homeodomain-leucine zipper transcription factor TaHDZipI-1	transcription factor
gb_UNIGENE17684	2.776204	0.001818273	1.0210845	6861.0874	2490.2847	2.5422223	0.0030310529	0.9330386	6141.8574	2383.7178	LOC_Os02g56250.1         	GATA zinc finger family protein	transcription factor
gb_UNIGENE5687	0.33181933	0.010565805	-1.1031647	2255.6626	6859.3677	0.5349977	0.0037089165	-0.6254928	3211.6726	6056.129	LOC_Os05g10670.1         	zinc finger C-x8-C-x5-C-x3-H type family protein	transcription factor
gb_UNIGENE20981	2.5314734	0.0005097903	0.92880154	81.8535	32.95251	3.581528	0.006870923	1.2757895	68.69737	21.64692	LOC_Os03g60570.1         	zinc finger DNA-binding protein	transcription factor
gb_UNIGENE6759	2.9009616	0.0006926379	1.0650423	34775.598	11842.395	2.517035	0.00039109995	0.92308164	26763.299	10587.709	LOC_Os05g03760.1         	zinc finger transcription factor-like protein	transcription factor
gb_UNIGENE21793	10.842061	0.0004473118	2.383433	157.24075	15.277884	10.740745	0.0023406795	2.3740444	116.656845	10	LOC_Os03g32230.1         	zinc-finger protein 1	transcription factor
gb_UNIGENE18088	4.5363917	0.0033022203	1.5121319	5945.515	1326.6924	4.1760674	0.004712702	1.4293699	4960.995	1195.3888	LOC_Os03g55540.1         	zinc-finger protein 1	transcription factor
gb_UNIGENE11225	4.323597	0.0034639577	1.4640877	2060.2676	464.00668	3.765517	0.0048028273	1.3258852	1247.6875	341.7288	LOC_Os03g08330.1         	ZIM motif family protein	transcription factor
gb_UNIGENE25131	3.8778973	0.010738624	1.355293	251.12476	69.97048	5.454833	0.0017652137	1.696502	192.96574	36.017612	LOC_Os04g32480.1         	ZIM motif family protein	transcription factor
gb_UNIGENE4815	1.304572	0.1897671	0.26587498	240.522	182.9894	2.113372	0.0062774406	0.7482848	206.4985	99.043076	LOC_Os10g25230.1         	ZIM motif family protein	transcription factor
gb_UNIGENE13355	3.028945	0.02241114	1.1082144	6697.9624	2128.8875	3.6816142	0.009030203	1.3033513	7648.6045	2012.6528	LOC_Os01g39330.1         	helix-loop-helix DNA-binding domain containing protein	transcription factor
gb_UNIGENE14337	0.44520962	0.03139664	-0.80921006	219.9605	497.773	0.3763035	0.017626408	-0.9773593	212.11826	561.33575	LOC_Os05g46610.1         	myb-related protein Hv33	transcription factor
gb_UNIGENE3362	2.194917	0.0024347275	0.78614426	3513.9001	1598.1326	1.9843651	0.018867634	0.68529904	2402.9502	1194.6746	LOC_Os01g60020.1         	NAC domain-containing protein 68	transcription factor
gb_UNIGENE11081	0.47776684	0.015324636	-0.73863244	2353.0942	4481.27	0.57545775	0.010621381	-0.5525894	2414.9702	4214.905	LOC_Os03g29970.1         	nuclear transcription factor Y subunit B-3	transcription factor
gb_UNIGENE3667	7.7911057	0.0032962887	2.0529828	39584.523	5070.8047	12.220603	0.00065835967	2.5031233	38324.95	3157.2478	LOC_Os01g59660.2         	transcription factor GAMYB	transcription factor
gb_UNIGENE23540	3.2909143	0.0021378512	1.1911654	3157.0176	964.91614	3.112909	0.02777685	1.1355577	145.75275	50.606644	LOC_Os01g47070.1         	acidic endochitinase precursor	PR protein
gb_UNIGENE2992	10.386199	0.00023486152	2.340478	74594.35	7350.99	15.778141	0.000011299412	2.7586255	64263.2	4056.3638	LOC_Os10g39680.1         	acidic endochitinase Q precursor	PR protein
gb_UNIGENE2990	12.840089	0.0006239621	2.5525723	18188.875	1401.4818	25.617773	0.00013628752	3.2432864	14991.851	586.6223	LOC_Os10g39680.1         	acidic endochitinase Q precursor	PR protein
gb_UNIGENE4173	4.7974772	0.0049700895	1.5680902	449.68173	102.88553	4.9245744	0.008001233	1.5942378	575.034	130.08797	LOC_Os05g33140.1         	basic endochitinase 1 precursor	PR protein
gb_UNIGENE7001	1.3175067	0.8497661	0.27574104	59.945534	16.680275	5.4447355	0.00049403275	1.6946492	218.207	39.28261	LOC_Os06g51060.1         	basic endochitinase 1 precursor	PR protein
gb_UNIGENE5023	3.2502759	0.0038081764	1.1787399	1045.3075	329.17462	2.4832094	0.011929183	0.9095518	236.37276	98.12433	LOC_Os10g28080.1         	chitinase 1 precursor	PR protein
gb_UNIGENE4326	6.370265	0.00024637466	1.851641	17102.725	2760.0898	5.7610383	0.0020137713	1.7511177	9430.223	1684.5955	LOC_Os04g41680.1         	endochitinase A precursor	PR protein
gb_UNIGENE4324	6.7971573	0.0003842105	1.9165045	8586.825	1310.8712	4.414933	0.0013464151	1.4849927	3552.9875	823.2475	LOC_Os04g41680.1         	endochitinase A precursor	PR protein
gb_UNIGENE14498	3.6169295	0.0036509628	1.2856255	1449.5975	394.77618	2.930333	0.005172827	1.075116	1061.889	365.2496	LOC_Os02g39330.1         	endochitinase PR4 precursor	PR protein
gb_UNIGENE13838	4.372554	0.0026360229	1.4753473	111.28262	24.930676	6.258503	0.0011239442	1.833941	153.3035	24.824883	LOC_Os09g23084.1         	endoglucanase 1 precursor	PR protein
gb_UNIGENE15553	8.965062	0.0062077204	2.193335	2554.7954	241.2956	8.809245	0.0003520897	2.1758018	2394.2847	264.47025	LOC_Os07g35520.1         	glucan endo-1,3-beta-glucosidase 3 precursor	PR protein
gb_UNIGENE13906	1.3870803	0.2160117	0.32720107	42.6963	30.857761	2.2436726	0.014069375	0.80811405	30.798	14.137555	LOC_Os01g14140.1         	glucan endo-1,3-beta-glucosidase 4 precursor	PR protein
gb_UNIGENE10415	0.6153122	0.20169778	-0.4856255	47.93562	73.83865	0.41701987	0.030791719	-0.8746214	29.810028	68.19874	LOC_Os03g30830.1         	glucan endo-1,3-beta-glucosidase 4 precursor	PR protein
gb_UNIGENE16109	0.4949846	0.0035167905	-0.70322865	732.765	1483.2622	0.55279624	0.011949801	-0.5927658	169.1265	303.99683	LOC_Os03g46660.1         	glucan endo-1,3-beta-glucosidase 7 precursor	PR protein
gb_UNIGENE1639	4.8301816	0.0013170724	1.574884	47455.977	10122.4375	4.284679	0.00024753078	1.4550456	37518.926	8742.889	LOC_Os01g51570.1         	glucan endo-1,3-beta-glucosidase GII precursor	PR protein
gb_UNIGENE11289	5.3663163	0.009053477	1.6801417	199.2375	37.18182	7.2531986	0.003944624	1.9814426	349.17	50.487602	LOC_Os01g71670.1         	glucan endo-1,3-beta-glucosidase GII precursor	PR protein
gb_UNIGENE1637	6.554939	0.0056140805	1.8802187	45826.1	6834.873	10.609137	0.00089549203	2.3617156	34643.75	3217.8542	LOC_Os01g71670.1         	glucan endo-1,3-beta-glucosidase GII precursor	PR protein
gb_UNIGENE1636	4.5644083	0.0014866886	1.5182889	35257.926	7913.5767	11.0481205	0.0004900561	2.4022603	25282	2176.0806	LOC_Os01g51570.1         	glucan endo-1,3-beta-glucosidase GII precursor	PR protein
gb_UNIGENE13350	8.020986	0.0039252965	2.0820613	3500.8901	438.12973	17.090124	0.000599045	2.8385007	2615.0474	148.3923	LOC_Os01g71340.1         	glucan endo-1,3-beta-glucosidase, acidic isoform precursor	PR protein
gb_UNIGENE5369	2.2209282	0.012909405	0.7979252	718.60547	342.41632	1.8345267	0.03557258	0.6067865	188.901	107.59347	LOC_Os03g18850.1         	pathogenesis-related protein 1	PR protein
gb_UNIGENE12046	7.3309975	0.00985818	1.9921116	3358.52	415.0314	12.183125	0.00050476077	2.5000517	2886.14	242.54138	LOC_Os07g03710.1         	pathogenesis-related protein PRB1-3 precursor	PR protein
gb_UNIGENE2211	7.6026583	0.00465991	2.028498	14062.848	1781.581	18.102495	0.00044944158	2.8960497	16781.8	929.0659	LOC_Os01g28500.1         	 Pathogenesis-related protein PRB1-2	PR protein
gb_UNIGENE2214	7.9269447	0.0065293885	2.0702677	25619.65	3051.276	13.004931	0.00052513235	2.5653286	16184.275	1259.0061	LOC_Os01g28500.1         	 Pathogenesis-related protein PRB1-2	PR protein
gb_UNIGENE2209	9.256841	0.0070260493	2.2253628	37321.7	3820.1013	15.392146	0.00062283425	2.7338574	23865.824	1566.1697	LOC_Os01g28500.1         	 Pathogenesis-related protein PRB1-2	PR protein
gb_UNIGENE2210	7.9980817	0.008106357	2.0792017	31218.527	3630.7324	16.986301	0.0002797545	2.8324072	27683.148	1628.2021	LOC_Os01g28500.1         	 Pathogenesis-related protein PRB1-2	PR protein
gb_UNIGENE2212	5.772924	0.0117868	1.7531787	25251.375	4144.0576	6.8057632	0.0129291555	1.9177698	13669.325	2301.3784	LOC_Os01g28500.1         	 Pathogenesis-related protein PRB1-2	PR protein
gb_UNIGENE4402	9.576117	0.0011332437	2.259272	3182.4924	344.66284	9.546108	0.00031424695	2.2561336	366.0805	37.38145	LOC_Os12g36830.1         	pathogenesis-related protein 10	PR protein
gb_UNIGENE4405	7.6923566	0.004611701	2.0402272	10357.055	1373.1593	8.030602	0.0011341686	2.0832596	5274.0625	690.59906	LOC_Os12g36830.1         	pathogenesis-related protein 10	PR protein
gb_UNIGENE4406	5.4224176	0.009996033	1.6905417	1767.5925	315.01227	7.388532	0.010126946	1.9999291	963.062	164.98637	LOC_Os12g36830.1         	pathogenesis-related protein 10	PR protein
gb_UNIGENE10205	0.49920458	0.038431354	-0.6947393	3828.6675	7372.7656	0.540722	0.0053925226	-0.6148499	3197.1025	5818.5293	LOC_Os04g50710.1         	pathogenesis-related 10 protein PR10-1, putative	PR protein
gb_UNIGENE10004	2.877006	0.001551898	1.0567502	2115.79	754.1064	2.0213873	0.008571852	0.7037841	758.26825	374.09763	LOC_Os03g14050.1         	thaumatin-like protein 1 precursor	PR protein
gb_UNIGENE3947	4.4646626	0.0013195252	1.4961936	111107.625	25347.18	4.265456	0.0000921718	1.4505491	114530	26955.057	LOC_Os12g43380.1         	thaumatin-like protein precursor	PR protein
gb_UNIGENE4056	11.863256	0.0016863089	2.473446	7774.9175	636.8822	22.420599	0.00034538584	3.10998	9493.314	430.60776	LOC_Os12g43430.1         	thaumatin-like protein precursor	PR protein
gb_UNIGENE4054	19.031073	0.0031469131	2.946073	114392.945	6908.5396	28.70804	0.00019899712	3.3571773	103004.2	3745.342	LOC_Os12g43430.1         	thaumatin-like protein precursor	PR protein
gb_UNIGENE10245	8.41005	0.0005334111	2.1294274	1752.0076	215.43488	8.538121	0.0011445221	2.144541	1302.0275	155.60207	LOC_Os11g07690.1         	disease resistance response protein 206, putative	PR protein
gb_UNIGENE5607	3.3527741	0.0068649137	1.2097881	2588.11	768.8317	9.966837	0.00020100786	2.2992632	18098.3	1827.2714	LOC_Os01g14590.1         	pathogen-related protein	PR protein
gb_UNIGENE5605	2.1637444	0.022277916	0.7718402	147.39613	67.56661	9.672752	0.0007042776	2.2693129	1158.81	126.07973	LOC_Os01g14590.1         	pathogen-related protein	PR protein
gb_UNIGENE13312	0.5495022	0.015219462	-0.59874254	655.6207	1156.5073	0.4598973	0.005168204	-0.77675205	281.71124	599.2782	LOC_Os03g64280.1         	1-aminocyclopropane-1-carboxylate oxidase	hormone metabolism
gb_UNIGENE2639	6.1386757	0.0006724933	1.814609	18447.725	2976.7002	4.7746477	0.0007413406	1.5633202	13120.95	2719.3093	LOC_Os05g05680.1         	1-aminocyclopropane-1-carboxylate oxidase	hormone metabolism
gb_UNIGENE2642	3.4836442	0.0031384963	1.248079	28078.9	8157.377	3.4002597	0.0015404188	1.2238518	31273.275	9185.903	LOC_Os05g05680.1         	1-aminocyclopropane-1-carboxylate oxidase	hormone metabolism
gb_UNIGENE2638	2.067129	0.00064571766	0.72616065	2682.5073	1285.6613	2.1509414	0.0025798266	0.76590556	2197.8574	1018.5137	LOC_Os02g53180.3         	1-aminocyclopropane-1-carboxylate oxidase 1	hormone metabolism
gb_UNIGENE2636	2.1502535	0.0017306581	0.7655857	1554.1875	737.75397	2.2278273	0.0013035851	0.80102676	1035.8569	466.05804	LOC_Os02g53180.2         	1-aminocyclopropane-1-carboxylate oxidase 1	hormone metabolism
gb_UNIGENE7980	0.42325765	0.03793071	-0.8597742	43.60653	100.91004	0.47056833	0.031139607	-0.7538141	73.64755	171.95598	LOC_Os06g14370.2         	ABA-induced protein	hormone metabolism
gb_UNIGENE15047	0.45937777	0.014250227	-0.7778824	581.8265	1204.8885	0.5540692	0.035736334	-0.5904656	414.58826	723.0522	LOC_Os02g04810.1         	auxin response factor 2	hormone metabolism
gb_UNIGENE2768	0.36026987	0.01029393	-1.0209019	13186.742	35908.766	0.2719501	0.016533138	-1.3021367	1985.8575	7630.059	LOC_Os08g35760.1         	auxin-binding protein ABP20 precursor	hormone metabolism
gb_UNIGENE2769	0.23820882	0.08434757	-1.4346076	7016.043	20166.613	0.18192102	0.018029762	-1.7041826	3428.7073	16031.73	LOC_Os08g35760.1         	auxin-binding protein ABP20 precursor	hormone metabolism
gb_UNIGENE2504	0.42328423	0.0026691912	-0.8597114	1352.024	3179.7944	0.48374805	0.001498168	-0.72619104	781.82996	1618.4932	LOC_Os03g53150.1         	OsIAA13 - Auxin-responsive Aux/IAA gene family member	hormone metabolism
gb_UNIGENE14901	0.041078232	0.009738626	-3.192277	95.452126	1153.2484	0.20077734	0.11046347	-1.6055588	250.16641	842.1475	LOC_Os07g29310.1         	OsSAUR30 - Auxin-responsive SAUR gene family member	hormone metabolism
gb_UNIGENE15250	2.0788867	0.007562405	0.7318325	6209.7227	3024.5479	1.8128303	0.034638636	0.59488934	8743.109	4910.775	LOC_Os09g37330.1         	OsSAUR39 - Auxin-responsive SAUR gene family member	hormone metabolism
gb_UNIGENE23196	2.195203	0.03350658	0.78627455	107.78642	45.445274	2.624317	0.022287458	0.9648206	92.697754	37.16186	LOC_Os01g45860.1         	DELLA protein RGA	hormone metabolism
gb_UNIGENE20431	4.589002	0.0014473761	1.5236626	803.18097	177.64227	4.3162622	0.00076109957	1.4623898	550.3405	124.1897	LOC_Os01g06060.1         	gibberellin receptor GID1L2	hormone metabolism
gb_UNIGENE23369	1.7859718	0.0014858557	0.5799627	348.74976	195.92963	2.118473	0.003619842	0.7506956	140.095	66.40585	LOC_Os02g35940.1         	gibberellin receptor GID1L2	hormone metabolism
gb_UNIGENE13248	3.582299	0.018357782	1.2760048	170.1445	46.218376	1.7886544	0.0065354486	0.58146363	203.302	112.91679	LOC_Os04g12970.1         	indole-3-acetate beta-glucosyltransferase	hormone metabolism
gb_UNIGENE19199	0.44404185	0.025757857	-0.8118365	2208.0525	5365.039	1.0226583	0.7073475	0.022405483	569.177	550.196	LOC_Os03g55040.1         	indole-3-acetate beta-glucosyltransferase	hormone metabolism
gb_UNIGENE15634	1.3607354	0.030939376	0.30802527	4098.1675	2988.232	2.1784215	0.00041513506	0.7786006	1252.675	575.89026	LOC_Os04g12980.1         	indole-3-acetate beta-glucosyltransferase	hormone metabolism
gb_UNIGENE18558	6.2585163	0.0008542547	1.8339431	508.862	85.34156	11.123887	0.00025704812	2.4090948	540.258	45.111248	LOC_Os05g35290.1         	phenylalanine ammonia-lyase	phynylpropanoid pathway
gb_UNIGENE1802	14.180684	0.00031975337	2.6518807	1017.9888	72.69894	14.602955	0.00059631426	2.6812239	882.46277	57.688087	LOC_Os04g43800.1         	phenylalanine ammonia-lyase	phynylpropanoid pathway
gb_UNIGENE1800	5.162065	0.00085459126	1.6413367	497.14673	102.8016	5.237345	0.009639895	1.6558148	1550.2833	275.50986	LOC_Os04g43800.1         	phenylalanine ammonia-lyase	phynylpropanoid pathway
gb_UNIGENE1805	7.561742	0.0012628371	2.0231016	2228.8225	307.37158	7.344238	0.0010097604	1.993916	2152.0376	282.8707	LOC_Os04g43800.1         	phenylalanine ammonia-lyase	phynylpropanoid pathway
gb_UNIGENE1795	11.960164	0.000077951285	2.4815814	540.4265	45.240177						LOC_Os04g43800.1         	phenylalanine ammonia-lyase	phynylpropanoid pathway
gb_UNIGENE1799	1.7385546	0.0014254277	0.5530541	782.31573	455.037	3.0584378	0.02717471	1.1179043	3435.585	1142.5046	LOC_Os04g43800.1         	phenylalanine ammonia-lyase	phynylpropanoid pathway
gb_UNIGENE1797	2.081267	0.009082482	0.7329769	20531.975	10266.298	2.4702015	0.0032700542	0.90429974	16103.5	6458.396	LOC_Os02g41650.2         	phenylalanine ammonia-lyase	phynylpropanoid pathway
gb_UNIGENE4346	2.867605	0.04799061	1.0534772	5204.0923	1892.8148	3.4557362	0.009042587	1.2400355	4118.1226	1113.7083	LOC_Os02g09490.1         	cinnamyl alcohol dehydrogenase	phynylpropanoid pathway
gb_UNIGENE7358	0.21748716	0.010763642	-1.5256155	358.58057	1315.8319	0.19417661	0.036134124	-1.6389872	178.97995	536.536	LOC_Os11g32650.1         	chalcone synthase	phynylpropanoid pathway
gb_UNIGENE15844	10.555268	0.000011976543	2.356625	1568.1125	148.17572	10.784408	0.0006284451	2.3781013	1584.4667	143.99825	LOC_Os08g34790.1         	4-coumarate--CoA ligase 2	phynylpropanoid pathway
gb_UNIGENE4676	2.752478	0.0041870913	1.0125016	5657.735	2113.5618	2.439889	0.0053496878	0.8919525	4440.4976	1789.0995	LOC_Os02g08100.1         	4-coumarate--CoA ligase 1	phynylpropanoid pathway
gb_UNIGENE3439	2.094131	0.022229163	0.73913866	111.3229	51.741486	2.208851	0.027476974	0.7924725	93.410126	43.477776	LOC_Os05g25640.1         	trans-cinnamate 4-monooxygenase	phynylpropanoid pathway
gb_UNIGENE606	3.8552644	0.0013612299	1.3494396	40084.547	10364.258	3.344815	0.00029581832	1.2074114	35738.75	10509.524	LOC_Os08g38900.3         	caffeoyl-CoA O-methyltransferase 1	phynylpropanoid pathway
gb_UNIGENE406	3.2108226	0.0014885523	1.1665272	938.03625	291.47147	2.630395	0.00094413536	0.967134	760.6537	284.4267	LOC_Os08g38900.1         	caffeoyl-CoA O-methyltransferase 1	phynylpropanoid pathway
gb_UNIGENE619	3.2332318	0.002067581	1.1734822	18925.35	6106.9717	2.800604	0.0017552812	1.0298351	12928.588	4590.844	LOC_Os06g06980.1         	caffeoyl-CoA O-methyltransferase 1	phynylpropanoid pathway
gb_UNIGENE2736						4.5493484	0.00029447954	1.514984	49.93995	11.001629	LOC_Os09g33680.1         	cyanogenic beta-glucosidase precursor	phynylpropanoid pathway
gb_UNIGENE19751	0.5947753	0.038706433	-0.51957154	403.75076	633.1429	0.39449936	0.033537656	-0.93013775	171.85802	407.19324	LOC_Os08g39870.1         	non-cyanogenic beta-glucosidase precursor	phynylpropanoid pathway
gb_UNIGENE13799	3.8445146	0.000058565587	1.3466474	749.8745	195.25836	2.2659638	0.018417101	0.81800014	680.42847	309.75967	LOC_Os03g08999.1         	alcohol dehydrogenase 1	Phenylalamine and tryptophan biosynthesis
gb_UNIGENE5638	6.552858	0.004727863	0.991488	14522.4	5291.223	2.4248211	0.0059488253	0.88575774	14725.35	5958.662	LOC_Os04g56910.1         	anthranilate N-benzoyltransferase protein 1	Phenylalamine and tryptophan biosynthesis
gb_UNIGENE5883	2.3841681	0.006365749	0.9749571	848.265	317.20587	1.6840106	0.044685405	0.52117825	883.86	539.36285	LOC_Os03g03450.1         	anthranilate phosphoribosyltransferase, chloroplast precursor	Phenylalamine and tryptophan biosynthesis
gb_UNIGENE8200	2.695242	0.027578996	1.3544946	580.1675	199.86638	4.4534984	0.002657738	1.4936899	327.99628	73.4221	LOC_Os04g38950.1         	anthranilate synthase component II	Phenylalamine and tryptophan biosynthesis
gb_UNIGENE11623	3.0323176	0.088422626	1.1093272	138.25172	51.386845	4.749582	0.000724977	1.5580566	478.98227	107.15112	LOC_Os08g04540.1         	aromatic-L-amino-acid decarboxylase	Phenylalamine and tryptophan biosynthesis
gb_UNIGENE6407	5.1774006	0.008958816	1.0712909	56457.203	19791.082	2.6332738	0.0034816968	0.9682279	36760.7	13877.527	LOC_Os08g23150.1         	indole-3-glycerol phosphate synthase, chloroplast precursor	Phenylalamine and tryptophan biosynthesis
gb_UNIGENE10115	5.0041504	0.0018478376	1.1336455	238.81276	76.14621	3.3523948	0.004247636	1.209675	165.432	50.99568	LOC_Os09g08130.2         	indole-3-glycerol phosphate synthase, chloroplast precursor	Phenylalamine and tryptophan biosynthesis
gb_UNIGENE18733	2.6510534	0.0014955678	1.6102676	3605.2625	731.25604	3.5284479	0.0005248934	1.260858	2436.92	701.43805	LOC_Os04g59520.1         	phosphoribosylanthranilate transferase	Phenylalamine and tryptophan biosynthesis
gb_UNIGENE3904	2.9191453	0.00071207725	1.6443031	3990.16	774.35004	5.5577416	0.00022285162	1.7151918	3029.9575	533.09155	LOC_Os08g04180.1         	tryptophan synthase beta chain 1	Phenylalamine and tryptophan biosynthesis
gb_UNIGENE17478	3.1069624	0.06288749	0.8688503	5514.798	2379.4226	2.3872273	0.009983678	0.87013257	3427.28	1419.0302	LOC_Os06g42560.3         	tryptophan synthase beta chain 2	Phenylalamine and tryptophan biosynthesis
gb_UNIGENE7634	3.4776008	0.0022134932	1.2463427	6195.9375	1828.9763	2.8674657	0.0009627262	1.0534286	5278.82	1821.2863	LOC_Os12g02960.1         	glutathione S-transferase	oxidative burst
gb_UNIGENE6333	2.245612	0.00924527	0.8089781	12287.7	5519.5366	2.8258836	0.0017481208	1.0388211	7367.7627	2609.253	LOC_Os03g04260.1         	glutathione S-transferase	oxidative burst
gb_UNIGENE2975	2.5035515	0.0057223137	0.91771036	18539.148	7269.2383	2.4923487	0.0031512766	0.91322553	24474.273	10088.155	LOC_Os01g27260.1         	glutathione S-transferase GSTF1	oxidative burst
gb_UNIGENE20831	0.48144656	0.030740287	-0.73096	5881.6177	11072.528	0.4655271	0.0330693	-0.764585	4271.1924	8562.881	LOC_Os01g27260.1         	glutathione S-transferase GSTF1	oxidative burst
gb_UNIGENE21026	7.7323236	0.0060443957	2.0454094	251.50226	35.767105	6.509452	0.009173652	1.8732553	204.65724	30.463287	LOC_Os01g27390.1         	glutathione S-transferase GSTF2	oxidative burst
gb_UNIGENE14304	5.2096615	0.00015427495	1.6505148	3944.5151	757.1795	4.9914136	0.0010332873	1.6077192	4953.455	961.8178	LOC_Os10g38470.1         	glutathione S-transferase GSTU6	oxidative burst
gb_UNIGENE7171	6.4367576	0.000637845	1.8620249	1142.6505	185.32663	4.7525883	0.00031226888	1.5586894	756.7908	158.1998	LOC_Os10g38710.1         	glutathione S-transferase GSTU6	oxidative burst
gb_UNIGENE14387	8.165297	0.008860123	2.099893	96.2962	10	5.3213544	0.0083518345	1.6717279	204.94101	38.923008	LOC_Os10g38590.1         	glutathione S-transferase GSTU6	oxidative burst
gb_UNIGENE12776	4.466654	0.011363326	1.4966395	34063.523	7548.5107	4.3301253	0.004219497	1.4655964	20563.3	4612.867	LOC_Os10g38740.1         	glutathione S-transferase GSTU6	oxidative burst
gb_UNIGENE6010	3.7912836	0.014452411	1.3327047	838.71747	198.13884	2.6604526	0.02478605	0.97849625	33.020077	11.30221	LOC_Os10g38150.1         	glutathione S-transferase GSTU6	oxidative burst
gb_UNIGENE13240	2.1901934	0.010012758	0.7839899	2075.0725	969.3726	1.6626042	0.042945765	0.5083852	1024.9653	624.0774	LOC_Os01g27210.1         	glutathione S-transferase IV	oxidative burst
gb_UNIGENE7415	4.190274	0.0010372763	1.4327661	310.7515	74.3281	6.9011507	0.0006584616	1.9316882	1220.93	174.421	LOC_Os02g21460.1         	glutathione S-transferase, C-terminal-like	oxidative burst
gb_UNIGENE7080	2.5894227	0.011752869	0.95143497	1190.171	448.4944	1.825436	0.123801	0.60181886	271.6035	140.4316	LOC_Os07g44550.1         	peroxidase 1 precursor	oxidative burst
gb_UNIGENE1859	5.748767	0.006268687	1.7489854	113.97618	19.001255	3.4085264	0.0125955725	1.2262801	34.7272	10	LOC_Os04g59150.1         	peroxidase 12 precursor	oxidative burst
gb_UNIGENE2112	12.159904	0.0006278869	2.498144	23761.477	2023.6609	12.557541	0.0017307812	2.5303214	23860.85	1883.4062	LOC_Os07g48030.1         	peroxidase 2 precursor	oxidative burst
gb_UNIGENE2114	4.1127687	0.0066230004	1.4140965	208.795	57.28582	5.174699	0.0023250543	1.6437812	156.83575	30.62396	LOC_Os07g48030.1         	peroxidase 2 precursor	oxidative burst
gb_UNIGENE4337	2.1970837	0.0072714062	0.7871309	1128.9843	511.4314	1.5846913	0.029185034	0.4603896	1165.6375	747.37006	LOC_Os01g22352.1         	peroxidase 2 precursor	oxidative burst
gb_UNIGENE2115	1.095905	0.4506109	0.091580436	1226.8492	1128.915	2.816511	0.011969251	1.0354989	3208.79	1073.5648	LOC_Os07g48010.1         	peroxidase 2 precursor	oxidative burst
gb_UNIGENE15211	0.24703929	0.011549877	-1.3982079	291.94263	1000.00696	0.23368864	0.010758444	-1.4537656	130.25198	482.71246	LOC_Os01g16450.1         	peroxidase 24 precursor	oxidative burst
gb_UNIGENE20678	0.22626054	0.085814685	-1.4860681	78.87981	367.15234	0.29921457	0.022118352	-1.2065943	25.224373	75.73082	LOC_Os01g28030.1         	peroxidase 24 precursor	oxidative burst
gb_UNIGENE11361	2.1291184	0.002085347	0.75570804	406.30176	191.35063	2.1901577	0.005061825	0.7839736	599.50525	274.27026	LOC_Os03g55410.1         	peroxidase 51 precursor	oxidative burst
gb_UNIGENE1865	0.28141424	0.062310044	-1.2679275	41.278843	110.52409	0.20267096	0.013716587	-1.5961715	38.652073	167.49722	LOC_Os10g02040.1         	peroxidase 54 precursor	oxidative burst
gb_UNIGENE9877	0.38236827	0.031785283	-0.9613711	327.451	798.957	0.49519497	0.021132126	-0.70280373	259.49448	520.6217	LOC_Os03g02920.1         	peroxidase 66 precursor	oxidative burst
gb_UNIGENE3239	3.350172	0.0073974878	1.2090117	75809.1	23085.098	2.0006986	0.00429181	0.69349635	86591.02	44413.42	LOC_Os02g14430.1         	peroxidase 68 precursor	oxidative burst
gb_UNIGENE3243	2.0627062	0.023818666	0.7240188	34357.074	16693.701	1.6704118	0.009507145	0.51307017	42864.824	26243.621	LOC_Os02g14430.1         	peroxidase 68 precursor	oxidative burst
gb_UNIGENE2119	25.117208	0.0024010139	3.2235532	14602.792	545.19116	81.34274	0.0007211291	4.3986716	8595.133	103.93672	LOC_Os07g48050.1         	peroxidase precursor	oxidative burst
gb_UNIGENE2118	15.018366	0.0038838314	2.7092738	14169.533	892.8946	32.00728	0.0011075152	3.4659634	20766.049	633.296	LOC_Os07g48050.1         	peroxidase precursor	oxidative burst
gb_UNIGENE2123	13.683052	0.00220215	2.616158	4067.635	268.71536	1.0373605	0.8816769	0.036679532	29.959665	34.771427	LOC_Os07g48050.1         	peroxidase precursor	oxidative burst
gb_UNIGENE15108	5.356895	0.003689175	1.6783845	69.1089	12.145919	8.909345	0.006992898	2.1871006	168.37148	17.073513	LOC_Os04g42100.1         	peroxidase 18 precursor	oxidative burst
gb_UNIGENE4131	2.0544803	0.004548063	0.7200229	11665.22	5668.495	1.7409276	0.0011964877	0.554418	7922.34	4568.5576	LOC_Os04g35520.3         	OsAPx7 - Stromal Ascorbate Peroxidase encoding gene	oxidative burst
gb_UNIGENE4135	0.48786527	0.036894742	-0.717716	4258.8975	8203.1455	0.45577425	0.013794428	-0.78575766	3667.0474	7789.337	LOC_Os02g34810.1         	OsAPx8 - Thylakoid-bound Ascorbate Peroxidase encoding gene	oxidative burst
gb_UNIGENE24429	2.4291208	0.000082036495	0.8875294	652.8415	268.63116	1.795583	0.0027156963	0.5853298	277.19623	156.54428	LOC_Os05g34220.2         	disease resistance protein	signal perception and transduction 
gb_UNIGENE12245	3.251534	0.0009766322	1.1791269	1061.4325	326.892	2.481134	0.00007503931	0.9087157	895.174	357.88394	LOC_Os11g12320.1         	disease resistance protein RPM1, putative	signal perception and transduction 
gb_UNIGENE20639	5.0824857	0.0014511426	1.6258005	62.79755	12.360829	5.166359	0.0023155247	1.6421682	65.7334	13.072781	LOC_Os01g41750.1         	leucine-rich repeat receptor protein kinase EXS precursor	signal perception and transduction 
gb_UNIGENE11866	0.6620942	0.2690362	-0.4123475	25.382626	42.06569	4.542111	0.0015076216	1.5133919	28641.975	6298.926	LOC_Os02g40240.2         	leucine-rich repeat receptor protein kinase EXS precursor	signal perception and transduction 
gb_UNIGENE22050	0.47162622	0.054206762	-0.7515685	683.14575	1388.5732	0.38240647	0.022273868	-0.96127117	377.04373	966.0206	LOC_Os03g43650.1         	leucine-rich repeat receptor protein kinase EXS precursor	signal perception and transduction 
gb_UNIGENE13968	6.1484575	0.0000266437	1.8162012	75.946625	12.4625	3.9402153	0.026780333	1.3712354	56.059452	13.216111	LOC_Os01g66510.1         	MLO-like protein 1	signal perception and transduction 
gb_UNIGENE19979	11.688312	0.0006103717	2.4585893	520.4845	44.860523	14.898376	0.0033850982	2.7012522	448.48273	33.68691	LOC_Os04g43440.1         	NB-ARC domain containing protein	signal perception and transduction 
gb_UNIGENE25955	2.1338496	0.02211577	0.7579277	311.26123	148.03513	1.4419204	0.0586461	0.3659758	225.54901	154.46274	LOC_Os01g70080.1         	NB-ARC domain containing protein	signal perception and transduction 
gb_UNIGENE19197	9.814479	0.0030214312	2.2838588	1354.5275	142.84859	12.385633	0.0029657613	2.5165372	1184.4065	98.15184	LOC_Os04g43440.1         	NB-ARC domain containing protein	signal perception and transduction 
gb_UNIGENE17047	3.2338316	0.000967456	1.1736677	945.5767	290.53165	2.3709624	0.0048659234	0.8632959	799.3822	330.34973	LOC_Os01g23380.1         	stripe rust resistance protein Yr10	signal perception and transduction 
gb_UNIGENE23814	2.390277	0.0009469824	0.87140924	2817.0024	1186.3153	1.588703	0.0059308214	0.462918	2055.915	1296.7291	LOC_Os01g04070.1         	verticillium wilt disease resistance protein	signal perception and transduction 
gb_UNIGENE15476	3.7918022	0.002374538	1.3328414	3781.8975	1015.4836	2.9923239	0.007446216	1.0960503	3095.3926	1015.00793	LOC_Os07g35390.1         	serine/threonine kinase-like protein	signal perception and transduction 
gb_UNIGENE6958	3.498602	0.00015679646	1.2523634	2438.1326	692.6774	6.898315	0.00016891949	1.9312772	664.9492	96.52722	LOC_Os07g35260.1         	serine/threonine kinase-like protein	signal perception and transduction 
gb_UNIGENE25812	4.2688317	0.0007534698	1.4513402	2092.78	478.17688	3.2198796	0.00039749892	1.169344	2217.5825	686.9534	LOC_Os06g51170.1         	serine/threonine-protein kinase Cx32, chloroplast precursor	signal perception and transduction 
gb_UNIGENE17408	2.6667206	0.00022981096	0.9808495	311.2995	115.79497	2.421575	0.0026403135	0.8844182	203.5805	85.337616	LOC_Os09g36320.1         	serine/threonine-protein kinase NAK	signal perception and transduction 
gb_UNIGENE24256	3.0694265	0.00051671587	1.1214907	3753.1775	1203.0941	2.4103754	0.0028092724	0.87978244	2814.5676	1160.3286	LOC_Os01g60700.2         	serine/threonine-protein kinase NAK	signal perception and transduction 
gb_UNIGENE15336	3.757973	0.0014774323	1.3238797	2046.3151	543.7845	2.9714196	0.00096998224	1.0890398	1195.6565	402.99643	LOC_Os04g01310.1         	serine/threonine-protein kinase receptor precursor	signal perception and transduction 
gb_UNIGENE19683	2.8887384	0.0067471494	1.0608199	4669.9375	1587.6512	2.1920567	0.007833724	0.7848402	3685.7625	1687.9935	LOC_Os04g52840.1         	serine/threonine-protein kinase receptor precursor	signal perception and transduction 
gb_UNIGENE13499	0.074705034	no replicates	-2.5942078	1.81974	24.359	3.0797534	0.0020859516	1.1248496	338.60925	111.33784	LOC_Os06g47530.1         	serine/threonine-protein kinase receptor precursor	signal perception and transduction 
gb_UNIGENE21140	2.5432703	0.022003882	0.9334508	47.64617	19.53114	3.0157697	0.008082876	1.1038551	34.508247	10.983998	LOC_Os01g57480.1         	serine/threonine-protein kinase receptor precursor	signal perception and transduction 
gb_UNIGENE18906	5.491418	0.00082570955	1.7031865	2410.975	436.89145	4.2696013	0.00068470725	1.4515204	2336.0576	534.6536	LOC_Os07g35660.1         	receptor-like serine-threonine protein kinase	signal perception and transduction 
gb_UNIGENE13644	3.2335277	0.0010749582	1.1735737	320.05273	99.84977	3.3749893	0.0046951603	1.2163922	297.81024	89.001175	LOC_Os07g35660.1         	receptor-like serine-threonine protein kinase	signal perception and transduction 
gb_UNIGENE13429						2.6883733	0.02597228	0.9889363	782.69275	285.9581	LOC_Os01g02400.3         	Ser/Thr receptor-like kinase	signal perception and transduction 
gb_UNIGENE13428	5.181894	0.0033412366	1.6451706	611.73926	114.81212	1.9799099	0.05174483	0.68305135	293.80753	144.12248	LOC_Os01g02400.2         	Ser/Thr receptor-like kinase	signal perception and transduction 
gb_UNIGENE24896	2.3257914	0.0006753339	0.8440603	12243.475	5264.0815	1.8912816	0.0009732835	0.6372547	13492.899	7168.208	LOC_Os06g05520.1         	OsMKK1 - putative MAPKK based on amino acid sequence homology	signal perception and transduction 
gb_UNIGENE6993	3.211006	0.00032521304	1.1665843	349.29	110.187874	2.0381992	0.000056154247	0.71206665	931.4983	456.46255	LOC_Os10g38950.1         	OsMPK4 - putative MAPK based on amino acid sequence homology	signal perception and transduction 
gb_UNIGENE12629	3.5848866	0.0061262543	1.2767268	1368.635	385.12613	3.058541	0.008834909	1.117938	1151.7937	383.57373	LOC_Os01g49529.3         	OsWAK10a - OsWAK receptor-like cytoplasmic kinase (OsWAK-RLCK)	signal perception and transduction 
gb_UNIGENE14031	10.207785	0.0009225556	2.3231506	4814.4077	464.215	7.1195607	0.002137396	1.962846	7796.5327	1106.9138	LOC_Os02g42150.2         	OsWAK14 - OsWAK receptor-like protein kinase	signal perception and transduction 
gb_UNIGENE11886	1.5611819	0.052034866	0.44544318	420.93625	279.7416	3.086877	0.0035244157	1.12716	2466.715	804.20215	LOC_Os04g24220.3         	OsWAK32 - OsWAK receptor-like protein kinase	signal perception and transduction 
gb_UNIGENE12770	6.3384967	0.0057918825	1.8466417	237.6605	40.288147	7.5968924	0.018312858	2.0277393	191.97351	29.226173	LOC_Os09g29510.1         	OsWAK80 - OsWAK receptor-like protein kinase	signal perception and transduction 
gb_UNIGENE16619	16.766373	0.000003876142	2.8193753	6561.093	388.43182	11.090114	0.00081346853	2.406054	9424.397	877.27563	LOC_Os09g29520.1         	OsWAK81 - OsWAK receptor-like cytoplasmic kinase (OsWAK-RLCK)	signal perception and transduction 
gb_UNIGENE10767	0.57355493	0.11114864	-0.5559016	89.37155	154.73056	0.44681063	0.012964315	-0.80562043	50.514503	106.20888	LOC_Os03g12730.1         	receptor protein kinase CLAVATA1 precursor	signal perception and transduction 
gb_UNIGENE24099	2.5053613	0.002095774	0.91843295	138.7775	55.547085	2.5912113	0.001954466	0.9521255	116.29762	45.558613	LOC_Os03g43670.1         	receptor protein kinase CRINKLY4 precursor	signal perception and transduction 
gb_UNIGENE26172	2.326856	0.057352792	0.844518	47.677174	22.413979	3.1449971	0.0006368548	1.145813	423.36224	131.79005	LOC_Os03g61060.2         	receptor protein kinase TMK1 precursor	signal perception and transduction 
gb_UNIGENE7933	3.9995775	0.000534891	1.3861887	22493.176	5659.2017	3.0169964	0.0011404958	1.1042618	14771.65	4929.76	LOC_Os03g04110.1         	receptor-like GPI-anchored protein 2	signal perception and transduction 
gb_UNIGENE7934	3.0433218	0.009576564	1.1129496	188.24924	66.39392	2.900663	0.025712434	1.0649393	101.6385	39.1988	LOC_Os03g04110.2         	receptor-like GPI-anchored protein 2	signal perception and transduction 
gb_UNIGENE18445	2.015035	0.0006411223	0.7006365	1680.9624	839.2554	1.8061074	0.012011068	0.59117395	1428.355	790.28436	LOC_Os04g54070.1         	receptor-like kinase	signal perception and transduction 
gb_UNIGENE22773	4.840409	0.013918094	1.5769992	182.30469	34.88612	5.006945	0.01341629	1.610826	103.11655	23.36245	LOC_Os06g41560.1         	receptor-like protein kinase	signal perception and transduction 
gb_UNIGENE13611	0.3643625	0.014242466	-1.009606	535.6958	1393.7433	0.68425333	0.02880526	-0.37942705	1803.695	2567.2393	LOC_Os04g12540.1         	receptor-like protein kinase	signal perception and transduction 
gb_UNIGENE10292	2.2250466	0.00829832	0.7997779	4530.295	2036.4082	2.0598626	0.021027418	0.7226393	2522.2925	1280.8911	LOC_Os09g17630.1         	receptor-like protein kinase 2	signal perception and transduction 
gb_UNIGENE13821	4.5809073	0.0024970653	1.5218971	439.60623	96.18989	4.227554	0.000729302	1.4416236	367.49625	86.09614	LOC_Os05g44770.1         	receptor-like protein kinase 5 precursor	signal perception and transduction 
gb_UNIGENE20719	2.4621482	0.002660717	0.90103424	4865.185	2008.4662	2.0705621	0.0037396457	0.7278201	4882.455	2360.8066	LOC_Os06g36270.5         	receptor-like protein kinase 5 precursor	signal perception and transduction 
gb_UNIGENE18966	0.41904044	0.01747577	-0.8697879	706.6887	1650.3569	0.46111393	0.021781996	-0.77411014	518.4978	1141.8607	LOC_Os06g10230.2         	receptor-like protein kinase 5 precursor	signal perception and transduction 
gb_UNIGENE4998	2.2976456	0.025371872	0.831885	698.2065	296.57593	2.231718	0.004063426	0.8027717	1519.1825	685.21716	LOC_Os01g02800.1         	receptor-like kinase ARK1AS	signal perception and transduction 
gb_UNIGENE13692	2.3017569	0.00024503918	0.83367264	5038.0073	2197.8833	0.9693523	0.8593845	-0.03112715	1187.9773	1204.9924	LOC_Os01g02800.1         	receptor-like kinase ARK1AS	signal perception and transduction 
gb_UNIGENE5000	3.584894	0.008605556	1.2767289	114.8166	33.869606	1.7649307	0.0104068965	0.5681114	2039.7574	1155.9258	LOC_Os01g02800.1         	receptor-like kinase ARK1AS	signal perception and transduction 
gb_UNIGENE13693	5.2772527	0.000373007	1.6634057	7533.4824	1433.6992	1.6452156	0.043463383	0.49787146	155.17642	90.93436	LOC_Os01g02800.1         	receptor-like kinase ARK1AS	signal perception and transduction 
gb_UNIGENE10743	2.092558	0.0014388465	0.73838717	462.5975	221.99713	1.4925679	0.0018882519	0.40049806	424.62476	285.18436	LOC_Os01g02350.1         	receptor-like kinase ARK1AS	signal perception and transduction 
gb_UNIGENE21770	7.381911	0.000045448836	1.9990325	1614.18	222.45657	3.0702739	0.0828306	1.1217668	107.88115	51.950306	LOC_Os05g41370.1         	receptor-like protein kinase homolog RK20-1	signal perception and transduction 
gb_UNIGENE6204	5.1061215	0.00008558474	1.6304401	15084.601	2925.002	2.4913366	0.004462476	0.9128194	22860.1	9241.103	LOC_Os02g40180.1         	receptor-like protein kinase precursor	signal perception and transduction 
gb_UNIGENE6205	6.5226517	0.000086375796	1.875281	16200.85	2506.2358						LOC_Os02g40180.1         	receptor-like protein kinase precursor	signal perception and transduction 
gb_UNIGENE6203	3.5274448	0.011913767	1.2605737	213.1655	57.568157	0.9759125	0.9219565	-0.024382323	14714.801	15558.747	LOC_Os02g40200.2         	receptor-like protein kinase precursor	signal perception and transduction 
gb_UNIGENE19845	2.5845683	0.01292425	0.94955844	81.2396	33.056137	1.9342067	0.00257596	0.6596973	580.3565	304.3539	LOC_Os02g40200.2         	receptor-like protein kinase precursor	signal perception and transduction 
gb_UNIGENE14243	1.6296285	0.1838617	0.48835212	68.53587	47.775482	4.597195	0.018814921	1.5254464	303.34326	68.026566	LOC_Os09g18010.1         	receptor-like protein kinase, putative	signal perception and transduction 
gb_UNIGENE19911	30.766088	0.00039487568	3.426413	1327.8041	43.56608	18.58085	0.00007158361	2.9221315	4099.61	222.89407	LOC_Os05g44970.1         	senescence-induced receptor-like serine/threonine-protein kinase precursor, putative	signal perception and transduction 
gb_UNIGENE9788	0.43840453	0.039176833	-0.8246132	126.72267	245.99466	0.41084936	0.040745944	-0.88952863	138.72949	347.221	LOC_Os09g15480.1         	Ser/Thr-rich protein T10 in DGCR region	signal perception and transduction 
gb_UNIGENE3635	13.10313	0.00015084377	2.5728512	25894.227	1988.531	10.841216	0.0003454432	2.3833551	12967.85	1198.417	LOC_Os11g31540.1         	BRASSINOSTEROID INSENSITIVE 1-associated receptor kinase 1 precursor	signal perception and transduction 
gb_UNIGENE3636	15.391042	0.00031383446	2.7337856	22389.576	1483.0164	12.854483	0.0005126474	2.5536926	7600.907	603.5531	LOC_Os11g31540.1         	BRASSINOSTEROID INSENSITIVE 1-associated receptor kinase 1 precursor	signal perception and transduction 
gb_UNIGENE5422	2.7113829	0.001571528	0.99745876	1161.45	431.51923	2.2726138	0.0016583756	0.8209306	926.7778	404.68832	LOC_Os04g38480.1         	BRASSINOSTEROID INSENSITIVE 1-associated receptor kinase 1 precursor	signal perception and transduction 
gb_UNIGENE3638	6.527903	0.008217056	1.8760858	386.54602	59.27697	10.754908	0.0023327507	2.3753622	929.8533	85.70728	LOC_Os11g31540.1         	BRASSINOSTEROID INSENSITIVE 1-associated receptor kinase 1 precursor	signal perception and transduction 
gb_UNIGENE10249	2.6968846	0.004340133	0.99209726	574.48773	217.00317	1.8928422	0.011986167	0.6380795	699.4182	376.08853	LOC_Os05g34270.1         	BRASSINOSTEROID INSENSITIVE 1-associated receptor kinase 1 precursor	signal perception and transduction 
gb_UNIGENE16157	4.5799375	0.00033160608	1.5216854	373.258	81.82588	3.73334	0.0016566941	1.3173033	339.97525	92.16037	LOC_Os02g46090.1         	calcium-dependent protein kinase, isoform AK1	signal perception and transduction 
gb_UNIGENE4589	3.8021507	0.0017236224	1.3355669	19369.125	5172.374	2.6314816	0.0063485666	0.96754706	16373.175	6301.2695	LOC_Os04g49510.1         	calcium-dependent protein kinase, isoform AK1	signal perception and transduction 
gb_UNIGENE5926	0.3196663	0.006231262	-1.1404777	215.48724	668.38965	0.45759252	0.01319505	-0.7817762	201.83826	458.74576	LOC_Os12g02200.1         	CBL-interacting serine/threonine-protein kinase 15	signal perception and transduction 
gb_UNIGENE5924	0.24937065	0.004963865	-1.3888149	114.65993	436.18622	0.44352037	0.037812192	-0.8130116	140.61832	315.62787	LOC_Os12g02200.1         	CBL-interacting serine/threonine-protein kinase 15	signal perception and transduction 
gb_UNIGENE11154	3.4411988	0.0012787969	1.2358199	10367.654	3003.0146	3.0621989	0.0012188893	1.1191332	8170.16	2658.9268	LOC_Os02g03410.2         	CDPK-related protein kinase	signal perception and transduction 
gb_UNIGENE7189	2.039918	0.020410586	0.7129096	4352.1953	2174.8425	1.5524144	0.0080297785	0.4398114	3182.6575	2061.987	LOC_Os11g28340.1         	ER lumen protein retaining receptor	signal perception and transduction 
gb_UNIGENE13217	2.775195	0.002731247	1.020721	631.706	218.78384	3.6079779	0.0010076313	1.2831475	524.4883	138.3848	LOC_Os07g36570.1         	KI domain interacting kinase 1	signal perception and transduction 
gb_UNIGENE19663	2.536296	0.030680694	0.9307047	74.561905	33.36766	2.71728	0.03750635	0.99963135	81.194176	32.702156	LOC_Os10g30600.2         	protein kinase APK1A, chloroplast precursor	signal perception and transduction 
gb_UNIGENE15156	2.5091093	0.00061281445	0.91992784	1610.4951	652.6238	2.2746994	0.00551306	0.8218479	1507.86	665.0507	LOC_Os03g08170.1         	protein kinase APK1B, chloroplast precursor	signal perception and transduction 
gb_UNIGENE24439	3.06172	0.00912068	1.1189768	407.97726	138.9317	3.1637037	0.009031489	1.1517434	378.32727	114.56976	LOC_Os02g43430.1         	protein kinase APK1B, chloroplast precursor	signal perception and transduction 
gb_UNIGENE13748	5.3614907	0.000089696776	1.679242	3690.205	689.18835	1.8558779	0.0018128481	0.61835784	1254.9025	678.5995	LOC_Os07g38800.1         	lectin-like receptor kinase 7	signal perception and transduction 
gb_UNIGENE13749	5.3557005	0.0006604804	1.6781615	2565.2175	482.88312	1.8988459	0.0041435705	0.64124626	904.68176	478.72723	LOC_Os07g38800.1         	lectin-like receptor kinase 7	signal perception and transduction 
gb_UNIGENE13747	2.2685108	0.0063767903	0.8191236	1181.3099	527.8432	1.5090367	0.01228242	0.4114715	675.601	444.759	LOC_Os07g38800.1         	lectin-like receptor kinase 7	signal perception and transduction 
gb_UNIGENE12442	2.3028767	0.0008462659	0.83415914	1551.4375	668.37	1.9006482	0.0016084305	0.642195	1392.88	736.3725	LOC_Os03g04270.1         	Leucine Rich Repeat family protein	signal perception and transduction 
gb_UNIGENE9408	11.545199	0.000056921177	2.4462698	2005.365	176.31952	14.689242	0.0001405681	2.6871154	4791.725	310.93207	LOC_Os08g42580.4         	lysM receptor-like kinase	signal perception and transduction 
gb_UNIGENE12751	2.2297866	0.008361273	0.8019059	596.4908	263.5553	2.215843	0.05371216	0.7956329	461.06024	193.10286	LOC_Os01g72690.2         	NAD kinase 1	signal perception and transduction 
gb_UNIGENE11274	0.4627475	0.004638722	-0.7705737	196.8235	431.67218	0.5364232	0.004409019	-0.6228319	57.227726	106.98234	LOC_Os05g32210.1         	NAD kinase 3	signal perception and transduction 
gb_UNIGENE7894	2.1521993	0.0061837323	0.7664902	1095.4535	498.13412	1.932666	0.01585676	0.6589004	968.45123	495.38596	LOC_Os09g03620.1         	nodulation receptor kinase precursor	signal perception and transduction 
gb_UNIGENE20799	2.0565565	0.0091266325	0.721033	629.2175	313.32623	1.627736	0.006559489	0.48719007	506.94226	311.6513	LOC_Os02g05950.1         	phytosulfokine receptor precursor	signal perception and transduction 
gb_UNIGENE24211	2.1186404	0.020733275	0.7507746	387.02374	182.19633	1.4400353	0.03133258	0.36466768	414.43903	290.69305	LOC_Os03g30890.2         	S-locus-like receptor protein kinase	signal perception and transduction 
gb_UNIGENE477	3.70321	0.00015348653	1.3092	24859.975	6736.996	2.7966115	0.0024953922	1.0284085	17216.676	6076.788	LOC_Os02g46830.1         	expressed protein	signal perception and transduction 
gb_UNIGENE11517	3.0032866	0.00015970728	1.0997072	8511.793	2861.988	2.310421	0.0076749017	0.83742976	6783.9297	2960.2192	LOC_Os12g33100.1         	guanylate kinase	signal perception and transduction 
gb_UNIGENE21786	6.186598	0.00036741805	1.8223853	4761.43	777.84326	5.5935616	0.00094047515	1.7216163	3370.5376	594.6891	LOC_Os01g49580.1         	protein kinase domain containing protein	signal perception and transduction 
gb_UNIGENE12959	0.39958572	0.03352478	-0.9173269	100.0159	250.34512	0.36367276	0.0005613331	-1.0115008	80.951355	223.04121	LOC_Os02g47420.3         	pollen-specific kinase partner protein	signal perception and transduction 
gb_UNIGENE14572	11.54174	0.00059628644	2.44597	1774.4949	163.81616	4.21163	0.004217519	1.4378498	3234.2024	768.74396	LOC_Os10g04730.1         	protein kinase	signal perception and transduction 
gb_UNIGENE14350	3.3745012	0.0011738067	1.2162476	10088.93	2974.1387	2.877541	0.0008796091	1.0569361	8253.412	2834.9556	LOC_Os05g25450.1         	protein kinase	signal perception and transduction 
gb_UNIGENE13755	2.5982559	0.0024389757	0.9548404	4483.9624	1747.6279	2.2278907	0.006236978	0.80105525	2754.9849	1252.755	LOC_Os04g44910.1         	protein kinase	signal perception and transduction 
gb_UNIGENE13253	9.715779	0.00017308291	2.2737513	2038.55	214.82689	1.2516078	0.13890487	0.22442892	43.353477	33.43698	LOC_Os09g16540.1         	protein kinase	signal perception and transduction 
gb_UNIGENE18712	5.6604333	0.000087210625	1.7335005	235.371	41.568237	6.655076	0.0006398063	1.8953799	512.44025	78.41288	LOC_Os12g41540.1         	protein kinase	signal perception and transduction 
gb_UNIGENE8557	4.599436	0.00009501251	1.5259336	8674.586	1917.4784	3.6960883	0.001929759	1.307275	6398.4697	1740.3613	LOC_Os05g02200.1         	protein kinase	signal perception and transduction 
gb_UNIGENE24190	9.336902	0.0012444364	2.2339745	506.47028	59.201027	6.672006	0.0004739772	1.8979206	574.531	86.13074	LOC_Os12g41530.1         	protein kinase	signal perception and transduction 
gb_UNIGENE20782	2.8720434	0.0014415585	1.0550238	831.563	286.04083	3.0971556	0.00006716604	1.1304841	382.295	121.261024	LOC_Os10g04730.1         	protein kinase	signal perception and transduction 
gb_UNIGENE18530	4.302842	0.0025846087	1.4592757	2384.88	561.93744	5.6055737	0.013496841	1.7237614	782.6737	169.6764	LOC_Os04g22120.1         	protein kinase	signal perception and transduction 
gb_UNIGENE13252	8.952853	0.00013292149	2.1919723	3159.2603	356.57617	1.3458029	0.07378948	0.29699078	77.16398	58.228554	LOC_Os09g16540.1         	protein kinase	signal perception and transduction 
gb_UNIGENE12732	2.0083268	0.0005723426	0.6973019	1226.585	611.44745	1.6532091	0.018369004	0.5027183	1034.2185	630.2907	LOC_Os12g07590.2         	receptor-type tyrosine-protein phosphatase S precursor	signal perception and transduction 
gb_UNIGENE10887	2.141408	0.031907193	0.7614635	641.4502	299.67212	2.196007	0.01643693	0.7866407	673.06226	304.1227	LOC_Os04g11820.1         	ABC transporter-like protein	transporter
gb_UNIGENE12753	15.2825	0.00013558778	2.7267084	6272.1	411.5941	21.43006	0.000034030552	3.0647945	19858.125	911.3539	LOC_Os01g24010.1         	PDR5-like ABC transporter	transporter
gb_UNIGENE8122	4.0110545	0.00043178487	1.3890542	8116.675	2035.6875	3.7345731	0.0037828134	1.3176335	7009.8247	1949.2697	LOC_Os01g42410.1         	PDR5-like ABC transporter	transporter
gb_UNIGENE13986	3.2585328	0.007907882	1.181277	826.7595	255.86743	3.548727	0.043785933	1.2665889	1105.005	252.70297	LOC_Os01g42380.1         	PDR5-like ABC transporter	transporter
gb_UNIGENE21298	3.9477718	0.008231028	1.3731513	315.39423	80.96386	5.718019	0.00768811	1.7436224	183.79326	32.560223	LOC_Os01g50100.1         	multidrug resistance protein 4	transporter
gb_UNIGENE18361	0.50718105	0.0029969674	-0.6788872	323.113	626.30585	0.49242494	0.0040075555	-0.70841324	233.395	470.50244	LOC_Os01g50100.1         	multidrug resistance protein 4	transporter
gb_UNIGENE9422	3.1073833	0.0014441642	1.133781	237.93976	76.457214	2.4029398	0.0013605817	0.8766929	192.87349	79.5756	LOC_Os01g07870.1         	multidrug resistance-associated protein 3	transporter
gb_UNIGENE20253	0.4350518	0.010565386	-0.8322902	1006.396	2152.171	0.69513893	0.0031863733	-0.3636436	1847.1001	2669.7732	LOC_Os12g30040.1         	amino acid transporter-like protein 1	transporter
gb_UNIGENE18841	0.3966797	0.024326399	-0.9246261	415.97473	1129.9722	0.6343904	0.03990814	-0.45509076	619.534	979.6841	LOC_Os01g61044.2         	amino acid-polyamine transporter	transporter
gb_UNIGENE22563	2.4054751	0.021704191	0.8777474	438.24927	191.95653	0.9169219	0.5635399	-0.08673295	90.36035	101.365685	LOC_Os03g62200.1         	ammonium transporter 2	transporter
gb_UNIGENE7944	5.160737	0.0056613507	1.6410794	29306.025	5897.1553	4.4402266	0.0022391423	1.4907054	25417.8	5694.8555	LOC_Os04g10750.4         	inorganic phosphate transporter 1-5	transporter
gb_UNIGENE17497	2.250616	0.005874218	0.81120396	406.1665	188.67717	2.435123	0.022634242	0.8899973	165.93475	81.08633	LOC_Os07g15370.1         	metal transporter Nramp6	transporter
gb_UNIGENE18326	2.8231614	0.036974218	1.0378573	166.71825	64.068146	1.543504	0.01426535	0.43405512	113.2073	75.995255	LOC_Os09g29050.1         	mitochondrial 2-oxoglutarate/malate carrier protein, putative	transporter
gb_UNIGENE10285	2.121771	0.0062005445	0.7522512	344.70175	166.49002	1.7044907	0.01043339	0.53326637	344.66275	202.37613	LOC_Os03g36730.1         	oligosaccharide transporter	transporter
gb_UNIGENE17575	0.36698	0.037893094	-1.002448	1486.6421	3793.33	0.40419146	0.010524608	-0.9058666	2068.6968	4600.5283	LOC_Os07g37454.1         	organic cation transporter 3	transporter
gb_UNIGENE25386	19.17622	0.00018400788	2.953671	2293.595	118.72974	23.77733	0.00017895576	3.1687326	1512.8301	58.919033	LOC_Os02g47090.1         	peptide transporter PTR2	transporter
gb_UNIGENE12317	3.166086	0.0053120917	1.1524961	5415.435	1679.1095	2.8480873	0.0046987063	1.0466477	3289.815	1164.9095	LOC_Os06g15370.1         	peptide transporter PTR2	transporter
gb_UNIGENE19439	3.039224	0.014535987	1.1116022	190.58951	65.45833	1.2993847	0.08633589	0.26189083	467.3575	369.00815	LOC_Os01g67630.2         	peptide transporter PTR2	transporter
gb_UNIGENE26311	0.63989925	0.048078515	-0.4464445	216.83775	322.59326	0.47055775	0.0005824205	-0.7538366	107.352394	231.03595	LOC_Os10g22560.3         	peptide transporter PTR2	transporter
gb_UNIGENE21643	2.2872148	0.056015	0.82733476	2475.595	1125.6283	4.8141537	0.0070868316	1.5715603	4165.4727	879.48804	LOC_Os03g04570.1         	peptide transporter PTR2	transporter
gb_UNIGENE14224						0.49591345	0.020138666	-0.70135385	28.7871	58.14338	LOC_Os03g10090.1         	polyol transporter protein 4	transporter
gb_UNIGENE6920	2.2819226	0.009562785	0.8250183	948.9598	412.0817	2.0239186	0.01922581	0.70503557	760.9967	381.29834	LOC_Os04g32920.5         	potassium transporter 1	transporter
gb_UNIGENE6707	9.812951	0.00011953572	2.283703	44220.953	4596.1504	7.7651362	0.00046265015	2.049644	37928.25	4793.1953	LOC_Os07g39350.1         	proton myo-inositol cotransporter	transporter
gb_UNIGENE6706	7.0557895	0.00014685992	1.9538485	2719.61	383.4002	5.7434387	0.0005555057	1.7480581	3208.18	556.3587	LOC_Os03g10100.1         	sorbitol transporter	transporter
gb_UNIGENE9827	2.6574302	0.045717996	0.97735953	34.580254	14.402338	4.2227626	0.0037647828	1.4404895	224.54175	55.995247	LOC_Os08g31410.7         	sulfate transporter 1.2	transporter
gb_UNIGENE12360	2.507206	0.003279409	0.919169	599.5088	238.37807	2.2044027	0.0022697733	0.79045653	527.392	237.61307	LOC_Os02g46962.3         	protein transporter	transporter
gb_UNIGENE26252	0.4459739	0.018698502	-0.8074948	811.89374	1828.9001	0.5688459	0.08637839	-0.5641456	903.1598	1542.5214	LOC_Os04g52310.1         	zinc transporter 1 precursor	transporter
gb_UNIGENE11777	5.9361143	0.00042019362	1.7810547	7433.1826	1273.3613	4.0542903	0.0006089975	1.3997756	6189.3174	1536.3835	LOC_Os03g29850.1         	zinc transporter 2 precursor	transporter
gb_UNIGENE16352	5.1283374	0.0076884027	1.6347815	1806.9225	343.0598	4.1676483	0.0015989929	1.427352	1881.8225	455.16513	LOC_Os07g12890.2         	ZIP zinc/iron transport family protein	transporter
gb_UNIGENE5919	3.897329	0.00018243608	1.3602915	4086.7224	1039.8333	3.5933487	0.0014575813	1.2790846	1444.1425	400.41882	LOC_Os01g70200.1         	secondary cell wall-related glycosyltransferase family 47	cell wall organization
gb_UNIGENE5920	3.8741388	0.00041235404	1.3543234	7527.045	1952.1525	3.3089154	0.0019926976	1.1966205	3382.2673	1010.13074	LOC_Os01g70200.1         	secondary cell wall-related glycosyltransferase family 47	cell wall organization
gb_UNIGENE11540	0.37574527	0.012356125	-0.97884387	5928.885	15906.699	0.49107435	0.0029396242	-0.7111597	5829.4053	11990.74	LOC_Os06g21250.1         	glycine-rich cell wall structural protein precursor	cell wall organization
gb_UNIGENE946	0.45562828	0.022098552	-0.786078	51725.176	107338.11	0.42000157	0.005700349	-0.86749685	30210.225	72943.445	LOC_Os02g37490.1         	glycine-rich cell wall structural protein precursor	cell wall organization
gb_UNIGENE11541	0.36626282	0.015391677	-1.0044041	6125.9126	16532.332	0.47541082	0.0032219128	-0.743576	5849.015	12338.106	LOC_Os06g21210.1         	glycine-rich cell wall structural protein precursor	cell wall organization
gb_UNIGENE3965	0.43706632	0.05162914	-0.82767034	97.7768	218.53703	0.40228838	0.0057406914	-0.9105861	60.04992	143.87473	LOC_Os01g70180.2         	secondary cell wall-related glycosyltransferase family 47	cell wall organization
gb_UNIGENE19670	0.4576285	0.09688051	-0.7816976	97.439224	194.92274	0.47190776	0.011352669	-0.75097173	40.995274	87.75838	LOC_Os02g04540.1         	vegetative cell wall protein gp1 precursor	cell wall organization
gb_UNIGENE13783	0.40594673	0.056071766	-0.9015333	270.11353	587.9971	0.2892548	0.01317326	-1.2404473	186.0885	638.6837	LOC_Os10g31540.1         	glycine-rich cell wall structural protein 2 precursor	cell wall organization
gb_UNIGENE23219	2.013091	0.07323113	0.69967145	33.894276	13.892227	3.234757	0.021718735	1.1739538	225.46597	54.401367	LOC_Os10g31730.1         	glycine-rich cell wall structural protein 2 precursor	cell wall organization
gb_UNIGENE9925	0.34234366	0.09475379	-1.0719402	232.40475	621.7605	0.44111434	0.02290248	-0.81845117	68.1343	142.49846	LOC_Os02g37490.1         	glycine-rich cell wall structural protein precursor	cell wall organization
gb_UNIGENE347	0.24740084	0.016098104	-1.3967454	3005.713	10777.99	0.42227593	0.0047650356	-0.8620963	2837.2324	6782.2896	LOC_Os01g52240.1         	chlorophyll a-b binding protein 2, chloroplast precursor	CAB
gb_UNIGENE951	0.27262488	0.019376766	-1.2996585	1581.1194	5088.631	0.30337408	0.019808032	-1.1927886	874.5685	2609.4868	LOC_Os01g52240.1         	chlorophyll a-b binding protein 2, chloroplast precursor	CAB
gb_UNIGENE960	0.30065846	0.033769786	-1.2017803	1118.5035	3243.3416	0.26678866	0.0062131006	-1.3212985	197.89523	708.2197	LOC_Os09g17740.1         	chlorophyll a-b binding protein 1, chloroplast precursor	CAB
gb_UNIGENE6	0.25717306	0.03434608	-1.358006	1064.9785	3893.9868	0.3115767	0.017116059	-1.1661098	575.8485	1840.0403	LOC_Os01g52240.1         	chlorophyll a-b binding protein 2, chloroplast precursor	CAB
gb_UNIGENE841	0.27183035	0.036285516	-1.3025771	7521.3076	22990.543	0.35512275	0.009142716	-1.0352918	3874.5273	10995.18	LOC_Os01g41710.1         	chlorophyll a-b binding protein 2, chloroplast precursor	CAB
gb_UNIGENE425	0.13635413	0.039601643	-1.9924998	1235.1777	7385.2256	0.120944716	0.014508339	-2.1124218	396.86127	3144.3835	LOC_Os01g52240.1         	chlorophyll a-b binding protein 2, chloroplast precursor	CAB
gb_UNIGENE422	0.3055893	0.036267888	-1.1855133	3603.2756	10487.892	0.579081	0.0684345	-0.5463129	334.33026	542.1819	LOC_Os01g41710.1         	chlorophyll a-b binding protein 2, chloroplast precursor	CAB
gb_UNIGENE1012	0.2247681	0.040032357	-1.492686	4945.478	17942.014	0.30293903	0.038245026	-1.1942238	1941.9401	6288.047	LOC_Os01g41710.1         	chlorophyll a-b binding protein 2, chloroplast precursor	CAB
gb_UNIGENE433	0.32632402	0.055898987	-1.1198645	21980.988	56275.03	0.3302566	0.013970099	-1.1078854	6539.983	18865.875	LOC_Os01g41710.1         	chlorophyll a-b binding protein 2, chloroplast precursor	CAB
gb_UNIGENE628	0.29003215	0.046081416	-1.2377635	10481.28	30292.344	0.27716148	0.014769552	-1.283155	1933.075	7216.115	LOC_Os01g41710.1         	chlorophyll a-b binding protein 2, chloroplast precursor	CAB
gb_UNIGENE1523	0.5456521	0.084887765	-0.60577375	12272.893	20205.87	0.4622783	0.016554017	-0.7715882	7291.3647	15359.387	LOC_Os04g38410.1         	chlorophyll a-b binding protein CP24, chloroplast precursor	CAB
gb_UNIGENE837	0.2922873	0.045287974	-1.2300181	9686.343	28418.29	0.28220677	0.017761124	-1.2651153	2985.4375	10697.045	LOC_Os01g41710.1         	chlorophyll a-b binding protein 2, chloroplast precursor	CAB
gb_UNIGENE617	0.3916096	0.09971034	-0.9374898	17302.873	37423.555	0.31787017	0.01850161	-1.1461122	7724.117	24634.566	LOC_Os01g52240.1         	chlorophyll a-b binding protein 2, chloroplast precursor	CAB
gb_UNIGENE432	0.3980224	0.09146587	-0.92124695	20709.809	43925.867	0.27575094	0.02112031	-1.2882572	6942.1426	24937.98	LOC_Os01g52240.1         	chlorophyll a-b binding protein 2, chloroplast precursor	CAB
gb_UNIGENE828	0.26061776	0.062572695	-1.3447005	10777.189	32523.361	0.22446671	0.022146998	-1.4940279	4032.84	17146.416	LOC_Os01g52240.1         	chlorophyll a-b binding protein 2, chloroplast precursor	CAB
gb_UNIGENE981	0.34196368	0.06511187	-1.0730507	1841.5615	4403.662	0.23692188	0.029712973	-1.4400249	583.2638	2215.652	LOC_Os01g52240.1         	chlorophyll a-b binding protein 2, chloroplast precursor	CAB
gb_UNIGENE418	0.1816135	0.06302359	-1.7058744	3133.705	13897.562	0.1179224	0.031693127	-2.1377285	746.49243	5564.6167	LOC_Os01g52240.1         	chlorophyll a-b binding protein 2, chloroplast precursor	CAB
gb_UNIGENE7013	0.47124484	0.04062405	-0.7523775	541.0353	1121.3676	0.546771	0.04234514	-0.6037252	974.3623	1707.6847	LOC_Os03g09060.1         	1,4-dihydroxy-2-naphthoate octaprenyltransferase	other
gb_UNIGENE19684	2.9093156	0.015840385	1.0679178	1058.362	332.95166	2.6897385	0.0007114828	0.989444	806.93945	301.75995	LOC_Os10g35950.1         	10-deacetylbaccatin III 10-O-acetyltransferase	other
gb_UNIGENE11701	2.15703	0.012416872	0.76873237	863.89825	423.61542	1.6835688	0.0046983776	0.52091587	821.54504	505.03107	LOC_Os01g08380.1         	10-deacetylbaccatin III 10-O-acetyltransferase	other
gb_UNIGENE2329	1.9666014	0.11106869	0.67630684	69.246796	43.542618	2.0072024	0.005318666	0.69674194	102.09602	52.58032	LOC_Os06g11210.1         	12-oxophytodienoate reductase 2	other
gb_UNIGENE2330	2.325141	0.0065678405	0.8437807	7190.6973	3153.1855	2.1653934	0.0026566414	0.7726021	7318.1753	3395.2944	LOC_Os06g11210.1         	12-oxophytodienoate reductase 2	other
gb_UNIGENE3288	2.6068106	0.017339384	0.95812744	1614.3601	633.8627	2.2872586	0.016282625	0.82735395	919.04224	402.9634	LOC_Os01g08860.1         	17.5 kDa class II heat shock protein	other
gb_UNIGENE3289	1.863481	0.029781885	0.6224463	3416.6074	1706.8842	2.0234559	0.006746631	0.7048069	2448.4624	1207.9913	LOC_Os01g08860.1         	17.5 kDa class II heat shock protein	other
gb_UNIGENE12084	5.17129	0.0070802607	1.6431222	887.54175	178.22574	3.9835758	0.009177964	1.3821799	616.4395	165.9557	LOC_Os09g24320.1         	2-oxo acid dehydrogenases acyltransferase family protein	other
gb_UNIGENE15147	2.423989	0.03200978	0.8854146	1171.5562	480.24228	1.739999	0.030008318	0.55388457	480.6605	270.77362	LOC_Os07g49520.1         	2-oxoglutarate dehydrogenase E1 component, mitochondrial precursor	other
gb_UNIGENE18058	4.086998	0.0010127054	1.4078107	62.44195	15.466051	2.4754078	0.0023694248	0.9064052	62.300976	25.700386	LOC_Os06g07220.1         	36.4 kDa proline-rich protein	other
gb_UNIGENE18311	0.33680364	0.03207004	-1.0882552	3105.615	8198.533	0.3208508	0.005046624	-1.1367791	2627.805	7857.4434	LOC_Os06g14810.1         	3-ketoacyl-CoA synthase	other
gb_UNIGENE6409	0.42163822	0.04600499	-0.86360765	91.64693	204.08185	0.49539298	0.004834137	-0.70240396	89.11183	186.3712	LOC_Os03g12030.1         	3-ketoacyl-CoA synthase	other
gb_UNIGENE5885	2.682765	0.00011791218	0.986848	2305.3274	866.9397	2.338195	0.0008687935	0.8493793	1407.7	601.9873	LOC_Os01g09010.1         	3-N-debenzoyl-2-deoxytaxol N-benzoyltransferase	other
gb_UNIGENE18583	0.49071494	0.03514702	-0.7118919	2137.631	3936.0479	0.47118896	0.031585157	-0.75249606	1519.8162	2974.481	LOC_Os04g36800.1         	3-oxoacyl-synthase I, chloroplast precursor	other
gb_UNIGENE8851	2.3740723	0.004352286	0.86460674	5527.6924	2348.2139	3.0759208	0.0047315476	1.1236043	4539.157	1497.6205	LOC_Os01g11860.1         	4-methyl-5-thiazole monophosphate biosynthesis protein	other
gb_UNIGENE6148	0.47913086	0.041372288	-0.7357815	8962.66	17183.535	0.60572356	0.020321721	-0.50133157	10505.113	16648.092	LOC_Os06g46930.1         	50S ribosomal protein L24, chloroplast precursor	other
gb_UNIGENE1424	2.5996618	0.06019163	0.95538133	23249.125	9147.953	2.2461627	0.017699908	0.80922323	21767.727	9294.104	LOC_Os12g42876.2         	5-methyltetrahydropteroyltriglutamate--homocysteine methyltransferase	other
gb_UNIGENE6490	2.0347836	0.0042909402	0.7103895	1128.5125	555.4505	1.8386308	0.00897206	0.6090212	1075.9413	577.7395	LOC_Os01g09570.1         	6-phosphofructokinase 2	other
gb_UNIGENE2598	2.336128	0.002890355	0.8484949	42715.35	18414.64	2.0749304	0.007276741	0.72992766	36960.15	17820.832	LOC_Os06g02144.2         	6-phosphogluconate dehydrogenase, decarboxylating	other
gb_UNIGENE20720	2.6057034	0.008532503	0.95770264	396.55725	153.23317	1.5637999	0.23520726	0.44711864	146.39383	94.655106	LOC_Os01g72900.1         	abscisic stress ripening protein 1	other
gb_UNIGENE8961	0.3331426	0.024301082	-1.0991846	5838.3125	15724.896	0.38999873	0.0068766116	-0.94161177	2759.3276	6694.1274	LOC_Os11g06720.1         	abscisic stress ripening protein 2	other
gb_UNIGENE4678	2.5102823	0.0028907964	0.9203952	16227.225	6493.4487	1.9955937	0.004605561	0.6909416	15923.325	7942.473	LOC_Os04g40150.1         	acetylglucosaminyltransferase/ transferase, transferring glycosyl groups	other
gb_UNIGENE21316	2.1196058	0.003927018	0.7512301	464.38074	221.18631	2.0969353	0.0041539385	0.74047685	268.4275	128.60057	LOC_Os06g08540.1         	actin binding protein	other
gb_UNIGENE12861	2.00971	0.0032057443	0.6979905	968.9755	475.79428	1.4806502	0.01874422	0.39248127	972.2265	651.07336	LOC_Os03g61930.1         	acyl-CoA binding protein	other
gb_UNIGENE9679	2.1483672	0.032366164	0.76470804	616.65076	295.81677	2.098609	0.030732308	0.7412747	314.5585	153.8319	LOC_Os09g34190.1         	acyl-coenzyme A thioesterase 9, mitochondrial precursor.	other
gb_UNIGENE23598	0.39192566	0.05188983	-0.9366831	70.59745	161.44229	0.3810536	0.021955363	-0.96481526	32.4657	83.97459	LOC_Os08g44840.1         	acyltransferase	other
gb_UNIGENE1791	3.013291	0.009363054	1.1030328	89969.125	31191.312	2.1344545	0.014875758	0.75821114	72099.52	35000.934	LOC_Os11g26850.2         	adenosylhomocysteinase	other
gb_UNIGENE1794	2.452748	0.03216736	0.89720905	4293.3325	1829.8026	1.6477108	0.021753443	0.4993869	2884.6526	1775.809	LOC_Os11g26850.2         	adenosylhomocysteinase	other
gb_UNIGENE12794	2.858356	0.00019363442	1.0502466	7162.55	2507.5913	1.7623332	0.0019730455	0.5666386	3440.8076	1944.7384	LOC_Os03g31679.1         	adhesive/proline-rich protein	other
gb_UNIGENE1536	2.012434	0.02306297	0.69934493	1931.8125	974.9065	1.6309695	0.02360901	0.48917466	833.9442	507.96417	LOC_Os02g48720.2         	ADP,ATP carrier protein, mitochondrial precursor	other
gb_UNIGENE9863	2.5628996	0.0021908318	0.9411393	4231.4526	1678.9152	2.3178604	0.0040922295	0.84064454	2830.3474	1264.0087	LOC_Os05g37660.1         	AER	other
gb_UNIGENE20247	3.5361354	0.00512526	1.2630345	772.78076	212.61832	4.8449717	0.010221986	1.5779414	447.754	86.73584	LOC_Os09g37200.1         	agmatine coumaroyltransferase	other
gb_UNIGENE15413	3.297143	0.02421296	1.1930563	176.51749	60.689606	12.527168	0.0021263345	2.5278997	356.253	26.347467	LOC_Os03g08720.1         	agmatine coumaroyltransferase	other
gb_UNIGENE9093	2.203836	0.0034394215	0.79019946	461.50177	209.72911	2.0838435	0.007847225	0.73421407	320.16675	159.4324	LOC_Os08g41290.1         	AIR12	other
gb_UNIGENE7751	3.2847261	0.00033464728	1.1892833	2533.5774	769.24445	3.3733077	0.00018743804	1.2158937	2274.5176	675.4258	LOC_Os01g03030.1         	alanyl-tRNA synthetase	other
gb_UNIGENE5648	4.3250074	0.00024964317	1.4644139	7633.6553	1778.9396	3.8944094	0.0008441737	1.359542	5659.4746	1463.8031	LOC_Os04g38540.1         	aldose 1-epimerase	other
gb_UNIGENE1642	2.0668154	0.0020841106	0.72600895	7946.9575	3867.4043	1.6696005	0.0029179452	0.5125844	6421.468	3827.0696	LOC_Os03g40270.1         	alpha-1,4-glucan-protein synthase	other
gb_UNIGENE1634	2.7225664	0.0137876915	1.001575	36.446877	13.262829	1.8865081	0.007819058	0.63472754	78.2018	40.49734	LOC_Os03g40270.1         	alpha-1,4-glucan-protein synthase	other
gb_UNIGENE4058	2.5731819	0.005004771	0.9451432	132.94049	53.13065						LOC_Os04g44470.1         	alpha-amylase/subtilisin inhibitor	other
gb_UNIGENE2787	7.8577986	0.009638851	2.0615065	6152.585	783.536	18.584822	0.00037692158	2.9223452	11523.234	618.52405	LOC_Os12g43490.1         	alpha-amylase/trypsin inhibitor	other
gb_UNIGENE2790	7.1760683	0.013151226	1.9707516	4245.02	610.705	17.880682	0.0006682594	2.8837209	3220.55	180.5109	LOC_Os12g43490.1         	alpha-amylase/trypsin inhibitor	other
gb_UNIGENE3674	0.35486782	0.021809192	-1.0360099	6624.097	16335.104	0.35893393	0.012978333	-1.024617	4939.895	12729.84	LOC_Os05g39990.1         	alpha-expansin 1 precursor	other
gb_UNIGENE3675	0.14647949	0.013605213	-1.9208698	531.6611	2383.584	0.23776938	0.004788402	-1.436454	574.5402	2215.5542	LOC_Os01g60770.1         	alpha-expansin 10 precursor	other
gb_UNIGENE3023	4.6868086	0.00041163	1.5447519	68487.625	14741.393	3.794043	0.00058825326	1.3334322	54481.25	14558.5625	LOC_Os06g06290.3         	alpha-L-fucosidase 2 precursor	other
gb_UNIGENE14937	2.1782663	0.0058073434	0.7785293	365.08652	166.23465	2.0113478	0.005032722	0.698805	281.07175	143.47961	LOC_Os10g06710.1         	amidase	other
gb_UNIGENE3787	0.5202708	0.056102704	-0.6534058	3896.5625	6683.698	0.4709674	0.031913344	-0.7529664	2707.97	5155.0996	LOC_Os05g02060.1         	amino acid selective channel protein	other
gb_UNIGENE11240	2.8114583	0.01407315	1.0337033	3653.065	1401.5593	2.341813	0.0051775114	0.85092545	805.15625	348.24692	LOC_Os01g42234.1         	amino-acid permease C584.13	other
gb_UNIGENE3350	2.0213635	0.00035473026	0.7037723	312.90326	155.65565	1.6130377	0.0059857047	0.4781192	196.73724	124.15381	LOC_Os06g11800.1         	annexin-like protein RJ4	other
gb_UNIGENE9200	0.53069025	0.07349938	-0.63357675	2164.1377	3792.7202	0.49163583	0.021463254	-0.710017	1527.2123	2941.6016	LOC_Os05g31750.1         	annexin-like protein RJ4	other
gb_UNIGENE21904	4.4231915	0.0021299084	1.4868615	467.57202	102.52402	3.9864836	0.003116669	1.3829095	306.643	75.21246	LOC_Os03g60260.1         	ANT1	other
gb_UNIGENE12524	4.7002473	0.0016594214	1.5476152	356.059	78.2739	1.5028259	0.015945982	0.4073472	341.86597	228.50339	LOC_Os02g56010.1         	anthocyanidin 3-O-glucosyltransferase, putative	other
gb_UNIGENE25368	6.4261265	0.0047928966	1.860372	866.71576	142.32921	7.917406	0.0007092009	2.0690637	171.80525	21.97315	LOC_Os01g53350.1         	anthocyanidin 5,3-O-glucosyltransferase	other
gb_UNIGENE18002	6.873887	0.0019311059	1.9277297	72.61052	10	8.1118765	0.0020088453	2.0933292	93.762474	11.380057	LOC_Os05g48010.1         	anthocyanin regulatory C1 protein	other
gb_UNIGENE6406	3.874802	0.0019937563	1.8799013	1683.0425	271.81528	7.333879	0.0002456746	1.9925046	1215.0027	159.96375	LOC_Os08g23150.1         	indole-3-glycerol phosphate synthase, chloroplast precursor	other
gb_UNIGENE16901	0.34283245	0.006561317	-1.0705135	2046.7224	5812.662	0.34909174	0.00019682455	-1.0524205	393.98276	1118.0779	LOC_Os10g36924.1         	aquaporin NIP5.1	other
gb_UNIGENE8087	0.41606316	0.011910672	-0.8769182	2189.9475	5527.5723	0.7570352	0.35618338	-0.27834556	2138.7925	3003.4348	LOC_Os02g04840.1         	ARA1	other
gb_UNIGENE16526	2.3097348	0.02147675	0.8371327	18107.326	7963.05	2.5872219	0.027582154	0.95058465	15824.225	6037.5024	LOC_Os06g04070.1         	arginine decarboxylase	other
gb_UNIGENE6113	2.7336984	0.0029894512	1.0056554	3372.0425	1227.4155	2.1707265	0.0003634035	0.77506196	2842.79	1310.6802	LOC_Os03g19280.1         	argininosuccinate lyase	other
gb_UNIGENE13341	2.6406314	0.0009739582	0.9710181	76.931244	28.677423	2.8628814	0.019625127	1.0518286	45.94845	17.34307	LOC_Os02g37080.1         	ASC1-like protein 1	other
gb_UNIGENE5164	1.9714031	0.083436765	0.6787455	57.5876	26.268696	2.268023	0.027801402	0.8189086	29.990826	14.221653	LOC_Os03g18130.1         	asparagine synthetase	other
gb_UNIGENE6380	2.6201746	0.021159427	0.963241	1178.1093	445.76303	3.0523942	0.0051380317	1.1159263	1034.0157	331.4874	LOC_Os11g08100.1         	aspartic proteinase Asp1 precursor	other
gb_UNIGENE24409	1.7027194	0.03381969	0.5322266	2166.27	1248.6289	2.0628223	0.01145386	0.7240751	1776.9425	853.0901	LOC_Os06g15760.1         	aspartic proteinase Asp1 precursor	other
gb_UNIGENE9000	0.40379667	0.029764935	-0.9068438	1434.5317	3121.2637	0.39369938	0.0048110755	-0.93216765	1147.0007	2774.6685	LOC_Os02g48900.2         	aspartic proteinase nepenthesin-1 precursor	other
gb_UNIGENE6013	6.761898	0.0010706842	1.9113036	473.11975	71.709015	3.251871	0.01712876	1.1792306	314.098	99.2827	LOC_Os02g48870.1         	aspartic proteinase nepenthesin-2 precursor	other
gb_UNIGENE13091	4.141209	0.0024497234	1.4209878	11090.207	2639.832	3.001368	0.0049930303	1.0990682	9541.476	3227.1738	LOC_Os06g50390.1         	aspartic-type endopeptidase/ pepsin A	other
gb_UNIGENE21100	2.02974	0.0050620786	0.7079078	471.4265	234.59912	1.6168281	0.008249938	0.48046628	465.23102	286.813	LOC_Os08g41190.1         	ATBPM1, putative	other
gb_UNIGENE3057	0.44962707	0.08416831	-0.7993368	1497.1964	3166.9863	0.41852105	0.026140045	-0.87102807	969.01404	2145.1147	LOC_Os04g32030.1         	ATFP3	other
gb_UNIGENE15046	3.5656478	0.00034464817	1.2713457	439.81952	123.5544	2.7803693	0.010073075	1.0225837	519.4405	192.83977	LOC_Os03g02240.1         	AT-GTL1	other
gb_UNIGENE13905	3.3607721	0.00045932952	1.2121707	6436.145	1908.3727	2.6046567	0.0013713492	0.95730084	2488.4375	952.6272	LOC_Os03g62180.3         	ATP binding protein	other
gb_UNIGENE18748	2.3561583	0.0021129062	0.8570324	1625.145	685.99414	2.144892	0.001787549	0.76308924	1496.475	702.1229	LOC_Os05g39410.3         	ATP binding protein	other
gb_UNIGENE17992	0.49542284	0.0017378766	-0.70234364	462.75226	925.81335	0.61090446	0.007936253	-0.49281475	508.89224	833.5757	LOC_Os06g38970.1         	ATP binding protein	other
gb_UNIGENE20838	2.2702858	0.0038351242	0.8199057	377.49277	165.32214	1.511851	0.039352633	0.4133347	409.5845	266.92313	LOC_Os01g26390.1         	ATP binding protein	other
gb_UNIGENE22199	2.3207037	0.021938525	0.8418704	190.8745	84.21575	1.740557	0.0038364527	0.5542051	456.148	261.61783	LOC_Os04g23700.1         	ATP binding protein	other
gb_UNIGENE2568	2.0383086	0.025360342	0.7121204	59658.574	30940.055	1.605167	0.0018886265	0.4732278	64033.08	40446.305	LOC_Os03g37120.1         	ATP binding protein	other
gb_UNIGENE18208	0.45645186	0.025839685	-0.784272	76.64037	158.20311	0.44395489	0.058468807	-0.81203234	29.960442	58.281612	LOC_Os01g12390.1         	ATP binding protein	other
gb_UNIGENE19731	1.6274903	0.021622956	0.48703915	27.394001	17.327888	2.02351	0.022112442	0.70483357	30.779425	15.354799	LOC_Os04g56010.1         	ATP binding protein, putative	other
gb_UNIGENE21659	1.9072375	0.05647793	0.6456559	1039.5592	570.29694	2.0172777	0.03443747	0.70174897	756.0885	372.61206	LOC_Os11g37700.1         	ATPase, coupled to transmembrane movement of substances	other
gb_UNIGENE23105	0.49991354	0.000021455442	-0.6933201	515.64	1030.3252	0.505499	0.0009577735	-0.6822092	840.42474	1661.014	LOC_Os03g02550.1         	ATPP2-A13	other
gb_UNIGENE16892	5.781437	0.00072597025	1.7546523	613.23724	105.35574	6.179745	0.0017923139	1.821277	548.44073	90.35792	LOC_Os03g08880.1         	ATPUP3	other
gb_UNIGENE3375	0.47572502	0.024627162	-0.7429153	2431.3025	5204.0156	0.71612513	0.13216905	-0.33390033	2538.57	3659.8242	LOC_Os09g23350.1         	ATTPS6	other
gb_UNIGENE8605	2.999499	0.009323916	1.0984453	4966.6777	1666.1415	2.3773286	0.012268671	0.8659774	5347.5947	2211.4944	LOC_Os07g17330.1         	B12D protein	other
gb_UNIGENE25923	1.7586552	0.3577864	0.56454945	40.4892	22.34739	2.204237	0.022420047	0.7903814	27.61875	12.386237	LOC_Os03g50140.1         	basic blue protein precursor	other
gb_UNIGENE26053	12.9639435	0.0016515743	2.562172	1598.8875	129.32275	16.16182	0.000035867084	2.7826517	447.6765	27.494867	LOC_Os06g22440.1         	benzoate carboxyl methyltransferase	other
gb_UNIGENE24534	0.37693268	0.006262975	-0.9756887	94.2628	244.21614	0.4320064	0.024028448	-0.8393149	132.03012	286.45355	LOC_Os06g20630.1         	benzoate carboxyl methyltransferase, putative	other
gb_UNIGENE11383	0.4569262	0.030789549	-0.7832334	23715.902	47178.496	0.40670064	0.005731361	-0.8996779	13101.607	30890.562	LOC_Os09g39920.1         	beta-1,4-mannan synthase	other
gb_UNIGENE10435	0.5661815	0.17637889	-0.56884056	185.61398	296.84106	0.39957508	0.028318616	-0.9173536	91.62881	208.37003	LOC_Os03g17850.1         	beta3-glucuronyltransferase	other
gb_UNIGENE13674	0.4508119	0.04346248	-0.7967051	6136.9355	12410.111	0.49537662	0.022457227	-0.702437	5642.512	11658.644	LOC_Os11g19210.1         	beta-D-xylosidase	other
gb_UNIGENE7394	0.16450734	0.030873159	-1.8048	1883.2872	7631.4497	0.30542046	0.0007024764	-1.1860659	1749.3647	5514.4033	LOC_Os10g40700.1         	beta-expansin 1a precursor	other
gb_UNIGENE2877	0.13680583	0.03348448	-1.9891926	654.0044	2833.5588	0.14675996	0.0043614623	-1.918957	215.75314	1190.8141	LOC_Os10g40720.1         	beta-expansin 1a precursor	other
gb_UNIGENE2967	0.44791812	0.029052967	-0.8031448	115.90018	244.55014	0.45012504	0.006137722	-0.7982299	101.30183	238.64073	LOC_Os02g44108.1         	beta-expansin 4 precursor	other
gb_UNIGENE23526	0.37965623	0.0069430205	-0.9684891	534.0235	1339.4348	0.53424305	0.0016467646	-0.6269044	502.54547	950.56824	LOC_Os04g45290.1         	beta-fructofuranosidase 1 precursor	other
gb_UNIGENE6539	4.1844907	0.00037453737	1.431385	16786.799	3918.438	3.5911267	0.0024088067	1.278466	12386.475	3519.4075	LOC_Os02g33110.1         	beta-fructofuranosidase, insoluble isoenzyme 1 precursor	other
gb_UNIGENE13144	2.745254	0.0000672278	1.0098736	269.49527	97.99902	2.282125	0.000038191687	0.825107	195.8315	85.63989	LOC_Os11g05880.1         	protein binding protein	other
gb_UNIGENE7773	5.1736727	0.0009908342	1.6435828	2612.2449	496.11154	4.470248	0.0029645714	1.4974439	2033.0425	456.42896	LOC_Os01g61990.1         	protein binding protein	other
gb_UNIGENE11918	8.314801	0.0076317913	2.1180372	151.99092	18.568995	7.4992065	0.0016378489	2.0147972	79.4064	10.100733	LOC_Os02g45780.1         	protein binding protein	other
gb_UNIGENE16581	2.074725	0.042856455	0.72982854	66.426796	32.98927	2.085929	0.000803144	0.73521423	53.3647	25.580624	LOC_Os12g40790.1         	protein binding protein	other
gb_UNIGENE8760	0.5609952	0.084816605	-0.5780429	249.60199	422.67508	0.33885908	0.017306956	-1.082171	81.93825	231.8906	LOC_Os08g43270.1         	protein binding protein	other
gb_UNIGENE3208	7.905787	0.006282923	2.067595	634.652	86.206635	3.5380127	0.0068743606	1.2635652	898.93274	241.64394	LOC_Os09g39940.1         	blue copper protein precursor	other
gb_UNIGENE3209	6.0475106	0.01017893	1.7996467	1382.593	240.19514	5.873841	0.0030539876	1.7705088	1182.7762	182.91412	LOC_Os09g39940.1         	blue copper protein precursor	other
gb_UNIGENE18690	0.2708386	0.020637693	-1.3062322	149.43222	493.09088	0.39365256	0.024870679	-0.9322866	141.4073	338.60236	LOC_Os03g55120.1         	blue copper protein precursor	other
gb_UNIGENE3211	4.8960094	0.07226322	1.5884205	441.20126	181.7228	7.145636	0.008856427	1.9665018	326.213	43.58186	LOC_Os09g39940.1         	blue copper protein precursor	other
gb_UNIGENE17750	2.7728794	0.005353593	1.0198863	3891.76	1399.8959	1.7680213	0.0014934107	0.56986105	1691.9425	952.2898	LOC_Os03g57740.1         	blue copper protein precursor, putative	other
gb_UNIGENE10717	1.5241148	0.16787694	0.42141384	553.12897	347.31097	2.1826468	0.0004696206	0.7805382	533.19824	241.94788	LOC_Os01g03680.1         	Bowman-Birk type bran trypsin inhibitor precursor	other
gb_UNIGENE17082	7.7422743	0.00080701185	2.0466955	29541.477	3823.4236	8.470193	0.0012686356	2.1365533	13560.175	1634.2568	LOC_Os03g60840.1         	Bowman-Birk type trypsin inhibitor	other
gb_UNIGENE2241	6.1417127	0.009574349	1.8151037	33486.35	5291.5967	9.578342	0.00094428385	2.2595046	65412.3	6927.71	LOC_Os01g04040.1         	Bowman-Birk type wound-induced proteinase inhibitor WIP1 precursor, putative	other
gb_UNIGENE2243	3.1764526	0.12894954	1.155765	160.86493	84.13513	4.1770577	0.00881482	1.4296072	47.196022	10.602237	LOC_Os01g04040.1         	Bowman-Birk type wound-induced proteinase inhibitor WIP1 precursor, putative	other
gb_UNIGENE4750	1.6919333	0.17678706	0.5258718	1259.4465	785.8154	2.0518963	0.02041021	0.71876436	1197.8325	594.6511	LOC_Os01g04040.1         	Bowman-Birk type wound-induced proteinase inhibitor WIP1 precursor, putative	other
gb_UNIGENE3360	2.1727562	0.00080581196	0.77599645	13479.25	6146.882	1.7146676	0.005385821	0.5392192	13446.625	7795.0566	LOC_Os01g73250.1         	bundle sheath cell specific protein 1	other
gb_UNIGENE25984	0.38269898	0.02297247	-0.96050656	261.88376	626.2044	0.5262713	0.114125855	-0.64193845	37.878376	81.32663	LOC_Os07g41850.1         	C1-like domain containing protein	other
gb_UNIGENE14263	2.1774669	0.030887866	0.77816224	935.58704	444.2464	1.4962108	0.02415203	0.40293577	486.39774	321.15485	LOC_Os07g41850.1         	C1-like domain containing protein	other
gb_UNIGENE112	2.2791038	0.0016199007	0.8237823	41670.547	18286.61	1.7533585	0.0074317064	0.5615331	33005.324	18594.52	LOC_Os09g39770.1         	C2 domain containing protein	other
gb_UNIGENE21914	3.814239	0.0011370276	1.3387412	3245.8525	849.4636	2.4756382	0.0013581043	0.9064982	2937.7524	1195.3099	LOC_Os03g21380.2         	calcium-binding protein CAST	other
gb_UNIGENE4593	2.0579438	0.0009145467	0.72170734	5018.415	2424.7117	1.7290891	0.0033531038	0.5475948	4656.7397	2691.495	LOC_Os05g50810.1         	calcium-dependent protein kinase, isoform 2	other
gb_UNIGENE16998	3.5150714	0.000719875	1.2570598	2105.0676	602.9942	2.719459	0.0015290974	1.000433	1524.599	589.47046	LOC_Os03g57450.1         	calcium-dependent protein kinase, isoform AK1	other
gb_UNIGENE14715	6.0264196	0.0005658275	1.7961531	10683.883	1759.2328	3.0490136	0.0018483454	1.1148181	8827.28	2927.7068	LOC_Os03g10640.1         	calcium-transporting ATPase 2, plasma membrane-type	other
gb_UNIGENE14714	6.1882067	0.005013322	1.8226453	1846.345	290.69974	3.5932927	0.0046166577	1.279069	1632.7625	456.4358	LOC_Os03g10640.1         	calcium-transporting ATPase 2, plasma membrane-type	other
gb_UNIGENE14075	6.448681	0.00060548086	1.8638756	1295.9495	198.06116	5.0884013	0.003028129	1.6269637	1420.5371	294.40707	LOC_Os05g41580.1         	calcium-transporting ATPase 4, plasma membrane-type	other
gb_UNIGENE9475	2.6027088	0.0011244287	0.95655274	1555.6699	601.9967	1.9832135	0.0034363281	0.68471855	1360.3398	692.6976	LOC_Os08g40530.2         	calcium-transporting ATPase 9, plasma membrane-type	other
gb_UNIGENE1321	2.4231937	0.000036943573	0.88508636	3137.405	1297.7314	2.0945635	0.00033627977	0.73934513	1934.23	924.07825	LOC_Os07g48780.1         	calmodulin	other
gb_UNIGENE8468	3.0018375	0.00018021633	1.0992246	3469.16	1153.7297	2.672138	0.0010021919	0.9828789	4378.6475	1632.2587	LOC_Os01g38980.2         	calmodulin binding protein	other
gb_UNIGENE20239	5.484349	0.001095968	1.7018983	2298.8926	422.67676	5.914034	0.0014963668	1.7773281	1736.9575	290.81464	LOC_Os07g42740.1         	calmodulin binding protein	other
gb_UNIGENE14915	6.487363	0.0037371311	1.8698561	104.1532	18.69431	12.401374	0.0015943794	2.5178072	232.53725	19.59983	LOC_Os12g36110.1         	calmodulin binding protein	other
gb_UNIGENE15493	3.2021778	0.0028101837	1.1638311	2138.2275	679.5161	2.6878965	0.0046819504	0.9887589	1774.8275	650.38696	LOC_Os06g14490.2         	calmodulin-binding heat-shock protein	other
gb_UNIGENE5761	3.269627	0.0017236377	1.1846759	13194.75	4129.049	2.0398705	0.003208375	0.7128863	10489.5	5207.625	LOC_Os01g72080.1         	calmodulin-like protein 1	other
gb_UNIGENE17335	4.7644224	0.0018525484	1.5611763	656.51624	137.60837	2.5583189	0.00031758807	0.93935037	321.7635	126.70226	LOC_Os12g04360.1         	calmodulin-like protein 1	other
gb_UNIGENE10407	8.406682	0.0017949942	2.129027	1975.96	245.87576	7.140601	0.000026720061	1.965797	7331.1074	1036.1445	LOC_Os09g24580.1         	calmodulin-like protein	other
gb_UNIGENE5941	3.3872643	0.0000057061825	1.2200226	1741.1649	512.8692	3.109216	0.0014477836	1.1343706	797.33527	263.8388	LOC_Os01g04330.1         	calmodulin-related protein 2, touch-induced	other
gb_UNIGENE1560	2.4400392	0.0005464049	0.8920141	562.334	233.22827	1.7549398	0.00423286	0.56243455	540.26825	311.6105	LOC_Os04g32950.1         	calnexin homolog precursor	other
gb_UNIGENE1903	5.120874	0.003175377	1.6333251	1422.255	277.46814	3.0106227	0.0018354444	1.102147	879.15625	293.41968	LOC_Os07g14270.3         	calreticulin precursor	other
gb_UNIGENE2747	4.0286403	0.001430788	1.3934289	88720.2	22264.807	2.5711946	0.014921746	0.9443706	68467.35	28198.707	LOC_Os01g67054.1         	calreticulin-3 precursor	other
gb_UNIGENE11276	2.113319	0.000244261	0.7482596	1317.225	623.0929	1.438462	0.019048838	0.3635745	636.31177	437.11102	LOC_Os03g52760.1         	carbohydrate kinase, PfkB	other
gb_UNIGENE17756	0.4069875	0.026274122	-0.8989728	4271.8027	9930.593	0.47296432	0.021126727	-0.7487353	3723.815	7639.277	LOC_Os04g33660.2         	carbonic anhydrase precursor	other
gb_UNIGENE1899	2.1744866	0.0070328377	0.7767926	1366.2125	613.7395	1.6575582	0.019577643	0.5053456	794.19604	479.63757	LOC_Os07g44070.1         	carboxylic ester hydrolase	other
gb_UNIGENE11693	0.49502137	0.020689584	-0.7031543	418.20374	793.95605	0.5514201	0.015261255	-0.5952583	372.744	677.2051	LOC_Os01g66850.4         	carboxylic ester hydrolase	other
gb_UNIGENE9967	0.559724	0.10887294	-0.5803115	724.0812	1196.4879	0.3990355	0.034375302	-0.91870487	253.03851	603.96625	LOC_Os01g66830.2         	carboxylic ester hydrolase	other
gb_UNIGENE5274	0.34116492	0.028855758	-1.0753893	274.293	694.77747	0.2679505	0.0030484004	-1.316953	101.104324	352.26334	LOC_Os03g59240.1         	carboxyl-terminal peptidase	other
gb_UNIGENE2169	2.3907645	0.006134336	0.87161314	8502.545	3682.5166	2.1642787	0.011640822	0.77208716	5766.4624	2674.8	LOC_Os03g02514.2         	catalytic/ hydrolase	other
gb_UNIGENE20097	0.41802546	0.017567182	-0.87221295	3151.1726	6937.889	0.46526182	0.008177246	-0.76515496	2064.0076	4411.3135	LOC_Os12g02589.3         	catalytic/ hydrolase	other
gb_UNIGENE23016	6.451514	0.0014195365	1.8643148	3664.0598	584.8847	4.313868	0.00003846973	1.4618349	3368.9224	784.1403	LOC_Os03g10950.1         	catalytic/ protein phosphatase type 2C	other
gb_UNIGENE20759	2.2081237	0.0065527987	0.7921431	1424.3625	649.59064	2.4975562	0.0017204536	0.91531277	1099.4296	434.5197	LOC_Os03g60650.1         	catalytic/ protein phosphatase type 2C	other
gb_UNIGENE11985	0.45909405	0.0039855638	-0.7785002	2299.01	4871.5264	0.49151123	0.022961052	-0.7102705	2714.2275	5455.631	LOC_Os02g05470.1         	CCT motif family protein	other
gb_UNIGENE15708	2.4779434	0.011278106	0.907429	2438.0325	1057.6426	2.0743835	0.005231745	0.729664	1146.6904	547.0083	LOC_Os06g48250.1         	cell Division Protein AAA ATPase family	other
gb_UNIGENE9267	3.6890314	0.007832834	1.3053639	2656.9075	665.0039	2.2188394	0.0028395604	0.79698426	2262.735	1027.4081	LOC_Os10g22570.1         	cellulase containing protein	other
gb_UNIGENE9266	5.013427	0.016121097	1.6121197	578.42975	101.84508	2.3735814	0.0059785713	0.8643999	517.26	216.78532	LOC_Os10g22570.1         	cellulase containing protein	other
gb_UNIGENE7133	0.49164477	0.0059506777	-0.70999885	398.376	813.60754	0.49191093	0.016816294	-0.70945764	257.652	518.0322	LOC_Os05g07680.2         	CENP-E like kinetochore protein	other
gb_UNIGENE11385	2.341142	0.012490502	0.85063887	133.679	58.91557	1.2590281	0.18182337	0.23034006	76.08308	61.046753	LOC_Os11g35090.1         	centromeric protein E	other
gb_UNIGENE14570	0.15301193	0.014833101	-1.8772393	75.304794	417.47217	0.16641347	0.000395843	-1.7932798	25.105131	154.57448	LOC_Os10g33250.2         	CER1	other
gb_UNIGENE15116	0.3763144	0.058548994	-0.9773303	722.6802	1468.6191	0.19375284	0.021482434	-1.6411719	25.072363	112.485	LOC_Os01g54620.1         	CESA4 - cellulose synthase	other
gb_UNIGENE20165	0.40196878	0.058028456	-0.9113808	1595.3971	3435.3206	0.23892102	0.027897378	-1.4316223	533.972	1961.4818	LOC_Os09g25490.1         	CESA9 - cellulose synthase	other
gb_UNIGENE26163	0.71738684	0.25775415	-0.3321401	33.755077	43.14931	0.4920679	0.014097813	-0.7091386	14.330151	30.028584	LOC_Os03g04970.1         	chaperonin CPN60-1, mitochondrial precursor	other
gb_UNIGENE16898	3.1515827	0.022375327	1.1479048	73.38728	23.054117	6.0542784	0.00058634876	1.8007652	147.35448	23.489935	LOC_Os09g32570.1         	chloroplastic quinone-oxidoreductase	other
gb_UNIGENE21197	2.9833314	0.00062150613	1.0930406	2619.4475	879.3325	3.4970767	0.0005056076	1.2519274	2305.04	659.32043	LOC_Os03g56260.1         	chromosome-associated kinesin KIF4A	other
gb_UNIGENE6637	0.43795708	0.012473019	-0.82563436	786.5643	1734.3727	0.4782992	0.01608437	-0.7375188	691.7555	1476.2366	LOC_Os09g02650.1         	chromosome-associated kinesin KLP1	other
gb_UNIGENE24308	2.5118823	0.000064011234	0.9210324	319.00348	126.8847	1.9753506	0.018491618	0.6807459	237.25327	123.59308	LOC_Os04g46980.1         	cis-zeatin O-glucosyltransferase 1	other
gb_UNIGENE9541	2.2957108	0.0030327456	0.83104247	6080.315	2638.6584	1.7468807	0.000643265	0.5578317	4627.665	2653.4077	LOC_Os02g21250.1         	coatomer subunit zeta-1	other
gb_UNIGENE6773	4.567882	0.003686914	1.5190496	383.813	82.99664	4.8502755	0.010308154	1.5790355	269.45175	59.39144	LOC_Os03g18910.1         	COBRA-like protein 7 precursor	other
gb_UNIGENE3112	0.3084153	0.010038859	-1.176308	10161.333	33117.215	0.5243087	0.0146320835	-0.64567465	9605.383	18960.252	LOC_Os03g55850.1         	cold acclimation protein COR413-PM1	other
gb_UNIGENE19799	3.8463337	0.00038825817	1.3471204	270.70377	69.38385	3.3035016	0.0021059334	1.194983	169.14075	51.729603	LOC_Os07g40470.1         	conserved hypothetical protein	other
gb_UNIGENE6014	3.1943035	0.0007795366	1.1613691	859.7467	277.03314	2.5193954	0.009319107	0.924019	460.2865	184.9387	LOC_Os03g08940.1         	conserved hypothetical protein	other
gb_UNIGENE6015	3.1784408	0.0034741408	1.1563908	1306.9125	429.29556	2.501973	0.011600872	0.91707957	709.54175	287.30658	LOC_Os03g08940.1         	conserved hypothetical protein	other
gb_UNIGENE12481	3.8285282	0.0058649676	1.3424804	583.5685	149.32071	7.5650563	0.00091869914	2.0235398	1103.6438	143.8498	LOC_Os09g27410.1         	conserved hypothetical protein	other
gb_UNIGENE16680	6.801343	0.02988134	1.9171201	235.0925	42.06111	9.670019	0.00045781813	2.2690303	127.40663	13.192495	LOC_Os05g42340.1         	conserved hypothetical protein	other
gb_UNIGENE26418	0.47541174	0.001959222	-0.743574	242.26825	512.44586	0.9307558	0.535881	-0.07175833	89.0963	96.34709	LOC_Os10g04540.1         	conserved hypothetical protein	other
gb_UNIGENE13517	3.9258673	0.0039300625	1.3675873	617.4938	163.95401	1.9493454	0.0033225054	0.6674936	387.74802	200.58728	LOC_Os02g17400.1         	conserved hypothetical protein	other
gb_UNIGENE19832	0.38091457	0.0040480816	-0.96518016	203.79053	509.27203	0.542052	0.0005954313	-0.61239344	238.99176	430.03043	LOC_Os01g73090.1         	conserved hypothetical protein	other
gb_UNIGENE11103	2.9299407	0.028433248	1.0749822	4260.02	1514.9515	2.8438706	0.025188658	1.045166	4553.625	1587.338	LOC_Os06g40650.2         	copine-1	other
gb_UNIGENE7538	3.6373243	0.0030379556	1.2912483	2335.8574	637.8641	3.7623978	0.009788236	1.3250564	2174.3374	624.0892	LOC_Os01g68060.1         	copine-6	other
gb_UNIGENE6917	0.4643911	0.010287948	-0.76702815	588.625	1212.2206	0.55162424	0.016478065	-0.5948882	674.83075	1208.8477	LOC_Os07g43040.1         	copper-transporting ATPase PAA1	other
gb_UNIGENE1179	9.729133	0.00067995896	2.2751248	18282.895	1828.2421						LOC_Os01g05970.1         	core histone H2A/H2B/H3/H4 family protein	other
gb_UNIGENE1147	0.42408428	0.06646863	-0.8578231	242.92625	566.73016	0.46262202	0.005897234	-0.77084494	263.398	566.8998	LOC_Os01g05970.1         	core histone H2A/H2B/H3/H4 family protein	other
gb_UNIGENE1138	0.5125966	0.0724291	-0.66826606	195.94691	396.85217	0.48448834	0.0098118	-0.72466195	192.24825	381.2694	LOC_Os01g05970.1         	core histone H2A/H2B/H3/H4 family protein	other
gb_UNIGENE5808	0.22111449	0.07382282	-1.5090747	476.876	2088.5796	0.1898085	0.0125975115	-1.6617396	87.33293	584.90405	LOC_Os04g46830.1         	cortical cell-delineating protein precursor	other
gb_UNIGENE17964	2.2665215	0.0007976006	0.8182463	928.6187	409.31863	1.9883487	0.0040908796	0.6873045	1216.4208	602.57404	LOC_Os03g18560.1         	cp protein	other
gb_UNIGENE22016	2.3287654	0.00043863594	0.8453383	161.47876	69.182976						LOC_Os07g35690.2         	CRK6	other
gb_UNIGENE17916	6.2487683	0.008434188	1.8323843	5012.5376	817.8645	3.0869975	0.017020673	1.1271989	5207.0474	1802.904	LOC_Os08g28240.1         	crocetin dialdehyde	other
gb_UNIGENE8486	0.5093133	0.07823454	-0.6746919	312.154	611.5203	0.45253575	0.008860677	-0.7928885	213.9775	452.3423	LOC_Os05g43530.1         	CSLC7 - cellulose synthase-like family C	other
gb_UNIGENE8067	4.131378	0.002926933	1.418611	4764.8125	1118.0359	3.861433	0.007422783	1.3510383	2309.4976	613.34076	LOC_Os09g30130.1         	CSLE6 - cellulose synthase-like family E	other
gb_UNIGENE5706	0.36061445	0.019332843	-1.0199459	332.7885	866.3527	0.37682992	0.016675768	-0.9759613	144.696	386.62015	LOC_Os08g06380.1         	CSLF6 - cellulose synthase-like family F; beta1,3;1,4 glucan synthase	other
gb_UNIGENE13847	0.30941883	0.014848804	-1.1730595	443.93677	1300.9155	0.3513694	0.00183918	-1.0459172	298.01248	828.4182	LOC_Os01g58270.1         	cucumisin precursor, putative	other
gb_UNIGENE13848	0.35462195	0.02295262	-1.036703	215.30356	568.625	0.44614664	0.0034641097	-0.8071076	167.15884	362.20837	LOC_Os01g58270.1         	cucumisin precursor, putative	other
gb_UNIGENE8307	3.0217216	0.00017849528	1.1058267	769.2008	249.2213	2.1875741	0.067560494	0.7827933	444.35223	202.96878	LOC_Os02g17090.1         	cucumisin precursor	other
gb_UNIGENE15533	4.0900164	0.0016758216	1.408549	44.351227	10.764824	2.586858	0.05709377	0.950444	105.27049	51.141026	LOC_Os02g17090.1         	cucumisin precursor	other
gb_UNIGENE13114	4.547274	0.0003826608	1.5145279	34084.223	7148.0205	6.7419834	0.000085222535	1.9083542	49120.977	7253.425	LOC_Os10g33270.1         	cyanate hydratase	other
gb_UNIGENE13530	0.40608603	0.0051115095	-0.9011903	1128.4972	2640.2144	0.54947996	0.0050982945	-0.598783	1513.7301	2691.3042	LOC_Os01g17390.1         	cyclin-like F-box	other
gb_UNIGENE16761	2.7669775	0.0048436546	1.0177556	1015.2175	364.69168	3.5631387	0.001263887	1.2706418	675.4225	183.12576	LOC_Os01g11340.1         	CYP710A1	other
gb_UNIGENE2988	0.24382222	0.019222481	-1.4113159	8876.825	28589.516	0.23807774	0.010246734	-1.435158	4607.7803	15753.873	LOC_Os04g01710.1         	cysteine proteinase At4g11310 precursor	other
gb_UNIGENE1302	0.47768247	0.0009832286	-0.73880905	1034.5715	2132.8518	0.57588553	0.0018169088	-0.5518464	2503.8228	4300.946	LOC_Os12g42980.2         	cysteine synthase	other
gb_UNIGENE4271	2.8745117	0.0031069883	1.0558828	7556.3325	2580.7139	4.340998	0.00034227193	1.4681044	1692.4724	393.36237	LOC_Os11g28060.1         	cytochrome P450 71C4, putative	other
gb_UNIGENE18990	11.920349	0.00010489687	2.478247	678.85095	57.116978	11.176764	0.0001522717	2.413837	117.11425	10.399439	LOC_Os02g09200.1         	cytochrome P450 71D10	other
gb_UNIGENE22570	0.48477924	0.014113811	-0.72406167	256.962	501.9437	0.57698435	0.0024333182	-0.5499401	286.89902	497.84695	LOC_Os03g37080.1         	cytochrome P450 71E1, putative	other
gb_UNIGENE17284	3.873024	0.0012838795	1.3540356	8283.436	2130.196	4.2898846	0.00080343697	1.4562598	7278.0273	1666.5503	LOC_Os01g43851.1         	cytochrome P450 72A1	other
gb_UNIGENE20974	8.78662	0.005034513	2.1732302	494.55423	50.000877	5.0524487	0.0038235064	1.619873	252.91049	53.30591	LOC_Os01g43750.1         	cytochrome P450 72A1	other
gb_UNIGENE3096	0.38389415	0.020033173	-0.95738846	6219.185	16519.344	0.5788624	0.23633727	-0.5466904	2001.1124	4008.3418	LOC_Os03g12500.1         	cytochrome P450 74A2	other
gb_UNIGENE20828	2.1716435	0.0030523187	0.7754842	299.87024	139.51935	2.2712097	0.02757203	0.82031256	242.45775	108.96549	LOC_Os10g08540.1         	cytochrome P450 76B1	other
gb_UNIGENE1522	12.175537	0.0015894786	2.4994287	18414.25	1592.9451	14.813572	0.00070935395	2.6955438	11916.333	802.1098	LOC_Os06g19070.1         	cytochrome P450 76C2	other
gb_UNIGENE15786	0.4931012	0.0061503695	-0.70704085	1482.935	2982.6575	0.48754188	0.05987218	-0.7183791	1583.6819	3169.3125	LOC_Os09g35940.1         	cytochrome P450 78A3	other
gb_UNIGENE11969	4.224143	0.0063077365	1.4408164	257.42474	61.706142	5.649258	0.014989327	1.7315242	162.79825	32.456757	LOC_Os10g36848.1         	cytochrome P450 84A1	other
gb_UNIGENE12508	0.4372175	0.01938106	-0.8273245	816.8247	1767.1809	0.5316798	0.010252536	-0.63171387	1270.5123	2308.031	LOC_Os03g12260.1         	cytochrome P450 86A2	other
gb_UNIGENE13825	0.4873963	0.046940394	-0.7186777	299.774	576.5496	0.4244962	0.0060932543	-0.85685223	85.2472	200.75552	LOC_Os06g02019.1         	cytochrome P450 88A1	other
gb_UNIGENE10985	10.9067135	0.008034578	2.3893785	2924.8325	305.88672	13.52452	0.00092890154	2.6045043	2693.9724	199.11063	LOC_Os02g01890.1         	cytochrome P450 89A2	other
gb_UNIGENE14534	0.52135634	0.027651824	-0.65132153	1946.3545	3493.7993	0.27035654	0.012575686	-1.3080137	95.69385	298.972	LOC_Os08g05620.1         	cytochrome P450 89A2	other
gb_UNIGENE23669	0.4742136	0.14299054	-0.74609745	986.43274	1583.9264	0.32028693	0.015065953	-1.138538	370.67776	1071.4487	LOC_Os04g01140.2         	cytochrome P450 93A2	other
gb_UNIGENE25605	8.9926195	0.000047126992	2.1964042	278.84653	30.821966	6.8197265	0.000612744	1.9198194	236.81026	35.007454	LOC_Os04g09920.1         	cytochrome P450 CYP99A1	other
gb_UNIGENE25596	0.49704087	0.00213618	-0.69908303	1289.435	2584.876	0.5273612	0.0048716757	-0.6398696	950.9355	1766.9832	LOC_Os04g09920.1         	cytochrome P450 CYP99A1	other
gb_UNIGENE25363	0.15077342	0.018951058	-1.8919771	597.5303	3475.8057	0.30497038	0.009875064	-1.1875407	1222.9725	3397.0247	LOC_Os05g31040.1         	cytokinin dehydrogenase 1 precursor	other
gb_UNIGENE24041	6.5076814	0.00084033265	1.8729832	1362.8575	214.15857	5.133085	0.0003335579	1.6357068	617.98425	118.74989	LOC_Os01g59100.1         	cytokinin-N-glucosyltransferase 1	other
gb_UNIGENE14077	3.336053	0.032614928	1.2047883	1714.9725	473.97305	4.7900624	0.0041072723	1.5665435	2556.23	494.3975	LOC_Os01g45110.1         	cytokinin-O-glucosyltransferase 1	other
gb_UNIGENE14742	7.5763535	0.00092561863	2.025032	7070.1025	969.0868	5.082559	0.0005158506	1.6258149	7246.24	1425.85	LOC_Os10g30560.1         	cytokinin-O-glucosyltransferase 2	other
gb_UNIGENE11709	12.766675	0.001349305	2.5468383	7050.9346	594.6958	13.240595	0.00026413516	2.5832875	1550.6326	119.679886	LOC_Os02g36830.1         	cytokinin-O-glucosyltransferase 2, putative	other
gb_UNIGENE7629	0.34266937	0.013559584	-1.0709893	5201.17	14256.795	0.3200895	0.004447258	-1.1391547	3831.3926	11246.747	LOC_Os01g53240.1         	dehydration-induced protein RD22-like protein 2	other
gb_UNIGENE13523	2.0486712	0.035837144	0.7171914	71.413734	37.18817	1.1372889	0.55011207	0.12864734	200.2696	218.67993	LOC_Os02g45450.1         	dehydration-responsive element-binding protein 1A	other
gb_UNIGENE2856	0.48833498	0.0053631533	-0.71675366	12875.8	26256.791	0.704739	0.0031766505	-0.34992778	10945.697	15677.3	LOC_Os02g44870.1         	dehydrin COR410	other
gb_UNIGENE22417	3.0153823	0.027364435	1.1037266	121.9635	41.67991	2.153208	0.025544526	0.76695883	57.026924	29.445396	LOC_Os01g55974.2         	deoxycytidylate deaminase	other
gb_UNIGENE5427	2.3516521	0.00016250047	0.85511816	6793.305	2889.4917	2.0116634	0.0014170094	0.6989619	5526.867	2746.941	LOC_Os04g54200.1         	diacylglycerol kinase	other
gb_UNIGENE5428	2.4724345	0.0007413314	0.90520334	1065.775	432.26123	2.220772	0.002484247	0.7978549	1811.6226	813.2709	LOC_Os04g54200.1         	diacylglycerol kinase	other
gb_UNIGENE17287	0.49032947	0.003397718	-0.7126777	1707.6375	3396.1453	0.6035992	0.0063516377	-0.50484484	1621.1875	2656.5945	LOC_Os03g16140.3         	digalactosyldiacylglycerol synthase 2	other
gb_UNIGENE11163	0.4316131	0.004379226	-0.84022576	1965.25	4551.119	0.69738376	0.087586544	-0.36041942	1734.015	2604.8	LOC_Os10g42620.1         	dihydroflavonol-4-reductase	other
gb_UNIGENE14426	1.4304312	0.04883583	0.35797596	2854.7424	2013.5698	7.427979	0.0033840728	2.0052538	517.4515	74.50332	LOC_Os09g25150.3         	dihydroflavonol-4-reductase	other
gb_UNIGENE6799	0.53287435	0.03285277	-0.62946963	4728.3325	8264.776	0.38273445	0.0054901326	-0.9604139	1686.9115	4186.727	LOC_Os09g25150.1         	dihydroflavonol-4-reductase	other
gb_UNIGENE18793	0.46348888	0.037404113	-0.7689729	519.04095	1096.3888	0.6071767	0.002843385	-0.49893543	153.783	250.80194	LOC_Os11g10800.1         	dirigent-like protein pDIR14	other
gb_UNIGENE15515	4.820116	0.0006195438	1.572798	2959.3228	604.04614	3.8688507	0.0016986242	1.3529575	1682.9633	418.45026	LOC_Os07g42570.1         	dirigent-like protein pDIR17, putative	other
gb_UNIGENE11792	3.0953054	0.003583092	1.1298866	7546.6577	2454.12	4.1529155	0.0054050423	1.4238106	3744.4724	890.35394	LOC_Os01g16750.1         	disulfide oxidoreductase/ monooxygenase/ oxidoreductase, putative	other
gb_UNIGENE8578	0.34781882	0.013249035	-1.0560735	2062.6077	5466.258	0.36801323	0.0062215007	-0.99963635	1316.77	3658.2705	LOC_Os04g23550.1         	DNA binding like	other
gb_UNIGENE15608	2.178023	0.0057161837	0.7784176	1668.6799	786.60864	1.6814829	0.0073827286	0.5196761	1308.2781	787.69617	LOC_Os02g03270.1         	DNA binding protein	other
gb_UNIGENE23443	0.32192597	0.068414405	-1.1334337	59.246025	131.88158	0.21573903	0.0040513	-1.5336858	17.262014	73.89965	LOC_Os03g21970.1         	DNA binding protein	other
gb_UNIGENE14535	0.08435589	0.06633153	-2.4727106	84.23778	296.1376	0.05988248	0.01926506	-2.8153713	15.351675	166.25224	LOC_Os02g51320.1         	DNA binding protein	other
gb_UNIGENE18020	1.9886556	0.0089454325	0.6874588	1012.4055	490.7798	2.1635618	0.00564909	0.7717559	1310.4666	595.0392	LOC_Os11g45740.1         	DNA binding protein	other
gb_UNIGENE18051	0.7674701	0.28606033	-0.2646557	222.28	277.15485	0.44726136	0.023436097	-0.80461216	654.1317	1632.4326	LOC_Os12g33950.1         	DNA binding protein, putative	other
gb_UNIGENE8287	4.0879536	0.0024049352	1.4080445	230.78275	55.39916	1.6467307	0.0064085023	0.49879193	150.73274	92.243965	LOC_Os01g36390.1         	DNA replication licensing factor mcm4	other
gb_UNIGENE19699	2.1891637	0.03802726	0.7835196	738.4485	351.53317	1.8726101	0.0010153587	0.6273332	992.697	532.2794	LOC_Os05g47650.1         	DNA-binding protein RAV1	other
gb_UNIGENE3533	0.5544598	0.041450404	-0.58976096	38497.05	68170.06	0.45552516	0.000053759028	-0.78630435	21603.65	47756.625	LOC_Os01g52690.1         	DNA-directed RNA polymerase II largest subunit	other
gb_UNIGENE13151	2.418617	0.0029960407	0.8831959	3382.71	1404.9607	1.9303384	0.0048752488	0.6576953	2238.0574	1156.3085	LOC_Os01g74580.2         	dnaJ homolog subfamily C member 7	other
gb_UNIGENE10137	2.9556258	0.00038588897	1.0837104	9131.595	3045.1711	2.8738508	0.0023675722	1.0556529	7715.0054	2686.2676	LOC_Os06g44160.1         	dnaJ protein	other
gb_UNIGENE24922	2.2954397	0.011320243	0.83092445	1562.7476	699.0464	1.4518589	0.07698008	0.3728447	2920.0698	2102.3828	LOC_Os10g34170.1         	domain found in Dishevelled, Egl-10, and Pleckstrin family protein	other
gb_UNIGENE10480	2.94597	0.00070499745	1.0804381	4041.7722	1355.6569	2.3040962	0.0002518606	0.8346885	2139.3674	937.0581	LOC_Os04g15690.1         	DSBA-like thioredoxin domain containing protein	other
gb_UNIGENE10898	2.498112	0.0032568162	0.9155352	2393.875	879.04846	2.3957767	0.007655045	0.8737075	890.53296	347.97287	LOC_Os02g13380.1         	early nodulin 93	other
gb_UNIGENE2057	0.46851245	0.039823223	-0.7581926	518.7645	928.82764						LOC_Os06g05000.1         	early nodulin 93	other
gb_UNIGENE11653	8.712171	0.0006894306	2.164721	20108.375	2415.5283	11.754373	0.00029559288	2.4642253	16249.95	1404.7421	LOC_Os07g43800.1         	EF hand family protein	other
gb_UNIGENE4899	8.602056	0.0015392344	2.1520011	4249.145	492.90002	12.630363	0.000089320296	2.5361037	5165.6523	409.2142	LOC_Os06g46950.1         	EF-hand Ca2+-binding protein CCD1	other
gb_UNIGENE15981	3.6861725	0.004202434	1.3045887	5181.157	1426.0131	2.9894485	0.0033864456	1.095089	13840.225	4665.85	LOC_Os02g03020.1         	EF-hand Ca2+-binding protein CCD1	other
gb_UNIGENE12124	2.0630188	0.010349659	0.7241703	2515.2202	1220.9182	2.0377073	0.0023816873	0.7118253	3766.5176	1851.0015	LOC_Os07g41410.1         	EGG APPARATUS-1 protein	other
gb_UNIGENE9479	0.44303638	0.030304354	-0.81410336	5611.9424	11385.459	0.6002329	0.005340748	-0.5104375	10715.758	17663.418	LOC_Os07g30670.1         	electron carrier/ electron transporter/ iron ion binding protein	other
gb_UNIGENE21479	0.45269465	0.039249394	-0.79253745	4271.3022	8357.581	0.58648694	0.0050852587	-0.5336049	7528.3003	12697.333	LOC_Os07g30670.1         	electron carrier/ electron transporter/ iron ion binding protein	other
gb_UNIGENE18901	0.4673626	0.0053553125	-0.76064986	373.18848	789.438	0.4878316	0.006380619	-0.717785	342.7495	705.8154	LOC_Os05g11990.1         	electron transporter	other
gb_UNIGENE11789	4.9788384	0.00056999695	1.6051966	342.532	68.194534	3.223292	0.0010167492	1.1704032	566.9595	173.67871	LOC_Os01g62430.3         	elicitor-responsive protein 1	other
gb_UNIGENE10193	3.989076	0.001997117	1.3835596	2421.5974	613.1228	2.9583137	0.0037674163	1.0846194	1632.91	541.7501	LOC_Os01g62430.3         	elicitor-responsive protein 1	other
gb_UNIGENE5942	2.441636	0.0010901899	0.89266837	2039.7576	820.9508	2.0620317	0.0010439964	0.7236918	2027.15	977.51	LOC_Os04g44870.1         	elicitor-responsive protein 3	other
gb_UNIGENE5943	2.0694063	0.0010322812	0.7272618	5020.8022	2421.3013	1.77421	0.000115917035	0.57335526	4809.46	2704.7664	LOC_Os04g44870.1         	elicitor-responsive protein 3	other
gb_UNIGENE24322	6.7225666	0.00046694034	1.90547	794.9505	117.92455	3.2063012	0.0060097203	1.165118	1191.5068	378.80838	LOC_Os06g19370.2         	embryogenesis transmembrane protein	other
gb_UNIGENE7879	2.7184384	0.00008336417	1.0000576	1797.37	661.2344	3.2469962	0.00032476583	1.1777303	1982.96	612.0232	LOC_Os01g65070.2         	embryogenesis-associated protein EMB8	other
gb_UNIGENE22839	6.4500256	0.013312837	1.8640841	862.44476	197.99872	6.5833817	0.008012744	1.8845485	762.98224	128.03696	LOC_Os01g40660.1         	embryonic abundant protein-like	other
gb_UNIGENE4147	0.4180623	0.053472288	-0.8721248	438.25354	858.7773	0.22695197	0.024186695	-1.4830168	120.21756	425.31833	LOC_Os03g52630.1         	endo-1,4-beta-glucanase Cel1	other
gb_UNIGENE416	3.583273	0.00075366907	1.2762766	20334.125	5706.633	2.455506	0.0021294192	0.8983329	15224.65	6222.6123	LOC_Os06g50300.2         	endoplasmin homolog precursor	other
gb_UNIGENE71	3.375029	0.0017650902	1.216404	22493.275	6838.907	2.3840287	0.0037076608	0.8687918	17426.1	7288.9224	LOC_Os06g50300.2         	endoplasmin homolog precursor	other
gb_UNIGENE91	3.2057955	0.0027225136	1.1649603	4440.9	1387.0856	2.4457912	0.0044066017	0.89436865	3241.8525	1329.5538	LOC_Os06g50300.1         	endoplasmin homolog precursor	other
gb_UNIGENE17025	2.520829	0.0019171631	0.92458785	576.7995	229.69302	2.0759108	0.004484781	0.73039997	551.56976	266.087	LOC_Os09g20820.1         	enolase	other
gb_UNIGENE26204	0.5515936	0.0042610066	-0.5949437	106.62847	199.58093	0.36977005	0.0031993883	-0.99487394	167.9615	471.34943	LOC_Os04g52210.1         	ent-kaurene synthase B, chloroplast precursor	other
gb_UNIGENE4942	2.6270392	0.01635427	0.9658574	1316.5275	530.82837	2.9379592	0.0077300463	1.0777152	1231.3824	415.66492	LOC_Os02g32520.2         	ERD1 protein, chloroplast precursor	other
gb_UNIGENE10057	4.6695237	0.066368856	1.5410571	202.81424	48.02993	4.1062007	0.022187779	1.4124982	51.769753	13.294924	LOC_Os01g70850.1         	esterase PIR7B	other
gb_UNIGENE1402	4.811083	0.0034167764	1.5709221	801.06647	178.07298	3.606928	0.0062681255	1.2828565	754.536	218.59761	LOC_Os07g39740.1         	esterase precursor	other
gb_UNIGENE7354	4.538746	0.00039364482	1.5126507	1167.5	260.8759	3.2609694	0.00081252516	1.1820245	487.68048	149.73093	LOC_Os05g26660.1         	expressed protein	other
gb_UNIGENE7789	0.41399157	0.074604824	-0.88190967	187.32838	402.25452	0.3741069	0.004297988	-0.98321366	52.791	133.10397	LOC_Os05g48890.1         	fasciclin-like arabinogalactan protein 7 precursor	other
gb_UNIGENE8273	0.4825146	0.06615888	-0.72874415	236.64438	431.17517	0.22406147	0.010997297	-1.4958348	82.94205	335.94333	LOC_Os01g47780.1         	fasciclin-like arabinogalactan protein 7 precursor	other
gb_UNIGENE6782	0.49834663	0.13161401	-0.6964594	1817.0765	3317.4858	0.27637148	0.025099546	-1.2860094	782.07104	2394.7798	LOC_Os05g48900.1         	fasciclin-like arabinogalactan protein 7 precursor	other
gb_UNIGENE18128	0.41556293	0.064037606	-0.87812126	20.233675	40.093952	0.42843133	0.0038483697	-0.84762484	10.51495	24.941133	LOC_Os09g30010.1         	fasciclin-like arabinogalactan protein 8 precursor	other
gb_UNIGENE6939	0.6382305	0.049246415	-0.44905576	5126.12	7632.365	0.46938598	0.014702873	-0.75632983	3664.9546	7286.675	LOC_Os08g23180.1         	fasciclin-like arabinogalactan protein 8 precursor	other
gb_UNIGENE22130	10.82338	0.0007366401	2.3817086	129.52292	11.920082	11.312308	0.0028069774	2.4258914	127.47668	10	LOC_Os05g49900.1         	fatty acid elongase	other
gb_UNIGENE14595	0.38764656	0.035319112	-0.9476613	1593.781	4583.5605	0.6831103	0.12033001	-0.381099	3674.4302	5559.082	LOC_Os09g30170.2         	F-box protein interaction domain containing protein	other
gb_UNIGENE3886	2.9288588	0.0034175008	1.0746129	22072.2	7604.7764	2.8157992	0.0005555014	1.0352461	16703.875	5922.5957	LOC_Os03g61960.1         	ferredoxin-3, chloroplast precursor	other
gb_UNIGENE17766	0.39525434	0.08243352	-0.9282258	1179.5901	2618.2769	0.30665037	0.030240988	-1.182047	554.682	1625.8882	LOC_Os04g33630.1         	ferredoxin-3, chloroplast precursor	other
gb_UNIGENE8863	2.0099056	0.00033035636	0.6980877	633.8428	319.02838	2.0388024	0.011646292	0.7123625	40.978424	21.778978	LOC_Os09g10910.3         	fiber protein Fb34	other
gb_UNIGENE12081	2.680223	0.013521425	0.9859	93.671974	35.944656	1.9698687	0.08999221	0.6779669	73.85385	41.922916	LOC_Os10g35294.1         	fiber protein Fb34	other
gb_UNIGENE26313	2.7807627	0.01226041	1.0227252	936.53925	300.40204	2.2103338	0.01930229	0.7931435	475.89526	214.43544	LOC_Os09g26960.1         	flavonoid 3-monooxygenase	other
gb_UNIGENE12075	2.409607	0.03678101	0.8794637	3110.7625	1389.8544	2.443421	0.029209528	0.89339906	2166.1	983.9899	LOC_Os07g41460.1         	flavonol 4-sulfotransferase	other
gb_UNIGENE12910	0.30406886	0.031482898	-1.1905011	9.255423	25.390862	0.8497695	0.12819266	-0.16279018	768.4875	894.8779	LOC_Os09g08190.1         	flavonol 4-sulfotransferase	other
gb_UNIGENE8962	0.12934533	0.025342304	-2.0452695	112.2169	586.99524	0.13199632	0.01310351	-2.0249813	77.18335	446.91495	LOC_Os04g44460.1         	flavonol sulfotransferase-like	other
gb_UNIGENE12724	4.0392413	0.0029242886	1.3960569	7979.3604	1925.6243	5.0100584	0.0060157604	1.6114476	4431.9297	888.3235	LOC_Os03g03034.2         	flavonol synthase/flavanone 3-hydroxylase	other
gb_UNIGENE4273	12.284844	0.0004531814	2.5083663	2103.92	176.24399	9.978414	0.00018407499	2.300424	1973.525	193.18799	LOC_Os06g08032.1         	flavonol synthase/flavanone 3-hydroxylase	other
gb_UNIGENE3891	0.4847237	0.015288959	-0.7241763	2328.1125	4863.1587	0.7860206	0.041960813	-0.24077228	3024.22	3833.9438	LOC_Os09g07020.1         	flavonol synthase-like protein	other
gb_UNIGENE5876	8.983776	0.005533683	2.1954203	164.5335	21.018147	10.0194645	0.0017988763	2.3045297	115.727	10.831392	LOC_Os01g08110.1         	flavonol-3-O-glycoside-7-O-glucosyltransferase 1	other
gb_UNIGENE5217	2.4752893	0.008359029	0.9063573	1537.7958	583.79205	1.8283117	0.028167656	0.60339296	393.86475	222.05646	LOC_Os05g42190.1         	flavoprotein wrbA	other
gb_UNIGENE9844	0.5381189	0.116949245	-0.6196757	2276.5676	4372.8086	0.3095775	0.015816042	-1.1725469	525.7132	1674.7104	LOC_Os07g47450.1         	flowering promoting factor-like 1	other
gb_UNIGENE7279	0.37672144	0.03524753	-0.9762493	898.769	2139.3096	0.45890552	0.019904586	-0.77891093	1000.8858	2058.3174	LOC_Os01g32730.1         	FLU	other
gb_UNIGENE101	2.6162772	0.0004958477	0.9617524	3345.1726	1271.9591	1.7405746	0.005631887	0.5542153	1736.63	986.9779	LOC_Os01g66940.1         	fructokinase-1	other
gb_UNIGENE3466	0.44719905	0.01899728	-0.80475146	204.46303	428.99603	0.52650845	0.012146252	-0.6414879	199.15025	380.86963	LOC_Os05g07130.2         	fructose-6-phosphate-2-kinase/fructose-2, 6-bisphosphatase	other
gb_UNIGENE3812	0.27453986	0.018408718	-1.2926588	423.90723	1643.5073	0.7951877	0.29583004	-0.2291771	576.9817	786.0369	LOC_Os07g48830.1         	galactinol synthase 3	other
gb_UNIGENE16931	3.1438427	0.007512718	1.1454458	1092.1958	384.1963	3.1608102	0.0060570813	1.1508284	1099.2268	350.406	LOC_Os02g52560.1         	galactoside 2-alpha-L-fucosyltransferase	other
gb_UNIGENE25864	2.048756	0.004016933	0.7172327	1113.8362	536.7948	1.6495903	0.01401933	0.5005269	926.58203	563.30896	LOC_Os06g12390.1         	Galactoside-binding lectin family protein	other
gb_UNIGENE18811	2.7098596	0.0020269845	0.9968968	290.66974	109.241585	2.3927004	0.0065590064	0.8724226	191.69775	81.719795	LOC_Os04g01280.1         	galactosylgalactosylxylosylprotein 3-beta-glucuronosyltransferase 1	other
gb_UNIGENE18214	5.6999125	0.0019225687	1.7404509	73.566055	13.271466	4.073945	0.0069448263	1.4046118	54.59022	13.92264	LOC_Os07g09670.2         	galactosyltransferase/ transferase, transferring hexosyl groups	other
gb_UNIGENE8778	0.32184687	0.03101912	-1.1336794	202.699	512.9688	0.23845576	0.007198431	-1.4335715	100.26098	375.79742	LOC_Os06g15620.1         	GAST1 protein precursor	other
gb_UNIGENE21295	0.3786083	0.0083619375	-0.97125316	787.67346	1956.5371	0.59027904	0.0005211206	-0.52715987	650.4178	1098.3379	LOC_Os06g49740.1         	GCIP-interacting protein	other
gb_UNIGENE631	3.1792688	0.0004115494	1.1566513	6459.04	2084.6506	1.8042692	0.0007387781	0.5901556	3187.1948	1762.3833	LOC_Os06g04620.1         	GDP-mannose 4,6 dehydratase 2	other
gb_UNIGENE9950	0.32026014	0.039304446	-1.1386217	20.086374	65.56197	0.6765859	0.24592926	-0.39069584	113.92445	160.37756	LOC_Os01g11760.1         	GDSL-like Lipase/Acylhydrolase family protein	other
gb_UNIGENE3155	2.1766398	0.0027417634	0.7777823	1402.509	615.30164	2.4887593	0.014390683	0.9117843	6468.6875	2367.7124	LOC_Os08g08990.1         	germin-like protein subfamily 1 member 11 precursor	other
gb_UNIGENE3151	3.133419	0.0056910873	1.1421248	808.19226	247.40732	4.662544	0.0008007095	1.5395612	16258.475	3393.954	LOC_Os08g08990.1         	germin-like protein subfamily 1 member 11 precursor	other
gb_UNIGENE3157	1.5792944	0.0073567424	0.4569782	1134.1925	725.4684	2.8126268	0.00072226056	1.0341189	2665.4775	941.58966	LOC_Os08g09080.2         	germin-like protein subfamily 1 member 11 precursor	other
gb_UNIGENE3152	2.0714445	0.045198392	0.72824615	5547.8877	2657.083	2.5645893	0.008351332	0.9417983	21074	8387.95	LOC_Os08g08990.1         	germin-like protein subfamily 1 member 11 precursor	other
gb_UNIGENE3156	1.8891934	0.044796553	0.63614994	8376.138	4391.4824	2.2333887	0.017598793	0.80351996	29997.4	13595.809	LOC_Os08g09010.1         	germin-like protein subfamily 1 member 11 precursor	other
gb_UNIGENE17901	1.960198	0.04344528	0.6730455	30.175426	14.306875	2.0409348	0.0023467713	0.71340793	21.0032	10.193474	LOC_Os05g10830.1         	germin-like protein subfamily 1 member 11 precursor, putative	other
gb_UNIGENE1517	0.25104317	0.033901382	-1.3821304	734.96375	3904.2734	1.6333015	0.104172155	0.49060345	818.3783	505.81927	LOC_Os08g08980.1         	germin-like protein subfamily 1 member 7 precursor	other
gb_UNIGENE10860	8.952738	0.00008961074	2.1919594	6913.173	790.31604	5.731909	0.00082453113	1.7460486	5427.2227	960.94775	LOC_Os03g58980.1         	germin-like protein subfamily T member 2 precursor	other
gb_UNIGENE18334	2.8738887	0.0005463731	1.0556661	2035.8401	709.65186	1.6716162	0.0034348576	0.51379097	1424.0925	851.478	LOC_Os12g03720.2         	glucosamine--fructose-6-phosphate aminotransferase	other
gb_UNIGENE3308	2.0102413	0.0024082705	0.6982547	8642.395	4314.4053	2.174222	0.005165331	0.77667093	7810.01	3561.588	LOC_Os02g38840.2         	glucose-6-phosphate 1-dehydrogenase, cytoplasmic isoform	other
gb_UNIGENE1385	4.383049	0.00035084324	1.4777446	15799.05	3653.7415	2.3970342	0.00044356778	0.8742322	19324.926	8144.9175	LOC_Os03g51080.1         	glutamate decarboxylase	other
gb_UNIGENE23499	2.5676506	0.005305807	0.9429913	258.51474	102.603966	1.6698686	0.0041778386	0.51274496	266.116	159.50592	LOC_Os09g26160.1         	glutamate receptor 2.9 precursor	other
gb_UNIGENE9764	3.1272123	0.0057915705	1.140142	327.59027	103.36723	1.6086166	0.043903925	0.47537458	195.6525	128.0804	LOC_Os10g38710.1         	glutathione S-transferase GSTU6	other
gb_UNIGENE6008	3.6922193	0.021984428	1.3062277	509.43552	123.45079	3.653052	0.040465537	1.295563	313.536	81.53061	LOC_Os10g38140.1         	glutathione S-transferase GSTU6	other
gb_UNIGENE21604	0.4813221	0.01719493	-0.7312186	181.53825	386.2677	0.7690591	0.16031915	-0.2625874	179.85425	234.24489	LOC_Os12g16200.1         	glutathione synthetase, chloroplast precursor	other
gb_UNIGENE865	2.0260017	0.007964581	0.7060642	41274.824	21059.463	1.5176511	0.00858514	0.4171638	27835.725	18256.84	LOC_Os04g40950.1         	glyceraldehyde-3-phosphate dehydrogenase, cytosolic 3	other
gb_UNIGENE8749	3.0699303	0.0017486954	1.1216549	563.89124	179.23187	2.2489176	0.007850616	0.810449	530.751	239.80885	LOC_Os08g39350.1         	glycerophosphoryl diester phosphodiesterase family protein	other
gb_UNIGENE9141	3.7397776	0.0002553034	1.3190261	5898.492	1561.0275	2.4472148	0.0009656961	0.89495057	5002.2397	2031.6545	LOC_Os03g40670.1         	glycerophosphoryl diester phosphodiesterase precursor	other
gb_UNIGENE2994	0.51063585	0.10805339	-0.6720985	15373.274	24943.258	0.4472748	0.028861271	-0.8045821	12267.676	24980.312	LOC_Os10g37180.1         	glycine cleavage system H protein, mitochondrial precursor	other
gb_UNIGENE8722	6.8709564	0.00032541546	1.9273033	972.35425	145.66728	6.0034814	0.009298789	1.7923396	865.645	177.92009	LOC_Os03g03350.1         	glycoside hydrolase, family 28	other
gb_UNIGENE25252	2.4128962	0.00027815695	0.8808277	1131.9929	468.1059	1.9231339	0.0053853067	0.65395606	620.09045	325.6313	LOC_Os02g41520.1         	glycosyl transferase family 8 protein	other
gb_UNIGENE16651	0.48840755	0.0055570873	-0.71660507	257.22824	510.34027	0.4997453	0.0015592696	-0.6936567	160.40076	323.19363	LOC_Os01g04920.1         	glycosyl transferase, group 1 family protein	other
gb_UNIGENE13143	4.130644	0.0005401179	1.4184333	678.90826	164.70262	3.4001045	0.0049279397	1.2238061	136.84024	41.514954	LOC_Os02g04250.3         	glycosyltransferase	other
gb_UNIGENE14311	4.77715	0.0015393592	1.5638442	905.6555	194.45924	2.6522288	0.012803923	0.9754003	796.5643	321.91333	LOC_Os06g28124.1         	glycosyltransferase	other
gb_UNIGENE9352	2.2651043	0.0008987253	0.8176208	5182.165	2292.9548	1.7723632	0.0059738737	0.5723138	7174.22	4061.1458	LOC_Os01g02920.1         	glycosyltransferase	other
gb_UNIGENE9351	2.255647	0.0035079294	0.81343687	2991.2773	1335.9867	1.793769	0.00026257374	0.584319	1614.3074	898.21204	LOC_Os01g02920.1         	glycosyltransferase	other
gb_UNIGENE13583	2.0140238	0.004380359	0.7001346	155.17325	78.3281	1.8673228	0.029826049	0.62450576	171.09175	97.03554	LOC_Os01g72610.1         	glycosyltransferase	other
gb_UNIGENE11664	3.7067006	0.018930707	1.3101422	127.543625	38.849525	2.6252248	0.04037815	0.9651665	131.0005	54.9486	LOC_Os06g28124.1         	glycosyltransferase	other
gb_UNIGENE2387	0.49075007	0.012341632	-0.7118203	352.57425	697.4515	0.6073691	0.042937107	-0.4986185	483.151	800.34766	LOC_Os06g01400.1         	glycyl-tRNA synthetase 2, chloroplast/mitochondrial precursor	other
gb_UNIGENE16335	0.39497846	0.0708784	-0.928924	529.966	1168.0918	0.4118292	0.0066884756	-0.8871466	109.30733	256.8329	LOC_Os08g38170.1         	GMP synthase	other
gb_UNIGENE6874	0.9186766	0.7493996	-0.084821135	94.17515	102.346664	0.29778087	0.0015354481	-1.2113974	9.48407	30.370312	LOC_Os07g45060.1         	GPI-anchored protein At5g19240 precursor	other
gb_UNIGENE20058	3.148693	0.0015689646	1.1469874	2804.5876	915.5359	3.0289562	0.0010385463	1.1082181	900.8875	297.31467	LOC_Os02g48980.1         	GPI-anchored protein	other
gb_UNIGENE7044	2.5524666	0.000698874	0.93706024	1350.8275	531.2307	1.860362	0.00081192655	0.6207711	1065.9199	574.02527	LOC_Os01g61970.1         	protein GPR108 precursor	other
gb_UNIGENE12208	0.41928935	0.018139383	-0.86919403	224.56775	519.6538	0.38437495	0.0049230796	-0.95613676	125.68225	310.1025	LOC_Os07g22930.4         	granule-bound starch synthase 1b, chloroplast precursor	other
gb_UNIGENE12060	0.58695126	0.0065222257	-0.53281355	168.54599	287.14212	0.48273554	0.005204606	-0.7282863	74.0733	151.04813	LOC_Os03g51970.1         	growth-regulating factor 1	other
gb_UNIGENE7701	2.0118477	0.009014368	0.6990535	132.50375	67.98271	1.2294457	0.006456508	0.20656346	245.91	199.3473	LOC_Os01g27730.1         	guanine nucleotide-binding protein-like 3	other
gb_UNIGENE12088	0.3891838	0.0045425296	-0.9437036	432.99152	1062.0267	0.4378796	0.0036785465	-0.8258113	897.54047	1976.864	LOC_Os06g43620.1         	haemolysin-III related family protein	other
gb_UNIGENE3744	2.4141948	0.0019750376	0.8813658	7868.832	3221.61	2.1517668	0.003330354	0.76628923	6853.015	3198.4414	LOC_Os04g58850.1         	harpin inducing protein	other
gb_UNIGENE20575	5.250028	0.0011136596	1.6582334	229.24625	43.76503	4.299687	0.008568021	1.4585422	55.519226	13.607959	LOC_Os05g45070.1         	harpin-induced protein	other
gb_UNIGENE10379	0.60130143	0.0788679	-0.5086589	241.47989	373.74054	0.42008734	0.020011986	-0.86729264	137.17014	305.61334	LOC_Os01g09880.1         	harpin-induced protein	other
gb_UNIGENE16386	2.2074957	0.016744835	0.79185873	2553.445	1128.3206	1.938835	0.010142501	0.66208726	2016.44	1046.8499	LOC_Os10g33140.1         	hcrVf2 protein	other
gb_UNIGENE21775	9.421475	0.000052631036	2.2429917	140.61389	15.428959						LOC_Os05g44340.1         	heat shock protein 101	other
gb_UNIGENE6555	3.354715	0.022447819	1.2103668	2078.66	612.5635	1.2378572	0.44337568	0.21338187	388.4125	328.72327	LOC_Os06g11610.1         	heat shock 22 kDa protein, mitochondrial precursor	other
gb_UNIGENE6557	2.3581371	0.022554344	0.85787195	3579.6875	1495.73	1.0677404	0.7551112	0.06554465	898.8477	834.1894	LOC_Os06g11610.1         	heat shock 22 kDa protein, mitochondrial precursor	other
gb_UNIGENE6559	2.492842	0.026479982	0.9134234	1305.082	510.32495	1.0465449	0.7915479	0.04549414	282.40976	269.00998	LOC_Os06g11610.1         	heat shock 22 kDa protein, mitochondrial precursor	other
gb_UNIGENE18961	8.2020645	0.0025653518	2.1043859	352.94525	45.504692	9.462667	0.0054696035	2.2473543	252.352	31.025696	LOC_Os05g45410.1         	heat shock factor	other
gb_UNIGENE10851	0.2992998	0.09072899	-1.2063096	163.10138	405.0383	0.22926567	0.024576955	-1.4728738	94.60892	336.69177	LOC_Os02g55870.1         	heparanase-like protein 3 precursor	other
gb_UNIGENE9060	2.4848752	0.0077907406	0.9102225	15163.75	6167.0156	2.0561357	0.014365685	0.72082835	13941.625	6786.0107	LOC_Os01g64110.1         	hevamine-A precursor	other
gb_UNIGENE17518	4.415147	0.00021916645	1.4850411	3415.9424	800.2994	4.2291784	0.000239964	1.4420078	3439.1226	812.20984	LOC_Os06g04900.1         	hexose carrier protein HEX6	other
gb_UNIGENE94	1.7436165	0.013887889	0.5559614	1206.9125	708.46796	2.1109877	0.016421882	0.7471559	1096.1183	517.63	LOC_Os11g05730.1         	histone H3	other
gb_UNIGENE179	0.44250613	0.066511355	-0.81530094	234.13008	489.28375	0.20852333	0.019407963	-1.5677043	74.87471	264.40234	LOC_Os11g05730.1         	histone H3	other
gb_UNIGENE25238	0.5492206	0.02790135	-0.5992551	74.79884	139.51266	0.41964597	0.012584181	-0.86834383	31.52067	60.607906	LOC_Os03g03164.2         	homeobox protein HD1	other
gb_UNIGENE18116	6.6372776	0.0008259831	1.8927019	3135.235	475.26453	9.774217	0.000970173	2.279748	4236.6997	397.94226	LOC_Os07g35560.3         	hydrolase, hydrolyzing O-glycosyl compounds	other
gb_UNIGENE8650	0.96480507	0.8670852	-0.035829186	72.762505	78.30925	3.3951688	0.029731555	1.2223535	472.50198	151.92459	LOC_Os06g03910.1         	hydrolase, NUDIX family protein	other
gb_UNIGENE3314	2.418883	0.002740922	0.8833059	45740.85	19129.201	2.0537755	0.0071237185	0.71967983	33862.58	16772.605	LOC_Os05g04700.1         	hydrophobic protein LTI6B	other
gb_UNIGENE3315	2.0137486	0.0026486712	0.69999796	5010.29	2515.983	1.6933745	0.0069712074	0.52672327	3654.815	2166.931	LOC_Os05g04700.1         	hydrophobic protein LTI6B	other
gb_UNIGENE3626	3.180006	0.003619389	1.1568831	12724.926	4087.394	3.040457	0.0027619002	1.1120079	17723.8	5875.747	LOC_Os05g51420.2         	hypersensitive-induced response protein	other
gb_UNIGENE8729	17.527933	0.0010609535	2.8637958	909.24	54.992256	6.9041963	0.012693585	1.9321294	623.296	107.2249	LOC_Os11g44300.1         	hypothetical protein	other
gb_UNIGENE17122	3.4841504	0.0022187058	1.2482243	5559.315	1597.1732	3.3615518	0.0012908347	1.2124027	4683.43	1382.0403	LOC_Os03g14110.1         	hypothetical protein	other
gb_UNIGENE12954	0.18817839	0.019069437	-1.6703649	98.28698	658.41675	0.46519223	0.028887277	-0.76530457	50.18965	114.59564	LOC_Os10g37870.1         	hypothetical protein	other
gb_UNIGENE21739	2.3708818	0.004677034	0.86326194	143.7955	58.53517	1.6623276	0.008720405	0.5082188	253.23825	152.13753	LOC_Os09g11780.1         	hypothetical protein	other
gb_UNIGENE14529	0.56274474	0.071195506	-0.5749292	849.3042	1521.8896	0.45577183	0.0060433415	-0.78576297	390.90698	841.99744	LOC_Os09g20870.1         	hypothetical protein	other
gb_UNIGENE22861	4.488045	0.002406407	1.5014173	3844.7576	851.31226	2.5712879	0.012530237	0.9444069	782.0847	298.2503	LOC_Os09g26310.1         	hypro1	other
gb_UNIGENE25420	7.6938534	0.00043324786	2.0404217	663.2147	89.79127	4.9739976	0.0034972185	1.6042238	563.08875	111.000435	LOC_Os02g50460.1         	immediate-early fungal elicitor protein CMPG1	other
gb_UNIGENE19921	4.85124	0.0013226098	1.5792344	743.4178	148.81866	3.5133855	0.0071912976	1.2565801	1162.7443	325.64642	LOC_Os06g13870.1         	immediate-early fungal elicitor protein CMPG1	other
gb_UNIGENE2895	0.5156699	0.0042713643	-0.6622884	2553.3125	4980.3335	0.48510185	0.015130955	-0.7233964	1919.2	3859.9636	LOC_Os07g08200.1         	inner envelope membrane protein, chloroplast precursor, putative	other
gb_UNIGENE6322	3.6501112	0.00031092096	1.2947576	6695.495	1860.4049	3.3906825	0.0023074008	1.2210312	5464.3677	1613.8052	LOC_Os07g39280.1         	integral membrane protein like	other
gb_UNIGENE17361	3.2776132	0.14806171	1.1871154	1016.9253	309.9829	8.939548	0.026569847	2.190485	784.89	114.31845	LOC_Os08g35310.1         	isoflavone-7-O-methytransferase 9, putative	other
gb_UNIGENE22343	2.000702	0.00869248	0.693498	1347.7151	667.9256	1.2489303	0.028321305	0.22228743	1112.962	892.7608	LOC_Os04g42310.1         	K+ channel tetramerisation domain containing protein	other
gb_UNIGENE13654	1.2228001	0.4379807	0.20114343	24.789225	21.531166	2.2510092	0.0038717848	0.81137866	1567.375	698.46246	LOC_Os12g31560.1         	protein kinase domain containing protein	other
gb_UNIGENE24882	2.0299015	0.010278332	0.7079873	9064.375	4538.532	1.9636765	0.0007116075	0.67481846	9489.763	4891.598	LOC_Os06g18000.1         	protein kinase domain containing protein	other
gb_UNIGENE17486	0.4246474	0.0065888423	-0.8564961	87.18267	192.04349	0.2937388	0.03643184	-1.2250643	55.39775	164.73318	LOC_Os04g45580.1         	kinesin heavy chain	other
gb_UNIGENE16311	2.5413513	0.00041750178	0.9326959	183.48349	72.819275	1.9006641	0.009617891	0.64220333	176.18901	93.723114	LOC_Os08g08230.1         	kinesin-like protein	other
gb_UNIGENE12316	4.746352	0.018216318	1.5573764	196.8812	43.620384	1.5431055	0.51388985	0.43379694	37.9803	28.03828	LOC_Os04g43650.2         	L-allo-threonine aldolase	other
gb_UNIGENE14651	8.104246	0.0010889055	2.0923882	84.42413	10	5.1845603	0.0014670497	1.6456851	53.58315	10	LOC_Os09g20090.1         	L-ascorbate oxidase precursor	other
gb_UNIGENE18447	0.3846631	0.010871749	-0.9553874	195.23492	475.57202	0.2256719	0.025534803	-1.4886731	98.81682	363.4139	LOC_Os01g62490.1         	L-ascorbate oxidase precursor	other
gb_UNIGENE10632	0.2841955	0.034164947	-1.2580929	681.8445	1915.8342	0.37628868	0.0010858766	-0.9773987	238.10175	633.0737	LOC_Os01g63190.2         	L-ascorbate oxidase precursor	other
gb_UNIGENE17768	3.0059092	0.002391112	1.1005801	3160.9526	1062.6875	2.485829	0.00084506924	0.91060627	3306.42	1318.4382	LOC_Os02g04170.3         	L-aspartate oxidase 1	other
gb_UNIGENE23149	2.9548085	0.0021275573	1.0834339	360.9568	120.09042	3.1266632	0.016468115	1.1399664	137.11513	39.702065	LOC_Os02g48210.1         	lectin-like protein kinase	other
gb_UNIGENE3564	8.615035	0.00042203712	2.153509	9574.68	1078.1089	7.779349	0.00041288137	2.0514727	3765.3848	477.97705	LOC_Os02g17940.6         	leucoanthocyanidin dioxygenase	other
gb_UNIGENE3565	9.18313	0.0009078679	2.2173681	11307.832	1180.5133	8.487191	0.0012051387	2.1385581	2415.4775	293.3328	LOC_Os02g17940.6         	leucoanthocyanidin dioxygenase	other
gb_UNIGENE3563	12.381934	0.0039672637	2.5162385	207.71825	19.00054	7.044736	0.0037643586	1.9522806	105.06832	16.205002	LOC_Os02g17940.6         	leucoanthocyanidin dioxygenase	other
gb_UNIGENE3568	7.737742	0.00068796374	2.04611	4447.7075	562.8504	0.37205675	0.47742406	-0.9887089	15.6229725	16.749313	LOC_Os02g17940.6         	leucoanthocyanidin dioxygenase	other
gb_UNIGENE11146	2.3761058	0.004035072	0.8654629	2048.7324	848.68427	2.190083	0.011432693	0.7839395	1499.7849	676.01086	LOC_Os06g44170.1         	leucoanthocyanidin reductase	other
gb_UNIGENE3699	4.707302	0.0021349792	1.549115	9756.968	2123.9119	3.2432945	0.0044783303	1.1765896	4754.3926	1484.3063	LOC_Os06g47200.1         	lipid binding protein	other
gb_UNIGENE12086	0.45798558	0.041574392	-0.7809176	100.670334	230.54803	0.56752723	0.025180286	-0.56646657	53.34645	97.06473	LOC_Os05g41030.1         	lipid binding protein	other
gb_UNIGENE5475	0.53038335	0.36534798	-0.6341553	1195.8014	1542.4081	0.41503724	0.034267504	-0.879387	512.04974	1086.2535	LOC_Os05g06780.1         	lipid binding protein	other
gb_UNIGENE1737	3.9156568	0.0002774059	1.3649831	15332.625	3962.6362	2.39902	0.00080667104	0.8750603	9966.3125	4116.3867	LOC_Os03g49380.1         	lipoxygenase 4	other
gb_UNIGENE26335	2.1466606	0.0008322836	0.7639134	1002.932	471.01797	1.7603059	0.012184718	0.56548756	799.4597	453.9141	LOC_Os03g08220.1         	lipoxygenase 6	other
gb_UNIGENE13288	4.421749	0.0004915189	1.4865353	3149.2349	713.9179	3.6376088	0.0032964884	1.2913265	3911.9075	1026.1062	LOC_Os08g39840.1         	lipoxygenase 7, chloroplast precursor	other
gb_UNIGENE19604						2.7564197	0.0014685204	1.0139326	361.158	127.80186	LOC_Os06g22050.1         	loricrin, putative	other
gb_UNIGENE5815	0.49654058	0.006291521	-0.70009005	1022.1523	2016.0282	0.2841537	0.002690593	-1.25824	470.944	1709.0719	LOC_Os08g08040.1         	LSM7 homolog, U6 small nuclear RNA associated	other
gb_UNIGENE1615	4.602117	0.001570682	1.5265164	86389.805	19106.781	2.753863	0.0027250813	1.0130047	72531.625	26577.904	LOC_Os02g02410.1         	luminal-binding protein 3 precursor	other
gb_UNIGENE14290	0.50887585	0.027447712	-0.6755512	2225.9949	4251.7534	0.4531843	0.031838458	-0.7914564	1989.69	4072.5986	LOC_Os01g39960.1         	lycopene epsilon cyclase, chloroplast precursor	other
gb_UNIGENE10608	2.2841513	0.00048918853	0.8259945	1622.685	714.83417	1.8473222	0.0069255573	0.61373717	3167.1177	1704.7393	LOC_Os05g01760.1         	lysine ketoglutarate reductase trans-splicing related 1	other
gb_UNIGENE6067	2.36706	0.0021278546	0.8616487	5636.13	2348.5154	1.960177	0.004437607	0.6730347	4298.8	2207.527	LOC_Os04g53190.2         	lysine-specific histone demethylase 1	other
gb_UNIGENE17030	2.4073555	0.000026860529	0.87852883	10630.439	4427.2397	2.2211635	0.0038300273	0.7980312	8396.274	3835.2754	LOC_Os04g57550.1         	lysine-specific histone demethylase 1	other
gb_UNIGENE6066	2.1631696	0.00367989	0.77157456	6529.008	3015.4587	2.0080044	0.00236983	0.6971414	5298.7803	2613.4446	LOC_Os04g53190.2         	lysine-specific histone demethylase 1	other
gb_UNIGENE15181	0.4025657	0.025454251	-0.909897	1744.1245	3768.1887	0.4791464	0.023000684	-0.7357491	3389.1123	6677.4307	LOC_Os06g48600.1         	macrophage migration inhibitory factor	other
gb_UNIGENE16214	0.10191372	0.08324033	-2.2836287	105.547874	466.52682	0.06333468	0.02239741	-2.7593222	14.780783	142.11661	LOC_Os04g28620.1         	male sterility protein 2	other
gb_UNIGENE20411	2.7410069	0.07124532	1.0083253	490.69324	150.07056	5.2345166	0.020089589	1.6552745	119.9159	25.627672	LOC_Os04g15920.1         	mannitol dehydrogenase	other
gb_UNIGENE13997						0.38851336	0.0030554987	-0.9454277	1183.4437	3032.0117	LOC_Os09g23550.2         	mannitol dehydrogenase	other
gb_UNIGENE10152	7.754221	0.0011027075	2.0482373	16290.799	2185.7126	5.7507157	0.00017030652	1.7493243	15599.75	2721.1228	LOC_Os03g09900.1         	membrane protein	other
gb_UNIGENE10724	2.546939	0.004660829	0.9348922	14562.949	5791.765	0.6083347					LOC_Os10g35850.1         	membrane steroid-binding protein 1	other
gb_UNIGENE9575	7.178078	0.00018239784	1.9710317	18095.273	2566.8237	4.9545217	0.0002453733	1.6003007	15610.725	3103.7705	LOC_Os04g57200.1         	metal ion binding protein	other
gb_UNIGENE17974	0.43100747	0.00054918113	-0.84162986	299.05325	684.9431	0.4288815	0.0064668767	-0.84657466	362.23123	826.5735	LOC_Os01g52160.1         	metal ion binding protein	other
gb_UNIGENE4641	5.005221	0.00063543714	1.6104815	9894.36	1979.9177	3.9046721	0.0003824349	1.3621738	7776.925	1959.6232	LOC_Os04g57200.1         	metal ion binding protein	other
gb_UNIGENE4636	7.4130697	0.0014904597	2.0032446	3455.1274	459.7276	5.80909	0.0005853871	1.759424	2115.48	351.88354	LOC_Os04g57200.1         	metal ion binding protein	other
gb_UNIGENE3058	4.557432	0.0027473583	1.5167594	27095.176	5967.8223	3.204135	0.0031803837	1.1644422	21910.65	6834.2695	LOC_Os10g30450.1         	metal ion binding protein	other
gb_UNIGENE4639	4.6460485	0.016452873	1.5360171	270.22748	61.4705	8.773889	0.004955953	2.17178	116.657074	13.740076	LOC_Os04g57200.1         	metal ion binding protein	other
gb_UNIGENE10448	0.46906742	0.0047667813	-0.75700873	1076.6161	2230.2764	0.5629356	0.010932837	-0.57459	431.3715	762.5691	LOC_Os10g38870.1         	metal ion binding protein	other
gb_UNIGENE9576	1.919591	0.002878873	0.6521121	7610.4478	3880.8054	2.0095081	0.00015752586	0.69789	7520.1924	3747.7969	LOC_Os04g57200.1         	metal ion binding protein	other
gb_UNIGENE13088	0.4877082	0.0036012074	-0.71803796	8584.157	17718.555	0.63678813	0.0054025794	-0.4513183	9536.02	14832.643	LOC_Os03g12530.1         	metal tolerance protein C3	other
gb_UNIGENE17360	0.50822926	0.04107373	-0.67682266	302.87802	567.33234	0.4822801	0.015985038	-0.7292302	313.38675	634.296	LOC_Os04g52100.1         	methionine aminopeptidase 1B, chloroplast precursor	other
gb_UNIGENE17399	0.4881455	0.009332768	-0.71714175	1088.0477	2197.9238	0.65941983	0.0057762978	-0.41639486	1352.5725	2048.404	LOC_Os05g01470.3         	methionine S-methyltransferase	other
gb_UNIGENE16037	2.8528314	0.0117707085	1.048312	4724.84	1724.0332	1.8880086	0.010354379	0.6355226	5277.043	2797.764	LOC_Os10g37770.1         	methyltransferase family protein	other
gb_UNIGENE2985	0.43914095	0.08274069	-0.82293487	9084.588	17476.387	0.4602746	0.017496381	-0.775932	6531.3896	14155.839	LOC_Os03g20700.2         	Mg-chelatase subunit XANTHA-F	other
gb_UNIGENE15227	0.37909186	0.019909186	-0.9699767	844.138	2067.416	0.44379783	0.012231228	-0.81238616	662.0743	1495.3278	LOC_Os09g27700.2         	microtubule-associated protein MAP65-1a	other
gb_UNIGENE15142	5.468147	0.0010969598	1.6989398	514.83276	95.52275	4.9990215	0.0014090921	1.6092422	357.7515	71.15019	LOC_Os01g42030.1         	mitochondrial chaperone BCS1	other
gb_UNIGENE2946	4.6912827	0.00052693795	1.545706	1171.9695	243.50517	3.6777809	0.007124795	1.3023095	862.193	249.72392	LOC_Os08g44340.1         	monodehydroascorbate reductase	other
gb_UNIGENE17954	2.7509124	0.008431885	1.0119326	30.242777	10.484405	2.0891647	0.0080264	0.7367643	21.864723	10.315293	LOC_Os01g49380.1         	monoglyceride lipase	other
gb_UNIGENE10756	10.568362	0.0015781356	2.3578649	1661.54	162.15079	12.33367	0.00003939948	2.512333	803.9565	64.95831	LOC_Os03g05920.1         	monooxygenase	other
gb_UNIGENE12007	1.784396	0.006417744	0.57908	521.7435	288.3932	2.5068176	0.013547947	0.91901404	165.68225	63.905815	LOC_Os03g05900.2         	monooxygenase	other
gb_UNIGENE21141	0.41866642	0.00049667043	-0.8706808	2395.915	5711.869	0.45072564	0.00028449108	-0.79689646	2748.6626	6086.9053	LOC_Os09g37620.1         	monooxygenase/ oxidoreductase	other
gb_UNIGENE4833	2.6420314	0.0050878366	0.97154814	6319.395	2353.3264	2.264554	0.0053987047	0.8173778	5299.0425	2285.7002	LOC_Os03g42110.1         	N-acetyl-gamma-glutamyl-phosphate reductase, chloroplast precursor	other
gb_UNIGENE15462	6.255329	0.0010958799	1.8334337	12988.8	2106.3884	5.8097286	0.0004299908	1.7595339	3007.0625	510.2447	LOC_Os03g58010.1         	N-acetyltransferase	other
gb_UNIGENE2888	4.883098	0.00055158755	1.5857799	24518.5	4997.012	5.2599626	0.0005247433	1.660124	19688.55	3742.9434	LOC_Os05g09440.1         	NADP-dependent malic enzyme, chloroplast precursor	other
gb_UNIGENE14323	0.47278294	0.010743379	-0.7491189	796.6212	1623.0266	0.5150453	0.009228098	-0.6635004	276.22925	539.00916	LOC_Os01g72460.1         	NADPH quinone oxidoreductase 2	other
gb_UNIGENE5574	3.4481597	0.0015204675	1.2378407	8160.145	2402.2173	2.914445	0.014675953	1.0696794	5855.1074	1901.8562	LOC_Os09g38620.1         	NADPH--cytochrome P450 reductase	other
gb_UNIGENE11212	4.083195	0.0033936137	1.4068798	2234.475	549.2025	3.2460015	0.03495836	1.177424	10477.55	3595.0479	LOC_Os04g49194.1         	naringenin,2-oxoglutarate 3-dioxygenase	other
gb_UNIGENE11836	2.2052476	0.0017384002	0.79083985	1547.4476	698.8702	2.0713165	0.0027155443	0.72818434	1015.971	482.98264	LOC_Os11g07440.1         	neutral/alkaline invertase	other
gb_UNIGENE13809	2.159592	0.017104207	0.7699193	495.89474	230.62912	1.7121078	0.018319601	0.5377252	361.8955	214.37599	LOC_Os04g35060.1         	nicotinate phosphoribosyltransferase-like protein	other
gb_UNIGENE13776	2.050663	0.1261597	0.71816313	96.77295	53.489075	3.0325046	0.012657967	1.1093888	274.4255	87.83522	LOC_Os02g53130.1         	nitrate reductase	other
gb_UNIGENE15251	2.5866218	0.025264459	0.95035267	115.027	49.22737	2.1763597	0.005496918	0.77765363	84.74165	37.729584	LOC_Os09g12380.1         	no apical meristem protein	other
gb_UNIGENE13898	2.0697386	0.00353252	0.72742236	2371.095	1154.4176	1.9868999	0.0021819542	0.68657553	2262.395	1131.9106	LOC_Os09g32040.1         	no apical meristem protein	other
gb_UNIGENE21008	0.2617714	0.017077964	-1.3402836	196.71002	647.11743	0.31614047	0.009710963	-1.1515687	332.467	1011.14197	LOC_Os08g31130.1         	nodulin protein	other
gb_UNIGENE14080	3.0372276	0.00040317973	1.1109451	689.6515	229.58453	2.4907534	0.0011875089	0.9125852	488.492	195.83476	LOC_Os06g50180.1         	non-imprinted in Prader-Willi/Angelman syndrome region protein 1	other
gb_UNIGENE972	0.48264366	0.036372297	-0.72847664	12140.859	24402.889	0.49493587	0.046646167	-0.70332706	10397.478	20023.988	LOC_Os11g24070.1         	nonspecific lipid-transfer protein 1 precursor	other
gb_UNIGENE871	0.47121298	0.06618921999999999	-0.7524451	3374.6526	6420.8677	0.46198848	0.033444595	-0.7722153	2193.51	4680.1895	LOC_Os11g24070.1         	nonspecific lipid-transfer protein 1 precursor	other
gb_UNIGENE698	0.5196027	0.0072110537	-0.6546908	263.691	486.00012	0.2906472	0.019783292	-1.235645	164.82768	495.50354	LOC_Os12g02340.1         	nonspecific lipid-transfer protein 3 precursor	other
gb_UNIGENE12237	7.35053	0.00028163998	1.9947724	13715.468	1872.0143	5.1619143	0.0018111229	1.6413075	8106.5625	1604.3232	LOC_Os05g40010.1         	nonspecific lipid-transfer protein precursor	other
gb_UNIGENE4414	2.6316545	0.000285209	0.96761274	86977.03	33227.42	2.4904008	0.00054343406	0.9124437	99065.73	40455.707	LOC_Os10g36170.1         	nonspecific lipid-transfer protein precursor	other
gb_UNIGENE11884	0.43529952	0.02439398	-0.83172095	1057.5182	2223.3206	0.60961896	0.11360463	-0.49492115	896.031	1290.0886	LOC_Os06g49190.1         	nonspecific lipid-transfer protein precursor	other
gb_UNIGENE3841	2.1035333	0.0646255	0.7436184	18624.6	8655.667	2.2272646	0.02405557	0.8007742	17339.15	7881.206	LOC_Os01g36950.2         	N-rich protein	other
gb_UNIGENE24544	0.43945286	0.002263967	-0.82222486	823.4745	1846.8442	0.6267715	0.009825027	-0.4671732	988.92053	1589.9543	LOC_Os04g46000.1         	nuc-1 negative regulatory protein preg	other
gb_UNIGENE13931	0.41065866	0.04079085	-0.88999295	457.3058	1055.4258	0.39072892	0.0031312383	-0.93974125	307.26926	761.2599	LOC_Os03g15790.1         	nucleic acid binding protein	other
gb_UNIGENE5688	0.3514262	0.015257486	-1.0457555	2135.9023	6209.1904	0.54410857	0.01181229	-0.6086065	2686.8228	5034.9067	LOC_Os01g09620.2         	nucleic acid binding protein	other
gb_UNIGENE8505	2.0298088	0.00073529384	0.70794153	2247.8926	1109.2655	1.7383747	0.007712198	0.5529506	779.0885	442.68677	LOC_Os07g48430.1         	nucleoside-triphosphatase	other
gb_UNIGENE7663	0.4645042	0.07603492	-0.76678467	2878.7783	5940.6294	0.44673362	0.002828364	-0.8057928	1327.5535	2912.5032	LOC_Os12g01370.1         	omega-3 fatty acid desaturase, chloroplast precursor	other
gb_UNIGENE2947	0.22896236	0.027997509	-1.4741976	1636.7548	5335.23	0.0890647	0.46800858	-2.4183922	32.54123	55.54905	LOC_Os12g09770.1         	O-methyltransferase family protein	other
gb_UNIGENE4910	0.29338494	0.017296117	-1.2262697	2718.0522	8653.988	0.33216974	0.009253533	-1.1021092	1948.2566	5297.3877	LOC_Os11g20090.1         	O-methyltransferase ZRP4	other
gb_UNIGENE4911	0.22037785	0.028815007	-1.5124117	1208.4116	4976.5703	0.2192819	0.0034225304	-1.5173972	428.82224	1741.6876	LOC_Os11g20090.1         	O-methyltransferase ZRP4	other
gb_UNIGENE3547	0.33583334	0.012678656	-1.0911403	164.718	505.78165	0.23329598	0.09708175	-1.4554473	272.21124	1085.8394	LOC_Os11g20090.1         	O-methyltransferase ZRP4	other
gb_UNIGENE6345	0.25502333	0.016176116	-1.3664002	43.27625	142.50462	0.64998657	0.33427647	-0.4308036	404.79175	624.4433	LOC_Os11g20090.1         	O-methyltransferase ZRP4	other
gb_UNIGENE70	4.068955	0.0016599202	1.4033862	57573.15	14361.26	2.459649	0.00032430532	0.9000187	39534.92	16044	LOC_Os11g09280.2         	OsPDIL1-1 - Oryza sativa protein disulfide isomerase	other
gb_UNIGENE725	4.3433175	0.0026806374	1.4686384	55191	13003.377	2.5698988	0.0016705107	0.94386655	36976.426	14379.664	LOC_Os11g09280.2         	OsPDIL1-1 - Oryza sativa protein disulfide isomerase	other
gb_UNIGENE758	3.703924	0.0033996336	1.3093928	29085.225	7992.525	2.2478228	0.0013644843	0.80996203	18427.15	8149.72	LOC_Os11g09280.2         	OsPDIL1-1 - Oryza sativa protein disulfide isomerase	other
gb_UNIGENE2576	2.1913884	0.025157845	0.7845353	19432.65	8915.006	1.84499	0.014376291	0.61247385	15505.75	8563.216	LOC_Os05g06430.2         	OsPDIL2-1 - Oryza sativa protein disulfide isomerase	other
gb_UNIGENE2575	2.4086263	0.0074291606	0.87905663	39736.375	16713.781	1.8662943	0.013532417	0.6239548	31574.625	17298.123	LOC_Os01g23740.1         	OsPDIL2-2 - Oryza sativa protein disulfide isomerase	other
gb_UNIGENE2442	2.7572699	0.0129731065	1.014241	5291.307	1928.1945	1.6411082	0.007493755	0.49537173	3265.99	1984.4753	LOC_Os09g27830.1         	OsPDIL2-3 - Oryza sativa protein disulfide isomerase	other
gb_UNIGENE6622	3.1138535	0.011505317	1.135861	75.64873	23.073507	1.97122	0.027769528	0.67865264	37.1816	17.486368	LOC_Os10g37340.1         	O-succinylhomoserine sulfhydrylase	other
gb_UNIGENE3017	6.38697	0.0022159717	1.85426	6898.545	1061.3755	6.8189893	0.0028704673	1.9197112	2920.165	457.9412	LOC_Os03g48780.1         	oxalate oxidase 2 precursor	other
gb_UNIGENE3018	11.864342	0.0063194553	2.4735374	970.58875	78.58819	8.676298	0.014737634	2.160595	173.85846	33.29567	LOC_Os03g48780.1         	oxalate oxidase 2 precursor	other
gb_UNIGENE5337	5.0558276	0.0008891999	1.6205416	4875.0474	1003.3944	2.1468506	0.0015566401	0.7640019	3929.5825	1826.4327	LOC_Os08g37874.1         	oxidoreductase	other
gb_UNIGENE20510	2.3199816	0.005205303	0.84155923	631.2075	279.852	2.7985485	0.00016759231	1.0291009	426.70575	152.53386	LOC_Os05g50090.1         	oxidoreductase	other
gb_UNIGENE5096	2.8250635	0.00035688694	1.0385308	687.97076	244.75714	2.1535964	0.0062471456	0.7671392	1194.2876	566.3105	LOC_Os02g48110.1         	oxygen-regulated protein precursor	other
gb_UNIGENE4195	0.38195592	0.018181618	-0.9624501	187.87274	492.9123	0.35255808	0.028845213	-1.04254	164.543	478.59198	LOC_Os11g39990.1         	patatin T5 precursor	other
gb_UNIGENE6910	2.5019717	0.006381078	0.9170791	1922.505	787.08167	1.9561534	0.07424419	0.67098	2178.2075	1089.7096	LOC_Os08g37180.1         	patatin T5 precursor	other
gb_UNIGENE15718	5.9027705	0.0049786456	1.7754219	882.22546	158.41762	5.916717	0.0017812155	1.7777817	412.99026	68.18989	LOC_Os06g46350.1         	patatin-like phospholipase family protein	other
gb_UNIGENE9382	0.5576846	0.10019979	-0.58396167	1239.7858	1933.5525	0.4726094	0.02587515	-0.749486	889.34296	1639.9431	LOC_Os02g33380.1         	pectinesterase inhibitor domain containing protein	other
gb_UNIGENE22289	0.38646364	0.027147792	-0.95071745	1483.5968	3427.7805	0.4277898	0.015722083	-0.8491233	1505.7625	3404.086	LOC_Os05g31160.1         	peptide chain release factor 2	other
gb_UNIGENE7849	0.48470792	0.016822018	-0.7242088	181.32623	351.16275	0.5322029	0.004743826	-0.6307305	98.6949	186.24121	LOC_Os01g10950.1         	peptide-N4-asparagine amidase A	other
gb_UNIGENE6857	2.0418532	0.006917636	0.71385777	311.86676	157.74333	1.6448191	0.0077198194	0.49763045	251.72275	158.39594	LOC_Os08g45210.1         	peroxisomal membrane protein PMP22	other
gb_UNIGENE5469	28.839201	0.0013772764	3.3617356	1261.2325	49.827793	19.222435	0.00027474985	2.956078	912.72174	49.208435	LOC_Os03g61470.1         	PGPS/D12	other
gb_UNIGENE5468	5.416711	0.013604924	1.6894888	751.57324	129.02493	4.341929	0.009248551	1.4683187	1713.822	352.53076	LOC_Os03g61470.1         	PGPS/D12	other
gb_UNIGENE4955	0.45850444	0.03810312	-0.7797853	518.4697	1186.7305	0.69915676	0.022946041	-0.3578803	545.62775	781.7015	LOC_Os03g49440.1         	phosphatase	other
gb_UNIGENE4956	0.4212109	0.0381307	-0.8646216	4403.4277	11328.262	0.71165794	0.12781148	-0.34015793	4759.003	6851.4053	LOC_Os03g49440.1         	phosphatase	other
gb_UNIGENE13161	0.45363045	0.029182719	-0.7904724	120.613	279.7533	0.71343726	0.066804804	-0.3376608	144.6121	201.63503	LOC_Os05g46040.1         	protein phosphatase 2C ABI2	other
gb_UNIGENE11966	1.206851	0.35339376	0.18801446	31.660374	25.780912	2.9305425	0.009545913	1.0751876	46.152824	16.632318	LOC_Os04g34080.1         	protein phosphatase type 2A regulator/ signal transducer	other
gb_UNIGENE11285	6.8744893	0.000678931	1.9278173	5861.705	883.33673	4.6747165	0.00033280032	1.5421685	5089.463	1098.7318	LOC_Os04g37600.1         	phosphate carrier protein, mitochondrial precursor	other
gb_UNIGENE10529	3.2123535	0.000003846074	1.1670039	986.3628	306.78964	2.26552	0.00022319288	0.8178044	396.30075	176.62582	LOC_Os01g70210.1         	phosphatidylinositol transfer protein CSR1	other
gb_UNIGENE7525	2.3540263	0.0043857493	0.85612714	2940.2773	1271.7966	2.0788379	0.028461484	0.731809	2470.6475	1240.4376	LOC_Os03g50030.1         	phospholipase A2	other
gb_UNIGENE8402	2.890453	0.003331376	1.0614133	1819.99	625.14	2.41808	0.002703136	0.8829739	1343.9	558.0119	LOC_Os09g37100.1         	phospholipase D delta	other
gb_UNIGENE23169	0.6362806	0.11853506	-0.45211565	1118.5651	1750.285	0.3862814	0.022254277	-0.95118916	450.25397	1133.1443	LOC_Os03g30250.1         	phytochelatin synthetase-like conserved region family protein	other
gb_UNIGENE13305	0.330663	0.059834093	-1.1066556	2435.0747	5796.4023	0.377138	0.02065899	-0.97514415	1336.2977	3233.7373	LOC_Os06g51290.3         	phytoene synthase, chloroplast precursor	other
gb_UNIGENE3668	2.1865025	0.01979424	0.7823032	422.22275	195.97816	0.8211484	0.14438915	-0.19705145	102.32403	128.19392	LOC_Os07g01710.1         	phytosulfokine receptor precursor	other
gb_UNIGENE10013	1.8899071	0.05370465	0.63652766	686.088	358.00864	2.048532	0.009477987	0.7171234	667.89	326.19818	LOC_Os03g54900.2         	phytosulfokine receptor precursor	other
gb_UNIGENE11499	4.2657685	0.0047904565	1.4506223	266.65424	64.30918	3.3861458	0.0041808817	1.2196923	232.26125	70.18939	LOC_Os07g03200.1         	phytosulfokines 4 precursor	other
gb_UNIGENE17012	0.39494318	0.037137467	-0.9290134	216.56454	502.99042	0.3261851	0.024268946	-1.1202903	80.70522	251.90747	LOC_Os05g28730.1         	PIT1	other
gb_UNIGENE8004	2.5839546	0.020690842	0.94932103	6986.4326	2589.1602	1.6658036	0.055677477	0.5103076	1798.46	1058.0547	LOC_Os07g26110.1         	plant integral membrane protein TIGR01569 containing protein	other
gb_UNIGENE13172	0.46168295	0.013564599	-0.77287686	3946.3176	8285.909	0.58491	0.010374009	-0.5362974	4437.6	7495.2773	LOC_Os03g06680.1         	plant-specific domain TIGR01615 family protein	other
gb_UNIGENE13274	0.39864358	0.026480164	-0.91968757	1455.3628	3175.0122	0.52098316	0.0033808055	-0.65203756	1390.7725	2585.033	LOC_Os12g10320.1         	plant-specific domain TIGR01627 family protein	other
gb_UNIGENE4290	2.1832604	0.00039454558	0.7808194	7063.5	3253.015	1.8944238	0.0029389854	0.63891476	7198.992	3780.819	LOC_Os05g41480.2         	plastidic phosphate translocator-like protein1	other
gb_UNIGENE3743	2.7859092	0.0022550314	1.0245743	19547.5	7050.301	2.7402797	0.002402355	1.00806	13974.825	5182.913	LOC_Os07g42370.2         	pnFL-2	other
gb_UNIGENE10441	5.0153375	0.0030149939	1.6125007	2313.54	461.29468	6.433918	0.0021546641	1.8615837	1405.2676	223.91034	LOC_Os09g26780.1         	pnFL-2	other
gb_UNIGENE16091	2.2345788	0.0035034753	0.80405277	321.38202	146.50357	2.7226048	0.0006693676	1.0015891	389.803	140.55887	LOC_Os01g72100.1         	polcalcin Jun o 2	other
gb_UNIGENE26522	0.7455038	0.07842876	-0.29369503	204.7285	270.68964	0.49031228	0.030216279	-0.71271276	100.0103	190.86874	LOC_Os12g09739.1         	pollen signalling protein with adenylyl cyclase activity	other
gb_UNIGENE6732	4.5036592	0.00017286338	1.5048902	46785.074	10445.135	4.6050344	0.0007014356	1.5271502	34185.3	7355.6294	LOC_Os04g33390.1         	P-protein	other
gb_UNIGENE14233	0.461162	0.0020890555	-0.7740059	365.0075	797.99835	1.0696479	0.47926962	0.06732948	464.8165	451.1068	LOC_Os01g03180.1         	pr5	other
gb_UNIGENE15031	0.497815	0.0067048813	-0.69752675	983.021	1909.6556	0.6131184	0.024971353	-0.4891972	1240.1948	1994.7024	LOC_Os01g03180.1         	pr5	other
gb_UNIGENE1458	1.3931397	0.11980415	0.33156002	175.9395	129.56613	2.4237602	0.009143605	0.8853201	448.823	181.77939	LOC_Os06g31070.1         	prolamin PPROL 17 precursor	other
gb_UNIGENE1466	1.3384789	0.29564148	0.2915338	97.42186	98.14795	4.1066732	0.03251911	1.4126133	55.317333	13.207051	LOC_Os06g31070.1         	prolamin PPROL 17 precursor	other
gb_UNIGENE68	2.0521188	0.011156275	0.71887285	7374.63	3661.4236	1.7722093	0.0074126753	0.57222694	6227.9453	3535.2976	LOC_Os10g40360.1         	proline oxidase, mitochondrial precursor	other
gb_UNIGENE18080	2.7134113	0.0009657557	0.9982066	834.6575	308.23843	1.9659774	0.0023018552	0.67598957	856.63525	434.01392	LOC_Os01g55799.3         	protein binding protein	other
gb_UNIGENE19445	3.7239969	0.00015416337	1.3147975	2923.5327	776.8892	2.0354724	0.0012772911	0.7107279	704.0705	344.23953	LOC_Os04g24290.1         	protein kinase, putative	other
gb_UNIGENE14879	2.6174767	0.0011186079	0.9622108	2919.005	1132.1005	1.9838568	0.0029007588	0.6850428	2364.0425	1224.8167	LOC_Os02g45750.1         	protein kinase	other
gb_UNIGENE2881	2.9892972	0.006465876	1.0950383	35238.848	11571.513	2.6041994	0.0034937833	0.9571253	27937.826	10730.474	LOC_Os09g17730.2         	proton pump interactor	other
gb_UNIGENE5650	2.2311945	0.0007031991	0.8025371	128.73442	59.069305	2.1065693	0.0020981007	0.7450607	70.06407	33.171474	LOC_Os08g44370.1         	pyrimidine-specific ribonucleoside hydrolase rihA	other
gb_UNIGENE18037	3.7955766	0.030889297	1.3338363	635.2718	160.23576	6.6037526	0.0007341654	1.8876381	438.71402	69.23908	LOC_Os06g04240.1         	Q-rich domain protein	other
gb_UNIGENE20144	2.55419	0.005670484	0.9377351	3652.135	1455.3389	2.339477	0.0031794533	0.8499274	2431.84	1014.2909	LOC_Os03g09140.1         	ras-related protein Rab-6A	other
gb_UNIGENE12523	5.990957	0.000084664745	1.7902511	1280.4075	215.8831	6.694577	0.00018757318	1.9012978	979.86975	141.80064	LOC_Os08g44300.1         	recombination protein O, RecO	other
gb_UNIGENE12286	3.26086	0.0046488345	1.181991	1454.3151	460.50867	5.4624343	0.001940052	1.6978946	1825.93	320.7127	LOC_Os06g35700.1         	reticuline oxidase precursor	other
gb_UNIGENE14427	3.836322	0.009099206	1.3445141	532.8767	137.30377	2.4149559	0.013210678	0.88168097	345.30026	147.78404	LOC_Os06g35650.1         	reticuline oxidase precursor	other
gb_UNIGENE11913	2.6394274	0.00847873	0.97056204	3200.4497	1177.9857	1.5773367	0.0135230115	0.4557378	1229.2407	768.35004	LOC_Os03g27590.1         	retinoid-inducible serine carboxypeptidase precursor	other
gb_UNIGENE7697	2.1709414	0.021545121	0.7751609	5922.5825	2689.3909	1.9962484	0.006106859	0.6912696	6640.6553	3319.3384	LOC_Os05g18604.1         	retrotransposon protein, putative, Ty1-copia subclass	other
gb_UNIGENE3514	0.42875203	0.005016438	-0.8468765	94.90655	217.22266	0.760553	0.19300725	-0.27370948	97.98355	118.90362	LOC_Os04g54280.1         	retrotransposon protein, putative, unclassified	other
gb_UNIGENE20220	0.8375213	0.13436949	-0.17730856	2033.05	2345.7927	2.0394273	0.0026410476	0.712669	3026.6572	1478.0897	LOC_Os01g27430.1         	retrotransposon protein, putative, unclassified	other
gb_UNIGENE14464	0.34952903	0.0026056604	-1.0511687	105.788025	292.46964	0.379145	0.0050421013	-0.9698366	88.21146	228.25513	LOC_Os08g16830.1         	retrotransposon protein, putative, SINE subclass	other
gb_UNIGENE17073	0.49410293	0.017306058	-0.7050114	731.344	1445.9485	0.5163973	0.027337061	-0.66087884	863.11847	1555.036	LOC_Os03g40020.1         	rf1 protein, mitochondrial precursor	other
gb_UNIGENE18459	2.5524733	0.009489585	0.93706286	334.3695	141.3289	2.8208804	0.00031693862	1.037049	695.66003	244.48157	LOC_Os10g03570.1         	RGH1A, putative	other
gb_UNIGENE5012	2.1999798	0.012908471	0.7884482	11975.955	5329.9937	2.3029225	0.018707803	0.8341789	10187.777	4394.287	LOC_Os02g36340.1         	riboflavin biosynthesis protein ribAB, chloroplast precursor	other
gb_UNIGENE3691	1.9969333	0.03973259	0.69161266	6643.718	3271.9685	2.5301905	0.0023047084	0.92829454	6226.5576	2464.1257	LOC_Os07g43670.1         	ribonuclease 1 precursor	other
gb_UNIGENE9742	0.4408611	0.0077587054	-0.8190254	832.70154	1833.1858	0.46781144	0.0000289198	-0.75969	341.3705	728.0328	LOC_Os01g67190.1         	ribonuclease 2 precursor	other
gb_UNIGENE5058	0.19171588	0.00584678	-1.6517408	250.41989	1134.3157	0.2569896	0.04556243	-1.3587197	204.74062	613.1758	LOC_Os09g36680.1         	ribonuclease 3 precursor	other
gb_UNIGENE5059	0.16041966	0.010458117	-1.829962	403.1256	1709.9292	0.24443853	0.05484365	-1.4087914	492.03613	1419.3776	LOC_Os09g36680.1         	ribonuclease 3 precursor	other
gb_UNIGENE17817	0.3822112	0.012237189	-0.9617819	172.88126	489.88644	0.45076525	0.036060777	-0.7968086	138.49475	335.65933	LOC_Os07g08030.1         	ribose-5-phosphate isomerase	other
gb_UNIGENE11768	1.3254453	0.05623199	0.2817485	2820.71	2171.1147	2.235905	0.0017106406	0.8046461	2780.4	1223.9891	LOC_Os01g52530.1         	ribose-phosphate pyrophosphokinase 3	other
gb_UNIGENE997	0.35529643	0.06692855	-1.0348028	38826.926	95638.51	0.43801355	0.03293904	-0.82550544	47790.598	109353.3	LOC_Os12g19394.1         	ribulose bisphosphate carboxylase small chain C, chloroplast precursor	other
gb_UNIGENE1019	2.4018188	0.0034709985	0.87622625	58.378395	26.387379	1.3026309	0.20488364	0.264386	55.75935	46.0636	LOC_Os11g47970.1         	ribulose bisphosphate carboxylase/oxygenase activase, chloroplast precursor	other
gb_UNIGENE18654	0.5812725	0.052483056	-0.54253566	174.4165	283.59442	0.42405275	0.02846909	-0.85789746	84.78618	188.17831	LOC_Os04g53580.1         	RIC10	other
gb_UNIGENE6076	3.5689478	0.002816921	1.2722708	13323.665	3623.446	3.008838	0.00019530214	1.1015539	8608.814	2859.9624	LOC_Os03g47500.2         	RING finger protein 5	other
gb_UNIGENE20287	0.2945569	0.00720291	-1.2222831	120.32703	374.00662	0.31579372	0.004957624	-1.1526661	218.1875	669.2375	LOC_Os02g45710.1         	RING zinc finger protein-like	other
gb_UNIGENE24933	1.9081289	0.0006987061	0.6461231	751.51794	394.17386	2.0833015	0.0069921636	0.73395395	825.27423	397.28555	LOC_Os08g43670.1         	RING-H2 finger protein ATL2B	other
gb_UNIGENE20600	6.77865	0.00043208722	1.913778	465.54327	68.511894	4.870578	0.0004976132	1.5832126	338.332	69.39583	LOC_Os01g20910.1         	RING-H2 finger protein ATL2L	other
gb_UNIGENE4213	1.6108534	0.22330742	0.4767641	104.506996	62.441544	5.9731665	0.020484999	1.7872772	594.17596	90.947235	LOC_Os02g42450.1         	ripening-related protein 2 precursor	other
gb_UNIGENE9469	2.2628093	0.030820716	0.8166071	114.17888	60.345146						LOC_Os10g34760.1         	ripening-related protein 6 precursor, putative	other
gb_UNIGENE15472	0.42547542	0.03274631	-0.8545481	248.5545	589.7621	0.3727799	0.0065009026	-0.9867671	120.02835	322.74533	LOC_Os06g49760.1         	ripening-related protein	other
gb_UNIGENE15424	0.5090537	0.15310527	-0.6752017	134.88628	287.94485	0.3738285	0.002918116	-0.9839581	50.160297	138.59311	LOC_Os06g49760.1         	ripening-related protein	other
gb_UNIGENE1269	2.0487847	0.03178802	0.7172468	20175	10234.1	1.5879242	0.012786049	0.46242768	11755.475	7406.4297	LOC_Os01g22010.3         	S-adenosylmethionine synthetase 1	other
gb_UNIGENE11378	2.8534133	0.005669574	1.0485159	623.9925	222.14627	2.1625354	0.004629378	0.77128136	357.75476	167.2817	LOC_Os01g51870.1         	S-adenosylmethionine-dependent methyltransferase	other
gb_UNIGENE9898	2.686213	0.013309268	0.9881324	925.15	333.8463	2.284828	0.004873529	0.8262907	635.0692	278.155	LOC_Os01g22954.1         	SCPL33	other
gb_UNIGENE15859	2.0288978	0.0018202671	0.7074927	794.566	393.04904	1.6996763	0.015949428	0.5304378	577.3738	343.49463	LOC_Os02g51430.2         	SEC6	other
gb_UNIGENE538	15.17352	0.000021664073	2.7195518	2614.2925	172.56207	10.470534	0.00017845634	2.348565	1763.99	169.95665	LOC_Os10g34910.1         	secretory protein, putative	other
gb_UNIGENE634	18.706074	0.003056912	2.9288483	149857.5	10138.011	10.935092	0.00053959957	2.391977	145270.5	13975.917	LOC_Os10g34930.1         	secretory protein	other
gb_UNIGENE590	1.4545307	0.15762535	0.37468332	25.25535	17.117947	11.39501	0.00014352925	2.4331756	19299.824	1727.2384	LOC_Os10g34920.1         	secretory protein	other
gb_UNIGENE24835	0.44317785	0.0047772173	-0.8137841	6398.5674	14512.367	0.65638846	0.0027445531	-0.4210025	4631.6353	7133.543	LOC_Os09g10620.1         	seed maturation protein LEA 4, putative	other
gb_UNIGENE11396	0.3965001	0.012276263	-0.9250789	457.02252	1061.6311	0.3730391	0.015208008	-0.98607206	872.47253	2438.6462	LOC_Os06g02040.1         	seed maturation protein	other
gb_UNIGENE10150	0.036526904	0.12509604	-3.3097062	10.455826	38.44078	0.29587534	0.005487402	-1.2178171	211.86246	738.22424	LOC_Os06g02040.1         	seed maturation protein	other
gb_UNIGENE3054	2.5416932	0.0016682446	0.9328305	11999.237	4712.1494	2.1293507	0.0012077546	0.75581706	3876.44	1827.3533	LOC_Os09g25760.1         	senescence-associated protein DH	other
gb_UNIGENE15259	0.28947917	0.029204652	-1.239672	3483.8975	12641.965	0.47896343	0.0649998	-0.736131	3575.1274	7587.812	LOC_Os06g50930.1         	senescence-associated protein DIN1	other
gb_UNIGENE22898	0.45667064	0.029520547	-0.78379285	42.983376	100.079994	0.8025786	0.48224008	-0.21992546	78.584625	94.8003	LOC_Os06g50930.2         	senescence-associated protein DIN1	other
gb_UNIGENE9086	2.1703835	0.020953499	0.7749039	1904.4775	865.2664	2.1876433	0.00912997	0.7828249	1701.675	770.9037	LOC_Os01g70380.1         	serine palmitoyltransferase 2	other
gb_UNIGENE9196	2.6979744	0.00202851	0.99250126	1788.365	667.9217	3.3383687	0.0053205006	1.2054822	749.1233	222.33646	LOC_Os06g12150.2         	shikimate kinase, chloroplast precursor	other
gb_UNIGENE9197	3.0752342	0.0029672657	1.123381	402.51276	135.31909	3.3477952	0.009423407	1.208302	237.68774	72.744286	LOC_Os06g12150.1         	shikimate kinase, chloroplast precursor	other
gb_UNIGENE12026	2.5930111	0.00054275646	0.9528198	6977.44	2692.3064	2.1159828	0.0011347223	0.74951935	5374.9478	2529.0095	LOC_Os02g24080.5         	SNAP25 homologous protein SNAP30	other
gb_UNIGENE13096	2.9810345	0.0016051754	1.0922704	8304.46	2786.422	2.387326	0.003613995	0.8701739	7242.04	2975.4272	LOC_Os06g39260.1         	solute carrier family 35 member B1	other
gb_UNIGENE5784	8.564939	0.00066425547	2.147677	695.00903	84.66897	7.0084887	0.0018723689	1.9471221	493.86346	70.55169	LOC_Os03g02670.2         	solute carrier family 35 member B3	other
gb_UNIGENE16603	2.277985	0.004191453	0.82329136	7310.7075	3170.3896	1.7567543	0.0065311193	0.5634679	4000.2424	2287.8374	LOC_Os01g54580.1         	solute carrier family 35 member C2	other
gb_UNIGENE22023	4.924275	0.005810522	1.594177	334.87823	65.608475	3.7263825	0.022670912	1.3154379	79.53275	25.509037	LOC_Os08g32060.1         	spotted leaf protein 11	other
gb_UNIGENE11129	5.0208497	0.02041817	1.6135992	108.1678	26.943962	4.7257886	0.0010009105	1.5530344	74.34165	16.264053	LOC_Os07g42250.1         	strictosidine synthase 1 precursor	other
gb_UNIGENE3380	1.9717162	0.10144531	0.6789043	8828.913	4385.027	2.0802104	0.012455401	0.7324691	7577.2725	3514.1104	LOC_Os01g42860.1         	subtilisin-chymotrypsin inhibitor 2	other
gb_UNIGENE5537	6.9877105	0.00038824722	1.944153	47632	6967.1147	5.497595	0.0004247447	1.7043107	36994.426	6654.842	LOC_Os07g01560.2         	sugar transport protein 1	other
gb_UNIGENE20294	2.0216794	0.010659525	0.7039286	5979.87	3038.3264	1.8966079	0.002021787	0.640067	6408.7324	3398.6733	LOC_Os09g15330.2         	sugar transport protein 14	other
gb_UNIGENE24094	2.078666	0.0036798397	0.7317263	987.86304	489.23047	2.1958654	0.0004235703	0.7865762	1008.542	451.53577	LOC_Os04g37970.1         	sugar transport protein 5, putative	other
gb_UNIGENE9662	8.973066	0.00034407424	2.1942275	11258.842	1278.6785	7.690398	0.0002918202	2.0399725	8236.072	1083.4879	LOC_Os03g11900.1         	sugar transport protein 8	other
gb_UNIGENE20873	0.52467763	0.021348814	-0.64497125	315.1025	585.0457	0.46860388	0.024103748	-0.75799745	251.04774	484.0473	LOC_Os07g48360.1         	superfamily II DNA helicase	other
gb_UNIGENE10483	2.0496252	0.003037223	0.7176569	496.1115	240.89288	2.8538485	0.006414055	1.0486684	354.05225	127.35941	LOC_Os02g51150.1         	protein SUR2	other
gb_UNIGENE22370	3.0219584	0.0015792534	1.105905	4192.4375	1403.3824	2.6905081	0.00029035597	0.98973006	3729.85	1375.9135	LOC_Os03g57310.1         	syntaxin 121	other
gb_UNIGENE4999	3.1112828	0.0065552024	1.1350352	43.020226	13.976675	3.7541952	0.0004739906	1.322874	53.529625	14.500437	LOC_Os01g02390.1         	TAK14	other
gb_UNIGENE4997	1.8671877	0.023318963	0.6244334	999.6627	539.46936	2.355343	0.00294442	0.8566864	4325.3325	1853.6338	LOC_Os01g02390.1         	TAK14	other
gb_UNIGENE5260	0.46943757	0.011132249	-0.75622	5887.3896	12415.113	0.5685812	0.0022940459	-0.56461114	9253.846	16400.102	LOC_Os03g47610.8         	thiamine biosynthesis protein thiC	other
gb_UNIGENE14302	2.025878	0.0021406827	0.7060031	2286.1775	1131.511	1.8738213	0.0040766024	0.6279798	1598.3275	853.44446	LOC_Os01g12910.1         	thioesterase family protein	other
gb_UNIGENE1580	0.07819432	0.038993202	-2.5485582	1942.0247	18308.111	0.433046	0.4406108	-0.8369113	512.8008	1056.291	LOC_Os06g31800.1         	thionin precursor	other
gb_UNIGENE13994	2.1775367	0.002537733	0.77819425	178.424	83.04852	2.593981	0.022178138	0.9531938	82.17507	33.583897	LOC_Os02g56900.1         	thioredoxin	other
gb_UNIGENE5236	0.48124295	0.0046137753	-0.731383	5058.67	10590.536	0.6635982	0.009805297	-0.41007847	6379.025	9923.713	LOC_Os07g48510.1         	thioredoxin-like 1	other
gb_UNIGENE6584	2.4718542	0.002910925	0.90496856	5001.722	2004.488	2.3347867	0.0013425602	0.84792054	4084.9624	1746.6428	LOC_Os02g07044.1         	thiosulfate transferase	other
gb_UNIGENE15600	1.9895458	0.000785748	0.6879064	592.2655	301.2511	2.0355546	0.005593363	0.71076834	599.98047	296.12085	LOC_Os07g42310.1         	TMEM87A protein	other
gb_UNIGENE13952	11.990614	0.0002893047	2.4841242	654.39526	55.307415	17.526173	0.00025836434	2.8636954	386.42325	22.10842	LOC_Os02g09990.1         	TMV response-related gene product	other
gb_UNIGENE19593	1.18896	no replicates	0.17307894	11.8896	10	20.109413	0.0013583151	3.001188	882.33777	48.36261	LOC_Os09g32000.1         	TMV response-related gene product	other
gb_UNIGENE609	0.45969456	0.05173907	-0.777193	293.31378	597.06744	0.2887004	0.0037024117	-1.2423658	98.179924	314.8693	LOC_Os07g40130.1         	transcriptional regulatory protein algP, putative	other
gb_UNIGENE10749	0.35875794	0.040359527	-1.0251074	135.56665	333.07306	0.36800146	0.0008840304	-0.99966836	209.04324	560.4591	LOC_Os02g43670.1         	transferase family protein	other
gb_UNIGENE11572	4.8157682	0.005573472	1.5718956	607.963	127.37286	4.0936666	0.004015764	1.409441	535.714	132.63264	LOC_Os10g40640.1         	transferase, transferring glycosyl groups	other
gb_UNIGENE12799	2.5983155	0.0026433023	0.9548633	288.2255	110.899734	1.8800244	0.0013724404	0.6312848	254.3445	134.66203	LOC_Os03g30000.1         	transferase, transferring glycosyl groups	other
gb_UNIGENE7420	1.8739057	0.00062135677	0.6280248	164.93475	87.411194	2.2834952	0.022190643	0.8257072	54.505524	25.25578	LOC_Os07g08070.1         	transmembrane BAX inhibitor motif-containing protein 4	other
gb_UNIGENE16757	2.1870553	0.0040647835	0.782556	224.77075	102.39915	1.8253568	0.002824594	0.60177547	172.022	94.164154	LOC_Os05g38720.1         	transmembrane receptor	other
gb_UNIGENE21646	5.5027504	0.0036992743	1.705248	322.43124	57.154182	5.8680935	0.007025944	1.7695298	268.40274	47.259174	LOC_Os07g01750.1         	transparent testa 12 protein	other
gb_UNIGENE2595	10.469087	0.0013120648	2.3484268	4457.5947	423.1785	11.462731	0.00001957038	2.439101	2028.39	173.78264	LOC_Os09g17840.1         	protein transport protein Sec61 alpha subunit isoform 2	other
gb_UNIGENE2596	2.1720972	0.019973375	0.7756931	4466.0474	2068.1846	1.6329619	0.013182168	0.4903955	3408.3298	2086.0725	LOC_Os09g17840.1         	protein transport protein Sec61 alpha subunit isoform 2	other
gb_UNIGENE4691	2.416057	0.010912657	0.88213694	8933.105	3782.4175	1.9735565	0.004042185	0.6798372	6715.7	3378.904	LOC_Os01g38510.1         	protein transport protein Sec61 beta subunit	other
gb_UNIGENE3679	2.174671	0.016034318	0.77687734	12129.076	5593.7856	1.8161107	0.010049233	0.5966973	9034.342	4955.6655	LOC_Os02g08180.1         	protein transport protein SEC61 gamma subunit	other
gb_UNIGENE3681	2.062011	0.022572197	0.7236817	6678.13	3272.5784	1.6900033	0.016079543	0.52473044	4962.55	2910.2324	LOC_Os02g08180.1         	protein transport protein SEC61 gamma subunit	other
gb_UNIGENE16910	2.5642867	0.008013168	0.9416803	1510.7	600.9089	2.3194942	0.0035684113	0.8413491	939.76874	406.87897	LOC_Os04g05980.1         	transposon protein, putative, CACTA, En/Spm sub-class	other
gb_UNIGENE15587	0.42178515	0.027659563	-0.86325926	622.4723	1519.7196	0.43186197	0.0023160176	-0.83964926	2059.6775	4755.298	LOC_Os02g16590.1         	transposon protein, putative, CACTA, En/Spm sub-class	other
gb_UNIGENE22139	0.5473848	0.11252036	-0.60260326	51.6265	98.32634	0.38918433	0.002422098	-0.9437022	54.290802	134.17897	LOC_Os05g40220.2         	transposon protein, putative, CACTA, En/Spm sub-class	other
gb_UNIGENE11818	2.6631477	0.00013827847	0.97950876	4515.6504	1680.5466	2.381204	0.00032479962	0.8676062	2623.275	1103.443	LOC_Os10g37110.1         	transposon protein, putative, unclassified	other
gb_UNIGENE5887	13.987753	0.00053237536	2.6381822	578.59375	44.35025	10.163483	0.0031354823	2.3188012	471.73575	50.9501	LOC_Os04g51160.1         	transposon protein, putative, unclassified	other
gb_UNIGENE15936	15.712293	0.00094457617	2.7544434	455.14148	30.680319	10.080344	0.0016870737	2.3105874	322.55676	34.540962	LOC_Os04g51150.1         	transposon protein, putative, unclassified	other
gb_UNIGENE19871	2.6102314	0.006181507	0.95943886	878.31726	334.74863	3.0575824	0.012004083	1.1176245	737.12524	243.98178	LOC_Os01g70130.1         	transposon protein, putative, unclassified	other
gb_UNIGENE22627	2.1709926	0.011172916	0.7751845	1912.99	871.4008	2.028534	0.0114643015	0.7073133	3051.9075	1553.113	LOC_Os04g45900.1         	transposon protein, putative, unclassified	other
gb_UNIGENE5378	3.0347197	0.006632885	1.1101191	1436.2075	482.65814	2.242664	0.0045174668	0.8076645	1323.8676	594.8347	LOC_Os09g12600.1         	triose phosphate/phosphate translocator, non-green plastid,chloroplast precursor	other
gb_UNIGENE411	0.5279956	0.02492533	-0.63866735	1443.332	2708.3926	0.4694925	0.005426835	-0.756103	1099.645	2324.712	LOC_Os07g38730.1         	tubulin alpha-1 chain	other
gb_UNIGENE333	2.2323594	0.0036533708	0.8030591	45770.25	20504.965	1.6872754	0.00031393886	0.52311504	34705.574	20548.965	LOC_Os11g14220.2         	tubulin alpha-3 chain	other
gb_UNIGENE803	2.0765646	0.028427329	0.7307149	13247.75	6442.341	1.7020625	0.014036939	0.53184074	11657.402	6789.779	LOC_Os03g51600.3         	tubulin alpha-3 chain	other
gb_UNIGENE18474	3.4917655	0.006565938	1.2504075	109.80043	31.589293	2.2966955	0.01828787	0.8314714	105.7661	47.36262	LOC_Os09g32690.2         	ubiquitin-protein ligase/ zinc ion binding protein	other
gb_UNIGENE12685	7.109198	0.00956894	1.9613894	308.3295	48.42633	4.136744	0.005237269	1.419909	695.82996	158.19034	LOC_Os08g04370.1         	uclacyanin-2 precursor, putative	other
gb_UNIGENE13477	0.4867307	0.022475848	-0.7200443	402.63477	738.7295	0.39863983	0.058481306	-0.9196969	197.42014	435.6867	LOC_Os02g52180.1         	uclacyanin-2 precursor	other
gb_UNIGENE10276	3.675141	0.010004934	1.3015915	5836.4375	1633.2495	3.0590618	0.011094314	1.1181083	4027.22	1368.2711	LOC_Os04g52730.1         	UDP-arabinose 4-epimerase 2	other
gb_UNIGENE2148	2.5038323	0.001338129	0.91782254	9552.6875	3833.5615	2.1504025	0.0012580421	0.76565504	7005.3647	3242.84	LOC_Os03g55070.2         	UDP-glucose 6-dehydrogenase	other
gb_UNIGENE2918	4.4468136	0.00075984164	1.4921877	24208.248	5496.491	3.8351035	0.0014646142	1.3441964	19662.926	5127.9375	LOC_Os05g29990.1         	UDP-glucuronic acid decarboxylase 1	other
gb_UNIGENE2916	2.230651	0.005909472	0.8022934	1322.925	602.51294	2.440083	0.009076228	0.892032	1403.7574	565.7798	LOC_Os07g47700.1         	UDP-glucuronic acid decarboxylase 1	other
gb_UNIGENE14054	31.417095	0.0033239445	3.4473522	744.8955	30.784584	7.976833	0.0010833666	2.0765414	1017.436	130.9587	LOC_Os03g52910.1         	uncharacterized UPF0114 domain containing protein	other
gb_UNIGENE16393	5.649648	0.0002456409	1.7315933	252.39925	44.637646	5.5127387	0.0024230452	1.7070615	629.85474	119.178955	LOC_Os01g72240.1         	uridine 5-monophosphate synthase	other
gb_UNIGENE4696	2.0851867	0.0030843935	0.7348584	8521.99	4076.6067	1.6473268	0.010803718	0.49915385	5187.6523	3185.0774	LOC_Os02g53790.2         	uridylate kinase	other
gb_UNIGENE6075	0.5445519	0.1091423	-0.607792	3245.0151	5346.7583	0.47566256	0.018716965	-0.7430466	286.83063	543.1415	LOC_Os01g12940.1         	vegetative storage protein PNI288, putative	other
gb_UNIGENE14836	0.41671553	0.029886143	-0.8753515	151.35956	353.6473	0.44261307	0.03197487	-0.81505936	46.997547	102.71742	LOC_Os06g13600.1         	VEX1	other
gb_UNIGENE5626	0.22569846	0.28043973	-1.4885554	12.386325	29.941265	0.036760587	0.02278664	-3.303329	-1.6102347	24.356335	LOC_Os08g44270.1         	vignain precursor	other
gb_UNIGENE13615	3.2892811	0.0016007479	1.1906691	5322.5996	1598.4357	3.745553	0.0003777496	1.3205693	4753.4824	1260.8579	LOC_Os02g07690.1         	VQ motif family protein	other
gb_UNIGENE15374	9.529233	0.00018967522	2.2543643	2782.9375	288.18057	6.732749	0.00024195173	1.9069835	2712.2424	395.31937	LOC_Os06g33970.1         	VQ motif family protein	other
gb_UNIGENE25977	7.5694313	0.00020234704	2.024118	1754.3425	237.14159	6.8603477	0.0020752174	1.9257581	1216.8002	180.23694	LOC_Os03g47280.1         	VQ motif family protein	other
gb_UNIGENE15874	0.45374653	0.06231382	-0.79021657	1905.9865	3720.5122	0.3739564	0.012492084	-0.983616	792.4393	1986.5258	LOC_Os11g38010.1         	WDL1	other
gb_UNIGENE6576	16.077293	0.00082008226	2.777408	21638.275	1330.543	27.510948	0.0011740661	3.314584	18533.574	734.8955	LOC_Os11g37950.1         	win2 precursor	other
gb_UNIGENE2550	5.5705667	0.00094247103	1.7174968	4203.5024	752.88654	17.222927	0.000649904	2.8462415	143471.25	8859.488	LOC_Os11g37950.1         	win2 precursor	other
gb_UNIGENE3751	0.47448385	0.103675775	-0.7455277	268.42075	543.9132	0.4972166	0.0023982935	-0.6987295	169.7215	339.1958	LOC_Os02g51710.1         	wound/stress protein	other
gb_UNIGENE8308	3.6549077	0.016763791	1.2960708	71.38245	22.254406	4.193395	0.028272118	1.4335107	77.74147	20.342575	LOC_Os02g17150.1         	xylem serine proteinase 1 precursor	other
gb_UNIGENE2672	0.3596427	0.036209345	-1.0226442	4105.02	11707.99	0.4130573	0.0047472613	-0.884169	4480.2803	10992.67	LOC_Os06g48180.1         	xyloglucan endotransglucosylase/hydrolase protein 23 precursor	other
gb_UNIGENE2673	0.4352415	0.07894368	-0.8318542	3189.7585	6063.537	0.23271975	0.00023521502	-1.4579203	801.6807	3477.5625	LOC_Os06g48200.1         	xyloglucan endotransglucosylase/hydrolase protein 23 precursor	other
gb_UNIGENE2669	0.491415	0.06498285	-0.7104663	205.73175	473.05466	0.32863832	0.007031709	-1.1127975	62.598	186.64087	LOC_Os06g48200.1         	xyloglucan endotransglucosylase/hydrolase protein 23 precursor	other
gb_UNIGENE2670	0.2791732	0.049870286	-1.2759229	3270.8928	9523.4375	0.17290059	0.0072137467	-1.7550385	836.95496	4172.2275	LOC_Os06g48200.1         	xyloglucan endotransglucosylase/hydrolase protein 23 precursor	other
gb_UNIGENE5258	0.14417633	0.03971694	-1.9367182	129.66273	700.2883	0.09445398	0.009548708	-2.3596425	22.631203	188.45416	LOC_Os11g33270.1         	xyloglucan endotransglucosylase/hydrolase precursor	other
gb_UNIGENE11615	9.2667	0.0006387162	2.2264273	2525.55	285.32962	6.2936335	0.00063791784	1.8395386	2191.5076	351.40863	LOC_Os01g64470.1         	YLS9	other
gb_UNIGENE12869	0.47546104	0.03028643	-0.7434704	134.66162	241.41551	0.51695853	0.054912534	-0.6597926	104.75233	174.34549	LOC_Os09g29130.1         	ZF-HD protein dimerisation region containing protein	other
gb_UNIGENE19858	0.33986363	0.022027712	-1.0792109	56.526474	156.15604	0.4205798	0.00888288	-0.86612105	72.8911	163.7522	LOC_Os04g35500.1         	ZF-HD homeobox protein	other
gb_UNIGENE12158	0.46724138	0.006374347	-0.7609093	742.26447	1569.6454	0.56004304	0.016365834	-0.5797416	438.55298	771.9718	LOC_Os09g24820.1         	ZF-HD homeobox protein	other
gb_UNIGENE13562	10.961451	0.00000053158493	2.3943846	11068.848	1007.72424	5.161828	0.00087457674	1.6412908	10241.112	1988.4824	LOC_Os01g48620.1         	expressed protein	unknown
gb_UNIGENE8002	0.44065857	0.034790795	-0.81948495	244.02774	571.9338	0.65565425	0.015508865	-0.42212164	266.311	408.219	LOC_Os03g07590.1         	expressed protein	unknown
gb_UNIGENE4066	0.33835444	0.00006516951	-1.0836613	97.50182	288.18488	0.2857379	0.0141003085	-1.2526803	100.22312	363.3661	LOC_Os04g49680.1         	expressed protein	unknown
gb_UNIGENE2170	18.1078	0.00013410563	2.8963428	126119.75	7081.6226	22.720106	0.00046170224	3.1232502	117787	5512.23	LOC_Os06g29730.1         	expressed protein	unknown
gb_UNIGENE17563	15.061327	0.00019513555	2.7121303	2874.6326	189.53174	10.234276	0.00058087317	2.3257425	3203.2002	319.48297	LOC_Os03g15080.1         	expressed protein	unknown
gb_UNIGENE4062	0.31773013	0.000236067	-1.1465529	857.3038	2707.2957	0.35857797	0.007592134	-1.0256091	1265.7301	3651.0125	LOC_Os04g49680.1         	expressed protein	unknown
gb_UNIGENE5351	2.976905	0.00024919878	1.0908842	7069.5747	2394.4072	3.1102176	0.0049869004	1.1346927	4229.0674	1385.7821	LOC_Os03g50870.1         	expressed protein	unknown
gb_UNIGENE813	4.4407887	0.00025833404	1.490832	8643.3125	1967.5925	5.8665648	0.00060281216	1.7692692	13023.45	2262.0115	LOC_Os06g29730.1         	expressed protein	unknown
gb_UNIGENE16700	5.3876834	0.00026818877	1.6841155	54.468075	10	4.0793157	0.004232491	1.4059292	42.6262	10	LOC_Os01g24420.1         	expressed protein	unknown
gb_UNIGENE5302	3.8302915	0.00028189167	1.3429409	10931.114	2848.3564	3.5345893	0.00090578734	1.2625971	8012.2026	2254.0698	LOC_Os08g10500.2         	expressed protein	unknown
gb_UNIGENE2163	16.571083	0.00036473756	2.8076591	83710.25	5121.0806	20.950798	0.00065454835	3.0421767	143660	7273.6543	LOC_Os06g29730.1         	expressed protein	unknown
gb_UNIGENE20750	3.228001	0.00047318757	1.1718631	2181.795	679.67004	2.9603677	0.0037706424	1.0853134	1805.5275	596.6177	LOC_Os03g47060.1         	expressed protein	unknown
gb_UNIGENE19086	7.354924	0.0004840228	1.99537	488.128	66.459625	6.340585	0.008033875	1.846971	320.75125	49.17733	LOC_Os06g22330.1         	expressed protein	unknown
gb_UNIGENE16046	4.4478283	0.00048436204	1.4924159	1695.7424	383.0383	3.7466533	0.0010484946	1.320863	1330.7225	344.29315	LOC_Os01g48360.2         	expressed protein	unknown
gb_UNIGENE5106	3.4617615	0.00050917995	1.2417775	1224.87	354.84277	3.4209979	0.0019937963	1.2299323	1483.3599	431.32452	LOC_Os10g35770.1         	expressed protein	unknown
gb_UNIGENE20159	12.282276	0.0006582804	2.5081573	141.82637	10.779241	11.17698	0.0004927634	2.4138563	117.429726	10.177467	LOC_Os01g29280.1         	expressed protein	unknown
gb_UNIGENE5303	5.4438725	0.00082237093	1.6944907	552.3107	102.316765	3.9832695	0.014303523	1.382103	572.1958	156.6086	LOC_Os08g10500.2         	expressed protein	unknown
gb_UNIGENE18427	3.9559789	0.0008863811	1.375228	6497.0376	1675.7306	3.3953364	0.0015844856	1.2224028	9112.567	2673.2815	LOC_Os09g04310.1         	expressed protein	unknown
gb_UNIGENE14713	2.0725887	0.0010353156	0.72879845	1480.0825	714.04706	2.044458	0.0010665057	0.7151327	1324.0725	649.2249	LOC_Os01g15029.1         	expressed protein	unknown
gb_UNIGENE20556	3.4682717	0.0012657042	1.2436564	519.7487	148.82936	2.786138	0.0076264893	1.0246564	472.7185	171.80034	LOC_Os12g32190.1         	expressed protein	unknown
gb_UNIGENE599	2.8723195	0.0013624072	1.0551199	968.0873	334.9687	2.1782265	0.0032691415	0.77851105	568.6285	264.2383	LOC_Os06g37020.1         	expressed protein	unknown
gb_UNIGENE6085	5.0940495	0.001548243	1.6280731	354.25174	69.72641	5.140894	0.0010340468	1.6372269	233.15302	45.244064	LOC_Os03g51430.2         	expressed protein	unknown
gb_UNIGENE21196	2.4447036	0.0016871219	0.8939239	330.21924	133.21863	2.4245284	0.012006534	0.885637	570.02246	240.18448	LOC_Os06g43810.1         	expressed protein	unknown
gb_UNIGENE19265	3.05616	0.002006727	1.1171592	124.4019	41.450233	2.0589614	0.029332057	0.72220165	74.525055	39.087353	LOC_Os05g35970.1         	expressed protein	unknown
gb_UNIGENE18399	8.735835	0.0020591659	2.1674335	1899.4575	240.00888	6.8415856	0.019919815	1.9230195	248.0865	47.147964	LOC_Os02g28970.1         	expressed protein	unknown
gb_UNIGENE6495	2.7681377	0.0021042489	1.0181748	4428.31	1607.6643	2.8028305	0.00033439233	1.0306298	3637.91	1290.067	LOC_Os01g70470.1         	expressed protein	unknown
gb_UNIGENE4389	2.6979647	0.0023264168	0.9924977	1902.5325	711.53613	2.758286	0.0066295103	1.0146095	1962.1576	727.447	LOC_Os11g44870.2         	expressed protein	unknown
gb_UNIGENE9874	3.157432	0.0023326776	1.149759	6537.6553	2050.5957	3.5749526	0.00022289951	1.2739519	5397.93	1494.3696	LOC_Os10g39920.1         	expressed protein	unknown
gb_UNIGENE15548	5.6189256	0.0027288944	1.7261405	2430.7725	443.50592	7.228008	0.00020101015	1.9779634	1435.4299	193.60492	LOC_Os05g25210.1         	expressed protein	unknown
gb_UNIGENE15613	2.249138	0.0028688344	0.8105471	1316.5266	583.06055	2.0131938	0.0187123	0.69972247	974.31775	479.48074	LOC_Os11g31960.1         	expressed protein	unknown
gb_UNIGENE11805	4.2295704	0.0029405055	1.4421004	147.08026	35.04403	2.5725608	0.019881425	0.9449018	181.472	80.1761	LOC_Os03g52410.1         	expressed protein	unknown
gb_UNIGENE15773	2.2953107	0.003540072	0.8308682	271.18576	117.32489	2.6959808	0.0034147524	0.9917621	599.41876	220.87848	LOC_Os06g44190.1         	expressed protein	unknown
gb_UNIGENE8822	2.0289648	0.0038515911	0.7075257	1306.6	648.23035	2.0634024	0.0015641454	0.7243563	1735.7324	839.2572	LOC_Os06g19690.1         	expressed protein	unknown
gb_UNIGENE939	5.987238	0.004007033	1.7896302	204616.5	36715.164	7.9045486	0.000635793	2.0674384	192689.75	24884.363	LOC_Os06g29730.1         	expressed protein	unknown
gb_UNIGENE6797	3.9918993	0.0040494986	1.3842671	18886.125	4710.822	4.042143	0.002150934	1.396775	17708.8	4262.712	LOC_Os02g36940.1         	expressed protein	unknown
gb_UNIGENE5805	2.2489865	0.0042879726	0.81047964	14514.35	6566.0347	2.2856524	0.011935802	0.8266515	10563.457	4662.2554	LOC_Os01g63210.1         	expressed protein	unknown
gb_UNIGENE10096	3.7611675	0.0046281065	1.3247294	84.49397	22.866255	3.4602327	0.0011305206	1.2413359	513.2225	151.64734	LOC_Os03g02300.1         	expressed protein	unknown
gb_UNIGENE7378	3.567156	0.0048287576	1.2717687	1702.0226	498.31744	4.691048	0.0017437594	1.5456561	1138.692	242.34192	LOC_Os05g11900.1         	expressed protein	unknown
gb_UNIGENE19171	4.039739	0.0049587884	1.3961802	457.6845	121.00199	2.9218364	0.0030742378	1.0722123	265.68524	89.377014	LOC_Os03g52380.1         	expressed protein	unknown
gb_UNIGENE10615	2.4084315	0.005148983	0.87897575	1399.5375	576.96716	2.922221	0.004404947	1.072344	1058.8475	371.5065	LOC_Os03g07400.1         	expressed protein	unknown
gb_UNIGENE6742	2.4967222	0.0060041435	0.9149788	558.6598	229.53636	2.953003	0.00062239275	1.0828226	248.33426	84.24548	LOC_Os02g36530.1         	expressed protein	unknown
gb_UNIGENE16098	0.2731337	0.0075351	-1.2977939	468.1867	1612.1523	0.3182812	0.009418349	-1.14482	347.04077	1046.8562	LOC_Os02g38970.2         	expressed protein	unknown
gb_UNIGENE26105	2.2128782	0.007618012	0.7942940000000001	2056.1123	910.32117	2.625034	0.009127123	0.96509385	1437.3125	570.77	LOC_Os01g19420.1         	expressed protein	unknown
gb_UNIGENE15650	2.419796	0.007657774	0.88368326	241.25099	101.03134	2.421267	0.007875975	0.88429093	206.61925	84.30322	LOC_Os08g34800.1         	expressed protein	unknown
gb_UNIGENE6082	3.4709647	0.009366233	1.2444326	1596.0675	465.7522	2.6522865	0.012345068	0.97542214	5657.4404	2208.1626	LOC_Os03g51430.2         	expressed protein	unknown
gb_UNIGENE13990	0.4534183	0.010406194	-0.79094017	7295.662	15530.066	0.49279803	0.004279836	-0.70765585	7275.72	14737.438	LOC_Os03g47060.1         	expressed protein	unknown
gb_UNIGENE2474	0.28052065	0.010797067	-1.2711079	102.967926	325.9348	0.3202297	0.0006714944	-1.1387167	42.883526	136.02301	LOC_Os01g42520.1         	expressed protein	unknown
gb_UNIGENE7450	0.40654668	0.011031978	-0.9000565	138.5307	333.2161	0.43315688	0.024532171	-0.8366553	208.12973	436.80557	LOC_Os09g26370.1         	expressed protein	unknown
gb_UNIGENE6505	0.34761852	0.011466368	-1.0566496	14871.191	38881.93	0.25684533	0.021424882	-1.3592812	8489.758	29276.309	LOC_Os03g17790.1         	expressed protein	unknown
gb_UNIGENE13516	2.9579396	0.012485474	1.0844929	219.11475	75.91095	2.0633988	0.015668772	0.72435457	515.2555	257.26306	LOC_Os01g41770.1         	expressed protein	unknown
gb_UNIGENE13932	2.6807196	0.013406588	0.9860853	88.6221	35.540688	3.7154021	0.0023568608	1.3124869	66.55915	18.141964	LOC_Os07g02460.1         	expressed protein	unknown
gb_UNIGENE13177	2.3038926	0.013800327	0.8346001	179.452	78.95354	2.1141083	0.0006833069	0.74863315	345.6245	162.86145	LOC_Os04g43824.1         	expressed protein	unknown
gb_UNIGENE14777	3.8479283	0.015639713	1.3475349	237.99974	64.15918	4.5818777	0.02268727	1.5221089	339.924	74.04215	LOC_Os01g72360.1         	expressed protein	unknown
gb_UNIGENE12476	0.42725807	0.019448007	-0.85036707	365.626	856.2649	0.4703848	0.0021663622	-0.75420415	299.2185	631.1771	LOC_Os02g44710.1         	expressed protein	unknown
gb_UNIGENE2475	0.22085105	0.020809552	-1.5102668	2986.0925	10905.739	0.20904657	0.0021397527	-1.5651982	1041.754	4594.4795	LOC_Os01g42520.1         	expressed protein	unknown
gb_UNIGENE8809	0.4270062	0.021424487	-0.85095674	894.8213	1959.7094	0.45143747	0.00860767	-0.7953184	711.5265	1550.0333	LOC_Os01g39000.2         	expressed protein	unknown
gb_UNIGENE16125	0.45341083	0.024813179	-0.7909566	1928.4475	3825.2944	0.46236926	0.011416614	-0.77139145	1714.51	3526.6138	LOC_Os03g11230.1         	expressed protein	unknown
gb_UNIGENE15357	0.3569254	0.025775729	-1.0302285	439.20502	1108.1797	0.35624585	0.015394426	-1.0321342	258.4803	658.2606	LOC_Os01g66980.1         	expressed protein	unknown
gb_UNIGENE15603	0.24562299	0.026134083	-1.4039575	136.71248	619.4354	0.2350898	0.02876073	-1.4477878	206.30493	786.77234	LOC_Os07g41200.1         	expressed protein	unknown
gb_UNIGENE15356	0.37160066	0.02887764	-0.9899355	964.42126	2368.2834	0.39074004	0.015273524	-0.9397128	1794.87	4252.918	LOC_Os01g66980.1         	expressed protein	unknown
gb_UNIGENE26080	2.5481772	0.03409621	0.93537825	87.8919	41.539337	3.4199774	0.029484225	1.2296339	49.694374	11.676165	LOC_Os02g22160.1         	expressed protein	unknown
gb_UNIGENE9048	0.2576123	0.041375294	-1.3562996	1399.739	4148.9062	0.26190284	0.025325937	-1.3397816	531.40326	1563.8866	LOC_Os03g60509.2         	expressed protein	unknown
gb_UNIGENE10085	2.0180805	0.0003874334	0.70214677	1488.7725	734.5006	1.7255985	0.0019160851	0.54557395	1261.61	729.6107	LOC_Os03g49960.3         	expressed protein	unknown
gb_UNIGENE2849	2.2269247	0.00055817666	0.80062157	10547.961	4735.5234	1.9336367	0.0016213424	0.6594025	9236.065	4740.5996	LOC_Os01g03760.1         	expressed protein	unknown
gb_UNIGENE15070	2.1581295	0.0010942109	0.76924187	31736.25	14679.447	1.4123727	0.0840014	0.34527105	28542.275	21161.783	LOC_Os06g20820.1         	expressed protein	unknown
gb_UNIGENE26386	2.9703412	0.0013246997	1.0886768	198.96675	66.305756	1.6966414	0.03604364	0.52865064	105.75313	59.476746	LOC_Os06g43780.1         	expressed protein	unknown
gb_UNIGENE11993	0.27699828	0.0017473465	-1.283744	180.47972	688.46106	0.630511	0.09682885	-0.46122468	119.14082	188.33205	LOC_Os04g37760.1         	expressed protein	unknown
gb_UNIGENE15316	2.3191454	0.0019432927	0.84119874	7793.1	3375.5342	1.8293844	0.020299667	0.6039795	5854.0425	3216.273	LOC_Os06g40210.1         	expressed protein	unknown
gb_UNIGENE10826	0.4911816	0.0027937305	-0.7109413	2076.4248	4153.2256	0.7133677	0.010145735	-0.33775824	3880.14	5449.483	LOC_Os03g10180.2         	expressed protein	unknown
gb_UNIGENE10573	2.449051	0.0050775087	0.8957006	14986.449	6088.4287	1.7402152	0.0042544235	0.5540088	13093.9	7544.8574	LOC_Os06g05740.1         	expressed protein	unknown
gb_UNIGENE14073	0.41831782	0.005516672	-0.8715138	1411.995	3450.2422	0.63371843	0.034664776	-0.4561505	1643.415	2617.4438	LOC_Os05g08800.1         	expressed protein	unknown
gb_UNIGENE11406	0.49956268	0.0058308956	-0.6940222	1974.7725	3812.886	0.5256228	0.016381849	-0.6431715	1452.68	2740.7676	LOC_Os02g37600.1         	expressed protein	unknown
gb_UNIGENE7210	2.4631467	0.006811373	0.90143967	17322.574	7027.1826	1.8407376	0.004350769	0.6101664	8372.86	4533.2056	LOC_Os03g55776.1         	expressed protein	unknown
gb_UNIGENE23817	0.4985324	0.0068556895	-0.69608665	549.3013	1128.4984	0.8288075	0.2320229	-0.18776739	869.63324	1078.8203	LOC_Os01g07970.1         	expressed protein	unknown
... (truncated at 1000 rows)

## Sheet: Sheet2

## Sheet: Sheet3
